# fdrci: FDR confidence interval selection and adjustment for large-scale hypothesis testing

**DOI:** 10.1093/bioadv/vbac047

**Published:** 2022-06-13

**Authors:** Joshua Millstein, Francesca Battaglin, Hiroyuki Arai, Wu Zhang, Priya Jayachandran, Shivani Soni, Aparna R Parikh, Christoph Mancao, Heinz-Josef Lenz

**Affiliations:** Department of Population and Public Health Sciences, Keck School of Medicine of USC, Los Angeles, CA 90033, USA; Division of Medical Oncology, Norris Comprehensive Cancer Center, Keck School of Medicine of USC, Los Angeles, CA 90033, USA; Division of Medical Oncology, Norris Comprehensive Cancer Center, Keck School of Medicine of USC, Los Angeles, CA 90033, USA; Division of Medical Oncology, Norris Comprehensive Cancer Center, Keck School of Medicine of USC, Los Angeles, CA 90033, USA; Division of Medical Oncology, Norris Comprehensive Cancer Center, Keck School of Medicine of USC, Los Angeles, CA 90033, USA; Division of Medical Oncology, Norris Comprehensive Cancer Center, Keck School of Medicine of USC, Los Angeles, CA 90033, USA; Division of Hematology and Oncology, Massachusetts General Hospital, Boston, MA 02114, USA; Department of Medicine, Harvard Medical School, Boston, MA 02115, USA; Biomarker Development, Innovent Biologics, Suzhou 215123, China; Division of Medical Oncology, Norris Comprehensive Cancer Center, Keck School of Medicine of USC, Los Angeles, CA 90033, USA

## Abstract

**Motivation:**

Approaches that control error by applying a priori fixed discovery thresholds such as 0.05 limit the ability of investigators to identify and publish weak effects even when evidence suggests that such effects exist. However, current false discovery rate (FDR) estimation methods lack a principled approach for *post hoc* identification of discovery thresholds other than 0.05.

**Results:**

We describe a flexible approach that hinges on the precision of a permutation-based FDR estimator. A series of discovery thresholds are proposed, and an FDR confidence interval selection and adjustment technique is used to identify intervals that do not cover one, implying that some discoveries are expected to be true. We report an application to a transcriptome-wide association study of the MAVERICC clinical trial involving patients with metastatic colorectal cancer. Several genes are identified whose predicted expression is associated with progression-free or overall survival.

**Availability and implementation:**

Software is provided via the CRAN repository (https://cran.r-project.org/web/packages/fdrci/index.html).

**Supplementary information:**

Supplementary data are available at *Bioinformatics Advances* online.

## 1 Introduction

Momentum has been gaining in recent years to go beyond the dichotomous and somewhat arbitrary dictates of the *P* < 0.05 threshold for ‘statistical significance’ (Amrhein *et al.*, 2019; Wasserstein *et al.*, 2019). The main problem is that it oversells the *P*-value and can lead to true discoveries mistakenly dismissed because they fail to achieve the threshold or to unjustified confidence in weak evidence. The emerging consensus is that more nuanced approaches are needed to evaluate the totality of evidence and accept but quantify uncertainty.

In large-scale testing settings such as omics, the false discovery rate (FDR) plays an important role because it is based on a signal-to-noise approach, which is more sensitive than family wise error rate (FWER) when multiple null hypotheses are false. Unlike FWER, some noise (false discoveries) is acceptable if the signal (true discoveries) is strong in comparison. Rather than applying a predefined threshold such as 0.05, some have suggested that the set of rejected tests should be guided by the results themselves and study-specific factors such as the costs of following up on false discoveries versus the benefits of identifying true discoveries (Goeman and Solari, 2011).

With the development of the ‘q-value’, an FDR estimate using an observed *P*-value as a discovery threshold, a shift in thinking has occurred among some statisticians from conceptualizing FDR as an entity that should be controlled to a parameter that can be estimated (Storey and Tibshirani, 2003). It is underappreciated that like all point estimates the informativeness of an estimate of FDR is inversely related to its variance, and study-related factors such as the number of discoveries can substantially affect the variance of the estimate (Millstein and Volfson, 2013). A measure of uncertainty is therefore needed for proper interpretation. Millstein and Volfson (MV) addressed this problem by developing a permutation-based estimator with corresponding confidence intervals (CIs) (Millstein and Volfson, 2013).

To choose a discovery threshold without the conventional 0.05 constraint, MV proposed computing FDR and corresponding CIs for multiple candidate thresholds, then selecting from among them. However, a remaining problem, as Benjamini and Yekutieli (BY) showed (Benjamini and Yekutieli, 2005b), is that intervals selected because they imply departure from the null hypothesis may fail to provide the assumed coverage probability.

Here we describe how a technique developed by BY for adjusting multiple intervals for selected parameters (Benjamini and Yekutieli, 2005b) can be adapted to address the FDR CI selection problem. Other authors have proposed FDR estimation and CIs for fixed rejection regions (Scheid and Spang, 2005; Storey, 2002), and simultaneous upper bounds for the false discovery proportion (FDP) (Katsevich and Ramdas, 2020; Meinshausen, 2006). However, this is the first principled approach we are aware of for *post hoc* selection and adjustment of FDR CIs.

We include an application to the MAVERICC clinical trial (Parikh *et al.*, 2019), a study of metastatic colorectal cancer (CRC) patients treated with first-line chemotherapy consisting of modified leucovorin, 5-fluorouracil, oxaliplatin plus bevacizumab (mFOLFOX6-BV) or leucovorin, 5-fluorouracil, irinotecan plus bevacizumab (FOLFIRI-BV). A transcriptome-wide association study (TWAS) was conducted to investigate the relationship between genetic regulated gene expression and progression-free survival (PFS) or overall survival (OS).

## 2 Materials and methods

Suppose a multiple testing setting with *m* hypotheses tests, $H_{1},\ldots ,H_{m}$, with corresponding *P*-values, $P_{1},\ldots ,P_{m}$. An investigator selected threshold, $P^{*}$, defines *S* rejected tests that includes *F* false discoveries (true null hypotheses) and *S–F* true discoveries (false null hypotheses). Then FDR as estimated by MV, sometimes referred to as pFDR, is defined as E[F/S|S>0], where *F*/*S* is the FDP. Here *S* is observed once $p^{*}$ is chosen; however, *F* (and thus FDR) is unknown based on the data at hand and can only be estimated.

MV FDR estimation (Supplementary Material) provides CIs and has several other attractive features; (i) permutation-based and thus non-parametric, accommodating statistics with unknown distributions, without need for *P*-values as intermediate statistics; (ii) accounts for the number of permutations conducted, thus indicating convergence to asymptotic values; (iii) accounts for general dependencies among tests; (iv) conservative, but less conservative than Benjamini and Hochberg (1995) (BH) FDR; and (v) variance of log(${\hat{\mathrm{FDR}}}_{t}$) is smaller than the Storey and Tibshirani (2003) approach. MV FDR can be used whenever realizations of the statistic of interest can be generated under the null hypothesis by permutation and where that distribution is similar across tests (approximate exchangeability).

### 2.1 FDR-adjusted selected intervals

The BY is a general approach that can control the false coverage-statement rate (FCR) through selection and adjustment of intervals associated with rejected null hypotheses by leveraging BH FDR. They define FCR as the expected proportion of parameters not covered by their intervals among the selected parameters and where the proportion is defined to be zero if no parameters are selected. Let *S* here be the total number of selected CIs and *F* the number of these not covering their respective parameters, then FCR=E[Q], where,

(1) $$Q=\{\begin{array}{cc} F/S & \text{if}\;S>0\\ 0 & \text{otherwise}. \end{array}$$

Here we adapt the BY approach for selecting and adjusting MV FDR intervals at a series of *T* investigator defined thresholds, where the parameters are log-transformed FDR point estimates at those thresholds. Our interest is only in discovery sets with sufficient evidence that *FDR* < 1, which is the alternative to the composite null hypothesis that all discoveries are false. The investigator is then able to make an informed choice from among the selected rejection regions.

The BY approach requires a *P*-value for each parameter. Hence, for each threshold, *t*, we propose a *P*-value based on a one-sided Wald test, *P_t_*, computed from the MV FDR estimate and standard error (Supplementary Equations 1 and 2), where,

(2) $$P_{t}=\Phi (Z_{t}),\;Z_{t}=\frac{\;\text{log}({\hat{\mathrm{FDR}}}_{t})}{\sigma_{\;\text{log}(FDR_{t})}},Z∼N(0,1).$$

Here ϕ denotes the standard normal cumulative distribution function, and where the hypotheses are, $H_{0}:\;\text{log}(FDR_{t})=0$ vs. $H_{a}:\;\text{log}(FDR_{t})<0$. The steps of the adapted BY approach are described in Algorithm 1.

**Algorithm 1** Select and adjust MV FDR confidence intervals **Require:** set of p-value thresholds, T, defining nested candidate rejection regions

1: **for**t∈T**do**

2: Compute p-values, $P_{t}=\Phi (Z_{t})$

3: **end for**

4: Sort |T| p-values from smallest to largest, $P_{\left(1\right)},\ldots ,P_{\left(|T|\right)}$

5: Calculate $R=\text{max}\{i:P_{\left(i\right)}<i\alpha /|T|\}$

6: **for**r≤R**do**

7: $CI_{r}=\text{exp}\;\{\;\text{log}({\hat{\mathrm{FDR}}}_{r})\pm z_{\frac{\alpha^{*}}{2}}\sigma_{\;\text{log}(FDR_{r})}\}$, where $\alpha^{*}=R\alpha /|T|$

8: **end for**

9: Return $CI_{r}\;\forall \;r\leq R$

BY proved (Theorems 1 and 3 (Benjamini and Yekutieli, 2005b)) that an approach with the structure of Algorithm 1 controls FCR at level *α* as long as tests are independent or are positive regression dependent on a subset (PRDS) and the marginal intervals have the assumed coverage. While the proposed *P*-values are not independent, they likely satisfy the PRDS criterion due to positive correlations of overlapping rejection regions. Therefore, selected thresholds will have MV FDR intervals corrected for the effects of selection such that coverage is FCR controlled under typical conditions.

Implemented in the fdrci package, the software was designed to accommodate arbitrarily large-scale analyses using the property that MV FDR requires only the total number of tests, *m*, and counts of rejected tests in the observed and permuted results. Thus, computational requirements are minimized by allowing the user to filter out results that are not likely to be of interest (e.g. *P*-values larger than candidate thresholds) prior to FDR analysis. The R code used for the analyses here is provided in Supplementary Material in the R Markdown format, and a vignette, ‘fdrci: FDR selection and adjustment—HGSOC’ has been added to the fdrci software for instruction.

### 2.2 Numeric simulation

We conducted computer simulations under a variety of scenarios to compare coverage of MV FDR intervals following three selection strategies: (i) ‘UCB’, upper CI bound <1, (ii) ‘M.2’, MV FDR point estimate <0.2 and (iii) ‘BY’, proposed approach. For each replicate of each scenario, 100 block-correlated Gaussian variables were simulated with sample size 200 and block size five. For each block, $X_{l}∼\mathrm{MVN}(0,\Sigma_{l})$,

(3) $$\Sigma_{l}=\left[\begin{array}{ccc} \sigma^{2} & \sigma^{2}\rho & \ldots \\ \sigma^{2}\rho & \ddots & \vdots \\ \vdots & \ldots & \sigma^{2} \end{array}\right],$$

where $X_{l}$ denotes predictors in block *l*, all correlated at ρ=0.3. For each of the *L *=* *20 blocks, an outcome variable, $Y_{l}∼N(X_{l}\beta ,\sigma^{2})$, was generated, yielding 20 outcome variables in all. The objective of the analysis for each replicate was to identify true associations using hypothesis tests, $H_{0}:\beta =0$, for all predictor-outcome pairs, a total of 100 × 20 = 2000 tests. To apply MV FDR, 20 additional analyses were conducted for each data set after randomly permuting outcome variables with respect to predictors, thereby maintaining the dependencies within predictor and outcome sets. MV FDR and intervals were computed from the observed and permutation *P*-values for a series of significance thresholds, $-{\;\text{log}\;}_{10}(P)=2.0,2.1,\ldots ,6.0$, and intervals were selected (and adjusted for the BY approach) according to the three approaches described above.

Two thousand replicate studies were conducted for each of five scenarios: (A) 5 blocks with β=[0.05,0.05,0.05,0.05,0.05]′ and 15 blocks with β=[0,0,0,0,0]′, thus $H_{0}$ false for 25 of the 2000 tests (β here refers to the equation for *Y_l_* above), (B) like Scenario A, but with $H_{0}$ false for 10 blocks or 50 of 2000 tests, (C) like Scenario A but with a larger effect under $H_{a},\;\beta =[0.075,0.075,0.075,0.075,0.075]\prime$, (D) like Scenario C, but with $H_{0}$ false for 10 blocks and (E) β=[0,0,0,0,0]′ for all blocks, thus E represents the global null of no associations between any predictors and outcomes. To estimate FCR, *Q* (Equation (1)) was computed for the selected intervals for each replicate, with the mean computed across all replicates.

To assess the statistical properties of the permutation approach for detecting statistical interactions in Cox proportional hazards models, we conducted computer simulations under a variety of scenarios (Supplementary Material) consistent with Model 3 (below). These included a sample size of 329 as observed in the MAVERICC data, a binary treatment variable, a continuous or categorical gene variable, and a correlated adjustment covariate (*r* ∼0.6). The objective was to determine whether evidence supports concerns expressed by previous authors that basic permutation-based approaches are more problematic for detecting statistical interactions (Anderson and Braak, 2003).

### 2.3 Application to the MAVERICC clinical trial

We conducted a TWAS in the MAVERICC clinical trial to identify genes predictive or prognostic of PFS or OS. MV FDR with the BY approach was used to identify discoveries and quantify uncertainty. For comparison, FDR was also computed using the BH method.

MAVERICC was a randomized phase II trial comparing first-line treatment with modified leucovorin, 5-fluorouracil, oxaliplatin plus bevacizumab (mFOLFOX6-BV) to treatment with leucovorin, 5-fluorouracil, irinotecan plus bevacizumab (FOLFIRI-BV) in patients with previously untreated metastatic CRC (Parikh *et al.*, 2019).

Genome-wide association study data for this study were generated by the Illumina Infinium OncoArray-500K BeadChip, designed primarily for measuring genomic variation associated with predisposition to common cancers (Amos *et al.*, 2017). QC steps included the following. Samples with call rates <95% or with discordant genotype imputed vs. reported sex were excluded. Markers were excluded if call rate was <98% or if the within-race Hardy–Weinberg equilibrium (HWE) *P*-value was <$10^{-12}$. The strict HWE criterion reflects the possibility of a departure from HWE caused by case-status rather than by genotyping errors.

Genotype imputation was conducted for the autosomes, excluding single nucleotide polymorphisms (SNPs) with minor allele frequency <0.01. Data were pre-phased using SHAPEIT v2.r837 and imputed to the 1KGP Phase 3 reference panel [downloaded from the IMPUTE2 website (https://mathgen.stats.ox.ac.uk/impute/impute_v2.html#reference)] using IMPUTE v2.3.2 (Howie *et al.*, 2012). The enhanced dataset was then used to impute genetically regulated gene expression. Gamazon *et al.* (2015) analyzed transcriptome and genetic variation data from the GTEx eQTL repository to construct additive models of genetically regulated gene expression (Barbeira *et al.*, 2018; Gamazon *et al.*, 2015). They applied an elastic net approach to estimate weights for linear combinations of SNPs for transverse colon and other tissues. We used version 7 weights for transverse colon (downloaded from predictdb.org) with the imputed SNP data from the three clinical trials to impute gene expression for 5613 genes across the genome following QC. To further reduce dimensionality of the dataset, we applied the Partition approach (Barrett and Millstein, 2020; Millstein *et al.*, 2020), specifying a maximum information loss of 20% (minimum information capture of 80%). This procedure collapses highly correlated groups of features into new features, subject to the satisfaction of the information loss constraint. We observed a high degree of statistical independence between imputed gene features, thus few groups were collapsed. The final data set included 5520 imputed features, with 74 representing multiple predicted coexpressed genes. To eliminate high leverage points, each feature was transformed into an ordinal variable with three tertile groups.

OS was defined as time from randomization until death from any cause or last follow-up, and PFS was defined as time from randomization to disease progression, death or last follow-up. Adjustment covariates included age, sex, Eastern Cooperative Oncology Group (ECOG) performance status, number of metastatic sites, tumor location (left versus right), KRAS mutation status, and treatment arm. Patients with complete data included 162 from the mFOLFOX6-BV arm and 167 from the FOLFIRI-BV arm. Following the approach of the primary analysis for the MAVERICC clinical trial, models were stratified by geographic region and tumor excision repair cross-complementing group 1 (ERCC1) expression levels (high/low). Three Cox models were fitted for each gene,

(Model 1) $$\text{ln}\left(\frac{h(t)}{h_{0}(t)}\right)=\Sigma \gamma_{i}W_{i}+\beta T$$

(Model 2) $$\text{ln}\left(\frac{h(t)}{h_{0}(t)}\right)=\Sigma \gamma_{i}W_{i}+\beta T+\delta G$$

(Model 3) $$\text{ln}\left(\frac{h(t)}{h_{0}(t)}\right)=\Sigma \gamma_{i}W_{i}+\beta T+\delta G+\omega TG$$

where *h*(*t*) denotes the hazard function, *W* denotes adjustment covariates, *T* is an indicator for treatment and *G*, the imputed gene. For each gene, two likelihood ratio tests were conducted, a test of the prognostic effect, *δ* (Model 2 versus Model 1), and a test of the predictive effect (treatment-gene interaction) *ω* (Model 3 versus Model 2). One hundred permutation analyses were conducted by randomly permuting *G* within strata, where the same permutation was used for all gene features. Thus, dependencies between gene features, and between adjustment covariates and the outcomes were preserved.

## 3 Results

### 3.1 Simulation results

Departures from the assumed coverage were small and tended to be conservative, with coverage >0.95 for the three approaches for most scenarios that included false null hypotheses (Table 1). However, for the global null (Scenario E), coverage was substantially decreased for UCB (coverage = 0.780), with 22.1% of replicates including at least one CI upper bound <1. M.2 selection was also anti-conservative (coverage = 0.935), with 12.0% of replicates including at least one CI upper bound <1. In contrast, BY selection was slightly conservative (coverage = 0.969), with 4.5% of replicates including at least one CI upper bound <1. Coverage tended to be more conservative with larger effect sizes and larger numbers of false null hypotheses. The BY approach was the most conservative in all scenarios.

**Table 1.** Estimated coverage (1 − FCR) from 2000 replicate simulation studies for each of five scenarios

	FDR CI selection method		
Scenario	UCB	M.2	BY
A	0.942	0.954	0.973
B	0.965	0.969	0.979
C	0.971	0.972	0.976
D	0.973	0.978	0.976
E${}^{*}$	0.780	0.935	0.969

A: 5 non-null blocks, weak effects; B: 10 non-null blocks, weak effects; C: 5 non-null blocks, strong effects; D: 10 non-null blocks, strong effects; E${}^{*}$: 0 non-null blocks (global null hypothesis).

**Fig. 1.**
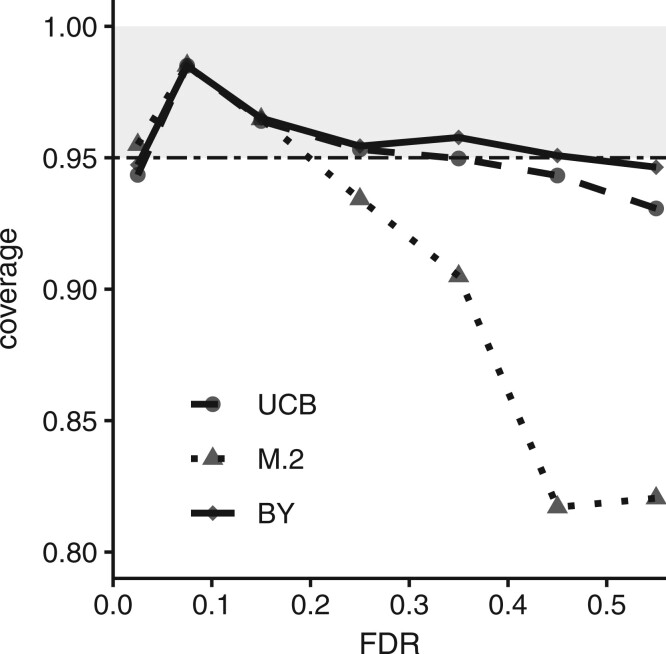
Conditional coverage for three CI selection approaches. UCB indicates selection of intervals where the upper bound is <1; the M.2 approach selects intervals where MV FDR estimate is <0.2; and BY denotes Benjamini and Yekutieli FCR-adjusted BH-selected intervals. Conditional coverage was computed across the four simulation scenarios, A–D (8000 replicates in total, including 160 000 permutation analyses), where each replicate study included a non-zero proportion of false null hypotheses (see Section 2)

Conditional coverage is a useful concept as it is restricted to selected intervals. However, BY arrived at the FCR formulation in part because conditional coverage depends on underlying conditions. They showed that even very conservative approaches such as Bonferroni adjusted intervals fail to achieve adequate conditional coverage in some situations typified by the global null. Understanding its limitations, we assessed conditional coverage for the three selection approaches as a function of true FDR. In simulations, M.2 performed substantially worse than the other approaches in the approximate range, 0.2 < true FDR < 0.5 (Fig. 1). This result is perhaps not surprising considering that within this range the interval is selected only if the FDR point estimate differs from the true value. In contrast, BY showed mostly conservative conditional coverage across the range 0 to 0.5.

The permutation approach for statistical interactions was more conservative than the parametric approach in almost all simulation scenarios (Supplementary Tables S1 and S2). Statistical power was similar for the parametric and permutation-based approaches. When both prognostic and predictive gene effects (*δ* and *ω*) were set to zero and extreme high leverage points were included, type I error was still close to assumed levels. However, high leverage points combined with outliers in the presence of main effects in G caused inflated type I error for both approaches. When high leverage points were addressed by transforming G into an ordinal variable by tertiles, the approach taken for the MAVERICC analysis here, type I error was reduced to acceptable levels. These results support the use of the permutation approach for the MAVERICC gene-treatment interaction analysis.

### 3.2 Application to the MAVERICC clinical trial

In data from the MAVERICC study, several genes were identified as discoveries both according to BH and BY criteria (Table 2). The close agreement between the parametric BH FDR (*q_BH_*) and non-parametric MV FDR (*q_MV_*) is consistent with approximate satisfaction of parametric assumptions underlying *P*-values from the Cox proportional hazards models. It also supports the validity of the permutation approach for testing treatment by gene interactions. Two genes, *WDSUB1* and *CAVIN3*, achieved transcriptome-wide selection for gene*treatment interaction by BY approach as well as FDR < 0.05 by both BH and MV. The two genes, *DYNC2H1* and *CCDC47*, did not achieve the conventional BH discovery thresholds of 0.05 or 0.1, however, they were selected by the BY approach, implying that though the evidence may be weak, it does provide some support for true discoveries. It should be noted that the evidence for these two genes is consistent between the BH and BY approaches; however, the BY FDR approach provides additional information regarding the precision of the FDR estimates. There was no evidence of prognostic effects. The minimum BH FDR was 0.76 and MV FDR did not fall below 1 for the series of significance thresholds $\left(-{\;\text{log}\;}_{10}(P)=\{3,3.1,3.2,\ldots ,6\}\right)$ tested.

**Table 2.** TWAS features associated with PFS via treatment interaction in metastatic colorectal cancer patients from the MAVERICC clinical trial

					FDR for LRTs of *ω*		
Symbol	δ(95%CI)	$P_{\delta }$	ω(95%CI)	$P_{\omega }$	*q_BH_*	*q_MV_* (95% CI)	($q_{MV_{a}}$ 95% CI)
WDSUB1	0.01 (−0.16, 0.17)	0.95	−0.78 (−1.12, −0.44)	4.7e−6	0.026	0.025 (0.005, 0.13)	(0.004, 0.14)
CAVIN3	0.14 (−0.03, 0.31)	0.12	−0.78 (−1.12, −0.43)	1.0e−5	0.028	0.025 (0.005, 0.13)	(0.004, 0.14)
DYNC2H1	1.76 (−0.81, 4.33)	0.15	12.1 (4.7, 19.4)	6.2e−5	0.11	0.13 (0.04, 0.42)	(0.04, 0.46)
CCDC47	−0.08 (−0.25, 0.09)	0.36	0.65 (0.31, 0.99)	1.5e−4	0.21	0.24 (0.09, 0.66)	(0.08, 0.70)

δ(95%CI), ω(95%CI) correspond to Model 3; *P*-values reported are for LRTs. *Q*-values correspond to FDR estimates for likelihood ratio tests of *ω*. *q_BH_* denotes BH FDR, *q_MV_* denotes MV FDR and $q_{MV_{a}}$ denotes BY adjusted CIs using 100 replicate permutations.

**Fig. 2.**
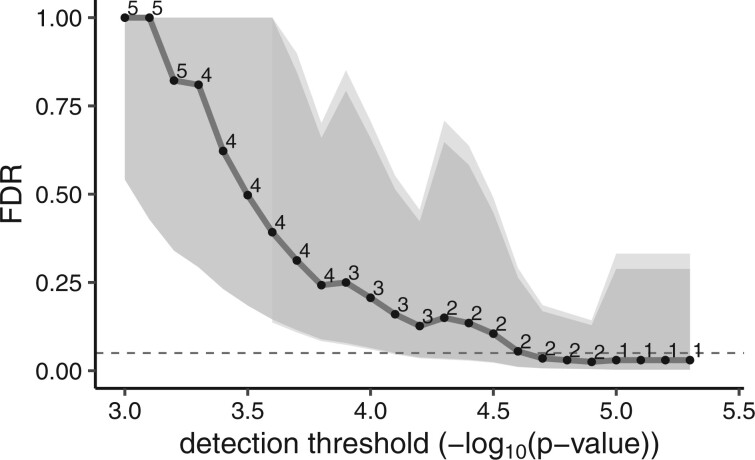
MV FDR discovery plot for tests of treatment*gene interactions, *ω*, for PFS. *P*-values from likelihood ratio tests were computed for both permuted (100 replicates) and non-permuted data to estimate MV FDR for a series of detection thresholds. Digits in the field denote the number of discoveries at each *P*-value threshold. The gray shaded area displays the 95% CI region computed by the MV FDR method whereas the light gray area represents the BY FDR adjusted approach. The dashed line indicates FDR = 0.05

Figure 2 demonstrates that MV FDR tends to decrease as the stringency of the *P*-value threshold increases (a style of plot available in the fdrci software), as we would expect. It is also apparent from Figure 2 that the cost of applying BY selection/adjustment is low in terms of increased CI width.

**Fig. 3.**
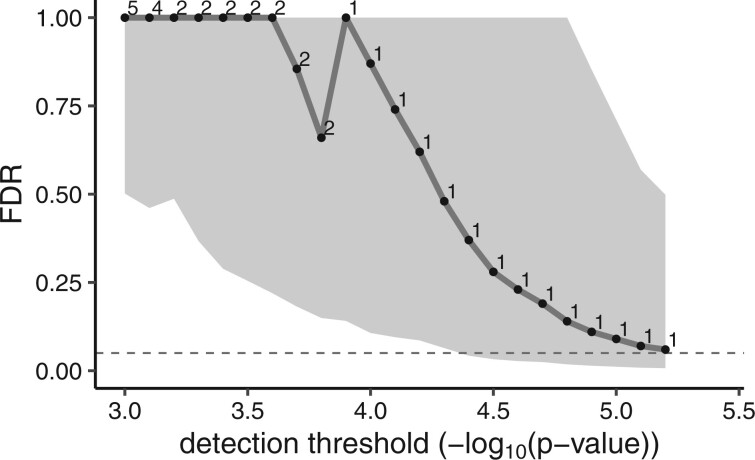
MV FDR discovery plot for tests of *δ* (Model 2), the prognostic effect of a gene on OS. *P*-values from likelihood ratio tests were computed for both permuted (100 replicates) and non-permuted data to estimate MV FDR for a series of detection thresholds. Digits in the field denote the number of discoveries at each *P*-value threshold. The gray shaded area displays the 95% CI region computed by the MV FDR method. The dashed line indicates FDR = 0.05

For OS, no effect achieved BY FDR selection, however, the prognostic effect, *δ* (Model 2), of the gene *SMIM19* achieved BH 0.05 level (P=5.1e−6). The *q_BH_* = 0.028, whereas *q_MV_* = 0.060, which again demonstrates relatively close agreement, however intervals were not selected by the BY approach. Figure 3 shows that the intervals are quite wide, which is not surprising considering that precision of the FDR estimate decreases with decreasing numbers of discoveries (Millstein and Volfson, 2013).

Kaplan–Meier curves for PFS by gene expression tertile for Table 2 genes are shown in Figure 4. The low and high expression groups tend to reverse order between treatments, consistent with predictive effects. This dynamic is especially compelling for *WDSUB1*, where median PFS in patients with high predicted expression is 18.1 months for the FOLFIRI arm but only 8.5 months for FOLFOX6. Another example is the low expression group for *DYNC2H1*, where median OS is 27.7 months for the FOLFIRI arm but only 8.1 months for FOLFOX6. In contrast, the prognostic effect on OS for *SMIM19* has a similar pattern across arms (Fig. 5).

**Fig. 4.**
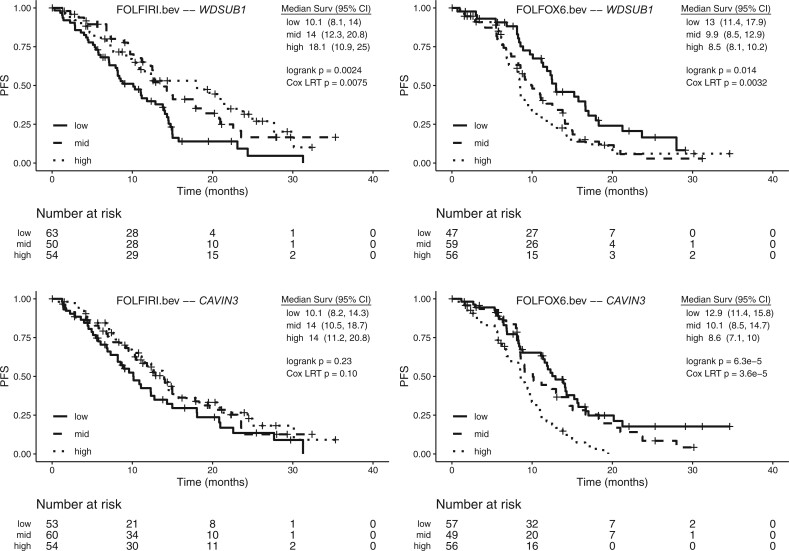
Kaplan–Meier curves for progression-free survival (PFS) for *WDSUB1,CAVIN3*, *DYNC2H1*, and* CCDC47* genes in the MAVERICC clinical trial. For each treatment arm, tertiles of patients according to their genetic imputed gene expression are denoted by low, mid and high

**Fig. 5.**
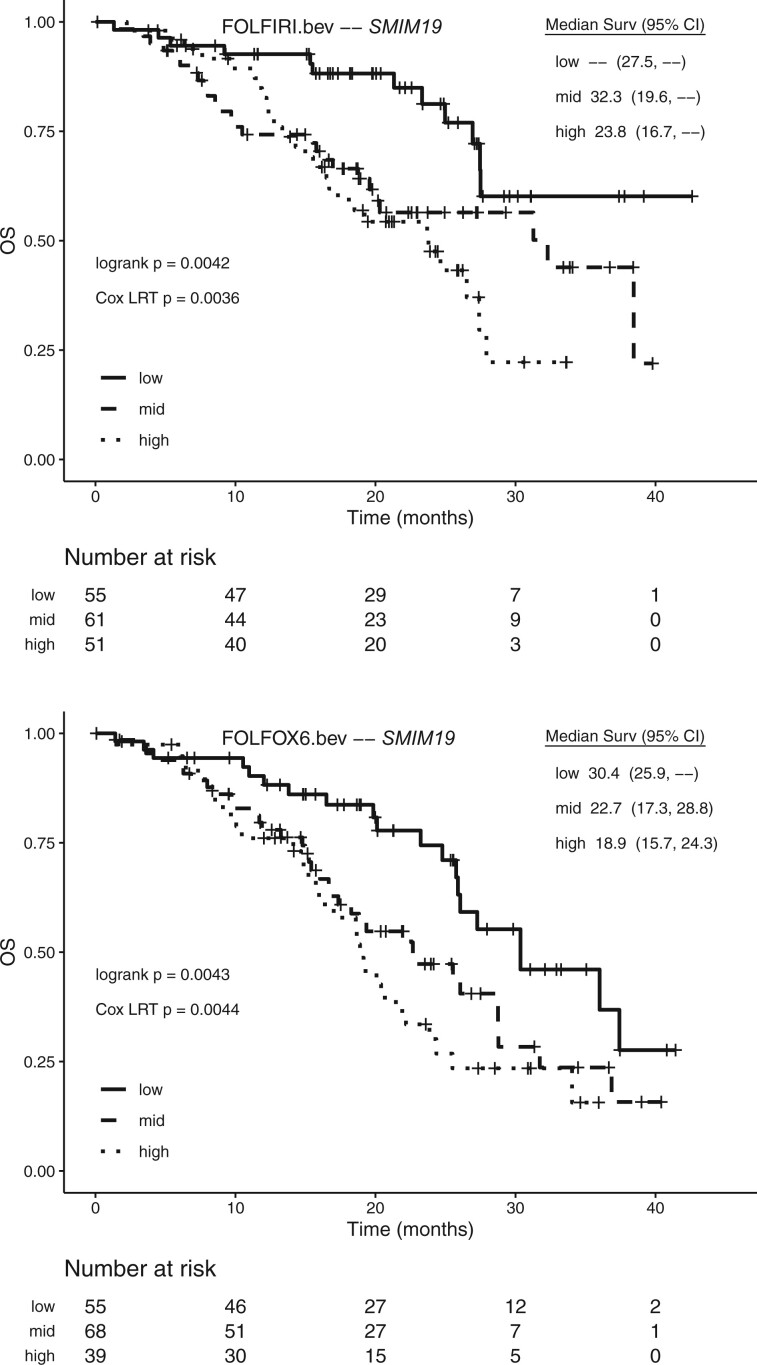
Kaplan–Meier curves for overall survival (OS) for *SMIM19* gene in the MAVERICC clinical trial. For each treatment arm, tertiles of patients according to their genetic imputed gene expression are denoted by low, mid and high

**Fig. 4.**
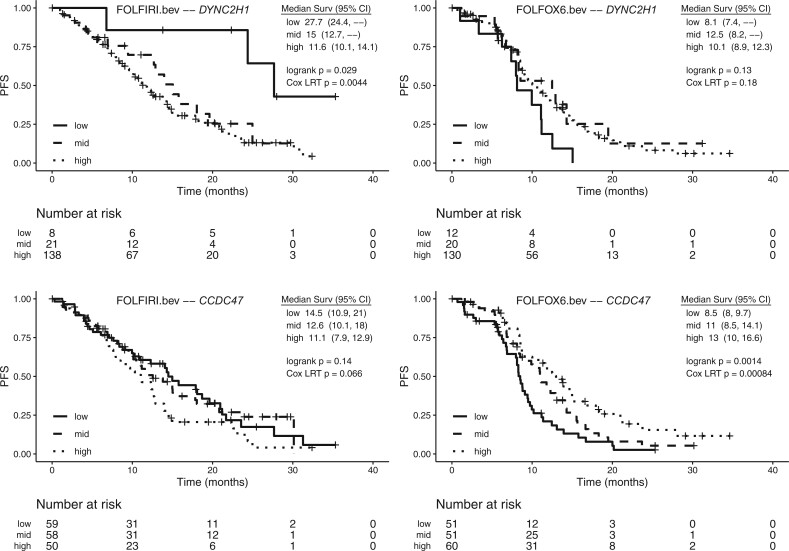
(Continued)

## 4 Discussion

In contrast to fixing the error rate before viewing the data to identify a rejection region, we suggest a dynamic process that incorporates study-specific factors such as precision of the FDR estimate, number of discoveries and cost/benefit of follow up research. The proposed adapted BY approach provides FDR CIs with the assumed coverage probability for investigator-selected discovery thresholds. If we consider each candidate threshold to be a hypothesis test, where selection of the associated interval corresponds to rejection of the test, then the proposed approach controls BH FDR. Therefore, it also controls FWER under the global null hypothesis that all null hypotheses are true (Goeman and Solari, 2014). Alternatively, if false null hypotheses are likely to be rejected, the approach ensures conditional coverage (Benjamini and Yekutieli, 2005a).

In related work, Storey (2002) proposed a resampling-based interval for *posthoc* FDR estimation, however, it does not account for the number of permutations conducted, and the problem of multiple inference was not discussed. In the context of FDP, more has been done to allow for adaptive selection of rejection regions by defining simultaneous upper confidence bounds. Meinshausen (2006) described an exact permutation-based method for defining simultaneous upper bounds on FDP, accounting for arbitrary dependencies among tests and accommodating an exploratory strategy for selection of rejection regions. Like MV FDR, this method can be applied without *P*-values as intermediate statistics. Hemerik *et al.* (2019) describe modifications to improve and generalize this approach. Goeman and Solari (2011) proposed a general technique based on the closed testing principle that provides simultaneous upper FDP bounds that hold uniformly across all rejection sets. Although this approach can be computationally expensive, there are shortcuts and simplifications, such as those described by Katsevich and Ramdas (2020). Some have argued that FDP rather than FDR should be controlled, since it is more relevant to the data set in hand. However, currently, FDR is more widely used, possibly because estimation of FDP under general dependence is much more difficult than FDR (Pawitan *et al.*, 2006), and statistical power may be lower (Katsevich and Ramdas, 2020).

We showed that the approach proposed here yields conservative coverage in simulated data and is more conservative than selecting intervals with upper bound <1 or intervals corresponding to point estimates <0.2. We demonstrated that the method of selecting intervals with upper bound <1 can be substantially anti-conservative under the global null but that the proposed approach adequately addresses this problem. We also showed that the proposed approach has much better conditional coverage under the scenarios investigated here than the approach of selecting intervals with FDR estimates <0.2. Although simulation studies were confined to a sub-region of the parameter space, the challenges of hypothesis testing with moderately strong positive dependencies among features, which translates to positive dependencies among tests, were addressed.

A limitation is that calculation of the intervals requires permutation results. For permutation statistics to be valid realizations of the null distribution, the standard assumptions underlying permutation tests apply. It has been noted in the context of linear models, that restricting permutation to a single feature of interest would break any dependencies that exist between that feature and the other covariates (Berrett *et al.*, 2020). Thus, the distribution of the test statistic under permutation may not exactly represent the true null distribution. While we do not dispute this reasoning, we note that *T* is a randomized treatment, therefore *G* is statistically independent of *T* under both the null and alternative. Further, for the MAVERICC application, we assume that the parametric properties of the test statistic approximately hold under the null hypothesis that *G* is independent of the outcome conditional on the covariates, that is, the test statistic has a chi-square distribution with one degree-of-freedom, whether or not there are dependencies between *G* and the other covariates. Under these assumptions, permuting *G* within strata would also yield a test statistic with a chi-square distribution under the null. Thus, even though there could be dependencies between the observed *G* and the other covariates, both observed and permuted test statistics would be distributed as approximately chi-square under the null.

Similar reasoning applies to the test of the interaction term, *TG*. The simulation results showed that type I error was inflated when high leverage points and outliers occurred in the presence of main effects of *G* and *T* on survival. However, it was well controlled when there was no main effect of *G* or when *G* was transformed into an ordinal variable. The permutation approach was slightly more conservative than the parametric approach for both predictive and prognostic effects, even with strong positive or negative correlations between *G* and an adjustment covariate. These results are consistent with the findings of Bůžková *et al.* (2011), which test statistics approximately independent of main effects can yield approximately valid permutation-based tests for interaction in large samples.

Notably, *CAVIN3* (Caveolae Associated Protein 3), identified here as predictive for PFS, is known to be downregulated in several cancer cell lines by genetic or epigenetic alteration and is considered a putative tumor suppressor (Hernandez *et al.*, 2013). *CAVIN3* expression is commonly lost or decreased in CRC by aberrant CpG hypermethylation and loss of function is linked to tumor progression and poor prognosis. *CAVIN3* overexpression leads to cell cycle arrest, apoptosis, suppression of colony formation *in vitro* and inhibition of tumor growth *in vivo* in CRC models. *CAVIN3* dictates the balance between ERK and Akt signaling, two key signaling pathway in CRC development and treatment response (Hernandez *et al.*, 2013). MAPK/ERK signaling promotes drug resistance by inducing epithelial–mesenchymal transition, while suppression of PI3K/AKT/mTOR pathway can reverse oxaliplatin resistance of CRC cells and increases irinotecan sensitivity. Furthermore, *CAVIN3* also interacts with *BRCA1*, and its epigenetic inactivation via promoter hypermethylation has been associated to resistance to oxaliplatin in CRC (Moutinho *et al.*, 2014). *CAVIN3* silencing occurs in primary tumors from CRC patients, where it predicts shorter PFS in oxaliplatin-treated case patients with advanced disease. Here patients with higher predicted *CAVIN3* levels had shorter PFS, an apparent discrepancy possibly explained by the differences between normal transverse colon tissue and tumor tissue.

*CCDC47* (Coiled-Coil Domain Containing 47 or Calumin) was among 8 top-ranking genes in a study focused on candidate driver genes affected by point mutations in microsatellite instable CRC (Kondelin *et al.*, 2018). The downregulation of *DYNC2H1* (Dynein Cytoplasmic 2 Heavy Chain 1) was reported in breast cancer (Kondelin *et al.*, 2018), and *DYNC2H1* is a critical gene for the assembly of primary cilia, microtubule-based organelles that protrude from the cell surface, which play a critical role in development and disease through regulation of signaling pathways including the Hedgehog pathway. Loss of cilia has been reported in several cancer types and it has been hypothesized that the presence or absence of cilia may regulate targeted drug efficacy (Hassounah *et al.*, 2012). On the other hand, *CCD47* is involved in the regulation of calcium ion homeostasis in the endoplasmic reticulum, which has been recently shown to be important in processes related to cancer progression including the development of resistance to cancer therapies (Bong and Monteith, 2018; Kerkhofs *et al.*, 2018).

Two discoveries, *CCDC47* and *DYNC2H1*, were indicated by MV FDR even though FDR was >0.05. Here the intervals played an important role in quantifying uncertainty, suggesting that despite the larger FDR, they are still likely to be true discoveries.

## Supplementary Material

vbac047_Supplementary_Data
