# Supplementary material for: fdrci: FDR confidence interval selection and adjustment for large-scale hypothesis testing
Source: Bioinform Adv. 2022 Jun 13;2(1):vbac047. doi: 10.1093/bioadv/vbac047 (PMC9210923; doi:10.1093/bioadv/vbac047)
Supplement: vbac047_Supplementary_Data [file vbac047_supplementary_data.zip › Supplemental_v10.pdf]

fdrci: FDR confidence interval selection and adjustment for large-scale hypothesis testing

Joshua Millstein, Francesca Battaglin, Hiroyuki Arai, Wu Zhang, Priya Jayachandran, Shivani Soni, Aparna R. Parikh, Christoph Mancao and Heinz-Josef Lenz

Permutation-Based Non-Parametric FDR (MV FDR) and CIs

Consider *m* hypothesis tests, each represented by a statistic *Z*, which has a common (or at least similar) distribution under all null hypotheses, H_{0i}, where *i* denotes an individual test. For ease of notation we assume without loss of generality that the test statistic is rejected in the lower tail only, that is H_{0i} is evaluated by the statistic Z_i, such that if $Z_i <= c$, the test is positive, whereas if $Z_i > c$, the test is negative, where *c* denotes the critical value for all *m* tests. Here Z_i could represent a p-value or other statistic for which we may not know the distribution. Let *S* denote the number of positive tests (discoveries) after conducting all *m* tests at threshold *c*. A permutation-based FDR can be estimated if the assumption of exchangeability under the null can be satisfied[1]. This strategy requires generating realizations of Z_i under the null via permutation. For example, if Z_i represents a p-value to evaluate evidence of association between two random variables, then a permutation strategy would be to randomly permute one of the two variables with respect to the other. Let S^* denote the count of positive tests under the condition that all *m* tests are conducted using permuted data. Consider multiple sets of permuted results, where all *m* tests are conducted a total of *B* times, yielding *B* positive test counts, $\bar{S}_j$, $j = 1,\ldots,B$.

Millstein and Volfson[2] (MV) showed that an FDR estimate can be computed from counts of positive tests in the observed (non-permuted) and permuted data,

$$\widehat{FDR} = \frac{\bar{S}^*}{S}\frac{1{-}S/m}{1-\bar{S}^*/m} \tag{1}$$

where $\bar{S}^*$ is the mean count of discoveries in the permuted results. They also showed that this estimator is less conservative than the Benjamini and Hochberg (BH) approach[3] but more conservative than the Storey and Tibshirani approach[4]. Here FDR is defined only if there is a non-zero number of positive tests in the observed data. Millstein and Volfson demonstrated that MV FDR can be interpreted as an odds ratio between the observed and permuted test results, that is, the odds of a positive test in the permuted divided by the odds in the observed. It follows that the variance of the log of FDR is as follows,

$$\hat{\sigma}^2_{\log(FDR)} = \frac{1}{\Sigma S^*_i} + \frac{1}{mB - \Sigma S^*_i} + \frac{1}{S} + \frac{1}{m{-}S}, \tag{2}$$

thus a confidence interval,

$$CI_{FDR} = \exp\{\log(\widehat{FDR}) \pm z_{\frac{\alpha}{2}}\hat{\sigma}_{\log(FDR)}\}, \tag{3}$$

can be estimated in an approach analogous to that commonly used for the odds ratio[5]. Millstein and Volfson accounted for dependencies across tests with an over-dispersion parameter estimated from the permuted results.

The main contribution of the approach, implemented in the R package 'fdrci', is that it quantifies uncertainty in a non-parametric FDR estimator, allowing FDR to be estimated and the precision of that estimate quantified for a post hoc chosen threshold (critical value). In this way, the strength of evidence for a set of discoveries is evaluated not solely by the magnitude of the FDR estimate but also by its uncertainty. By de-emphasizing the FDR magnitude, this approach de-

emphasizes conventional thresholds for discovery, such as 0.05 or 0.1. However, a remaining challenge is potential bias in the confidence interval bounds caused by post hoc selection of FDR CIs. The problem is two-fold, 1) ignoring multiplicity of intervals causes similar problems as ignoring multiplicity of tests, and 2) selection may result in intervals failing to provide the assumed coverage[6].

FDR-Adjusted Selected CIs – CI, p-value Agreement

One of the intents of Benjamini and Yekutieli was development of CIs that agreed with statistical tests in the sense that a significant test would correspond to a confidence interval that does not cover the null value and vice versa. However, it is possible here for a BY selected interval to cover the null value (typically FDR = 1). The apparent discrepancy involves the one-sided nature of the test of FDR at each threshold. It is one-sided because FDR is theoretically bounded by one and cannot be larger. However, the 95% CI is two-sided, resulting in the possibility that FDR could be significantly smaller than one (one-sided test) even if the 95% CI covers one (two-sided interval). One way of addressing this duality would be to estimate one-sided upper 95% CIs rather than two-sided intervals. This approach also makes sense considering that with FDR, the upper bound is much more important than the lower bound.

Simulation Studies

Computer simulations were conducted to explore the statistical properties of likelihood ratio tests (LRT) of main effects and interactions in the context of Cox proportional hazards regression models. Previous authors have noted in the context of linear models that testing main effects and interactions in multivariable models pose a particular challenge for permutation tests due to dependences that could exist between covariates under the null hypothesis[7,8].

For example, consider the following Cox models described in Methods,

Model 2:
$$\ln\left(\frac{h(t)}{h_0(t)}\right) = \Sigma\gamma_i W_i + \beta T + \delta G$$

Model 3:
$$\ln\left(\frac{h(t)}{h_0(t)}\right) = \Sigma\gamma_i W_i + \beta T + \delta G + \omega TG$$

where $h(t)$ denotes the hazard function for time t, W denotes the vector of adjustment covariates, T denotes an indicator variable for treatment, and G denotes the imputed gene expression. In Model 2 we are interested in testing the null hypothesis that $\delta = 0$, however, dependencies may exist between G and elements of W, which themselves may have non-zero effects, even if the null is true. Thus, randomly permuting G would break any such dependencies. The implication is that the distribution of the test statistic may not exactly capture the null distribution. The challenge for a permutation test for ω in Model 3 is similar. In addition to the problems just described, permuting G would force $\delta = 0$, which may not be true under the null.

It is important to note that in the analysis of the MAVERICC data in this report, the purpose of using permutations to approximate the null distribution is not primarily to correct for unsatisfied parametric assumptions but rather to estimate 95% CIs for FDR estimates. Permutations are required in the MV FDR approach for computing 95% CIs that account for dependencies across tests[2]. In this case, the parametric assumptions are likely to be approximately satisfied and the null distributions of LRT statistics for δ and ω are expected to be independent of the other parameters under the null[9].

Computer simulations were performed to determine how well permuting *G* captures the null distribution for LRTs of δ (Model 2) and ω (Model 3). The sample size for each simulated dataset was 329 to represent the MAVERICC clinical trial. A total of 3 covariates were simulated, including one adjustment covariate, *X*, one treatment indicator, *T*, and one gene, *G*. *X* and *G* were simulated to be Gausian correlated variables (r approx. 0.6) and T was independently simulated as 1:1 control:treated. Two sets of simulations were conducted for positive (Table S1) and negative (Table S2) correlations between *X* and *G*.

Survival times and events were simulated using the R package, "simsurv", according to an exponential distribution with a baseline hazard of 0.2 and time truncated at 10. Three sets of simulations were conducted, A) without outliers or high leverage values, B) with outliers and high leverage values, and C) with outliers and high leverage values but then discretizing *G* into an ordinal variable according to its tertiles. Within each simulation set, three parameter scenarios were simulated, a) $\delta = 0$ and $\omega = 0$, b) $\delta = 0.2$ and $\omega = 0$, and c) $\delta = 0.2$ and $\omega = 0.2$. The coefficients for *X* and *T* were set to 0.2 in all scenarios. Data were simulated with and without outliers in survival time and high leverage values in *G*. Five outliers were generated by randomly selecting 5 observations and multiplying the survival times by 5. High leverage values in *G* were generated by randomly selecting 5 observations and adding 5 standard deviations to those values. For each parameter scenario within each simulation set, 2,000 replicate dataset simulations were conducted. For each simulated dataset, Cox regression and corresponding LRTs were conducted using the raw data and following random permutation of *G*.

For the parametric tests, type I error and statistical power were defined as the proportion of datasets with the LRT p-value < 0.05. For the permutation-based tests, the distribution of p-values from the permutation results were used to form an empirical null distribution, and type I error or statistical power was defined as the proportion of p-values from the non-permutation results with the LRT p-value < the 0.05 percentile of the empirical null distribution.

Table S1. Type I error and statistical power for main effects and interactions via multivariable Cox proportional hazards regression with positive correlations between the gene and an adjustment covariate.

N = 167		Type I Error/Power					
True Value		Parametric		Permutation-based			
β_G	β_{GT}	β_G	β_{GT}	β_G	β_{GT}	Outliers	G Transform
0	0	0.046	0.052	0.049	0.047		
0.2	0	0.988	0.055	0.986	0.050		
0.2	0.2	1.000	0.780	1.000	0.760		
0	0	0.048	0.046	0.051	0.039	√	
0.2	0	0.818	0.111	0.828	0.093	√	
0.2	0.2	0.967	0.420	0.973	0.389	√	
0	0	0.036	0.052	0.049	0.045	√	√
0.2	0	0.896	0.051	0.914	0.047	√	√
0.2	0.2	0.998	0.566	0.998	0.518	√	√

Check marks in the Outliers column indicate high leverage points were included in *G* and outliers in survival time.
G Transform indicates that *G* was modeled as an ordinal variable with values {1,2,3} corresponding to the three tertiles.

Table S2. Type I error and statistical power for main effects and interactions via multivariable Cox proportional hazards regression with negative correlations between the gene and an adjustment covariate.

N = 167		Type I Error/Power					
True Value		Parametric		Permutation-based			
β_G	β_{GT}	β_G	β_{GT}	β_G	β_{GT}	Outliers	G Transform
0	0	0.054	0.047	0.054	0.046		
0.2	0	0.990	0.050	0.990	0.048		
0.2	0.2	1.000	0.781	1.000	0.762		
0	0	0.058	0.045	0.050	0.039	√	
0.2	0	0.822	0.130	0.813	0.120	√	
0.2	0.2	0.966	0.421	0.964	0.406	√	
0	0	0.056	0.050	0.050	0.044	√	√
0.2	0	0.908	0.046	0.900	0.046	√	√
0.2	0.2	0.998	0.580	0.998	0.570	√	√

Check marks in the Outliers column indicate high leverage points were included in *G* and outliers in survival time.
G Transform indicates that *G* was modeled as an ordinal variable with values {1,2,3} corresponding to the three tertiles.

These simulation results indicate that type I error is more tightly controlled with the proposed permutation approach than standard parametric tests. The results also indicate that differences in power are small and primarily attributable to the differences in type I error between the approaches. Interestingly, the only substantial departure from the assumed type I error was when two conditions simultaneously occurred, 1) non-zero main effect of *G*, and 2) high leverage values in *G*. When *G* was transformed into an ordinal variable, this problem was adequately addressed for both parametric and permutation-based approaches. In the simulations generated here, type I error for the permutation-based approach was close to the assumed 0.05 level for the three scenarios where *G* was statistically independent of survival ($\delta = 0$) even with the challenges of correlated covariates, outliers, high leverage points and tests of *T*G* interaction. Thus, these results support the permutation-based approach as a way to identify genes involved in interactions, even without categorization to eliminate high leverage values.

Code example for fdrci package

This code simulates data to demonstrate the selection and adjustment of a series of permutation-based FDR CI's.

```
#############################################################
# Purpose: Simulate observed and permuted results and apply
#          MV FDR approach with BY adjustment to account for
#          multiplicity in FDR interval estimation.

library(fdrci)

n.obs = 100
n.Halternative = 30
n.Hnull = 280
```

```
b = 0.2
n.tests = n.Halternative + n.Hnull
X = matrix(rnorm(n.obs*n.tests),nrow=n.obs,ncol=n.tests)
e.a = matrix(rnorm(n.obs),nrow=n.obs)
beta = matrix(rep(b, n.Halternative), nrow=n.Halternative)

# Simulate outcome Y dependent on the some of the X's
Y = X[,1:n.Halternative]%*%beta + e.a
n.perm = 50

# function to test correlations between all X,Y pairs
myanalysis = function(X,Y){
  ntests = ncol(X)
  rslts = as.data.frame(matrix(NA,nrow=ntests,ncol=2))
  names(rslts) = c("ID","pvalue")
  rslts[,"ID"] = 1:ntests
  for(i in 1:ntests){
    fit = cor.test(X[,i],Y,na.action="na.exclude",
                   alternative="two.sided",method="pearson")
    rslts[i,"pvalue"] = fit$p.value
  }
  return(rslts)
} # End myanalysis

## Generate observed results
obs = myanalysis(X,Y)

## Generate permuted results
perml = vector('list',n.perm)
for(perm in 1:n.perm){
  X1 = X[order(runif(n.obs)),]
  perml[[perm]] = myanalysis(X1,Y)
}

## FDR results tables and plots
lower.bnd = 1
upper.bnd = 4
plotdat =
fdrTbl(obs$pvalue,perml,"pvalue",n.tests,lower.bnd,upper.bnd)

# Plot of FDR, unadjusted CIs as shaded region, and number of
discoveries at each discovery threshold
FDRplot(plotdat, lower.bnd, upper.bnd) # unadjusted CIs

plotdat1 =
fdrTbl(obs$pvalue,perml,"pvalue",n.tests,lower.bnd,upper.bnd,correct="
BH")

# Plot of FDR, BY-adjusted CIs as shaded region, and number of
discoveries at each discovery threshold
# If no region is shaded, no interval was selected
FDRplot(plotdat1, lower.bnd, upper.bnd) # adjusted CIs
```

References

1. Anderson, M., and Braak, C.T. (2003). Permutation tests for multi-factorial analysis of variance. Journal of statistical computation and simulation *72*, 85-113.
2. Millstein, J., and Volfson, D. (2013). Computationally efficient permutation-based confidence interval estimation for tail-area FDR. Front Genet *4*, 179. 10.3389/fgene.2013.00179.
3. Benjamini, Y., and Hochberg, Y. (1995). Controlling the False Discovery Rate: A Practical and Powerful Approach to Multiple Testing. Journal of the Royal statistical society: series B (Methodological) *57.1*, 289-300.
4. Storey, J.D., and Tibshirani, R. (2003). Statistical significance for genomewide studies. Proc Natl Acad Sci U S A *100*, 9440-9445. 10.1073/pnas.1530509100.
5. WOOLF, B. (1955). On estimating the relation between blood group and disease. Ann Hum Genet *19*, 251-253. 10.1111/j.1469-1809.1955.tb01348.x.
6. Benjamini, Y., and Yekutieli, D. (2005). False Discovery Rate–Adjusted Multiple Confidence Intervals for Selected Parameters Journal of the American Statistical Association *100*, 71-81.
7. Berrett, T.B. (2020). The conditional permutation test for independence while controlling for confounders J. R. Statist. Soc. B *82* (*1*), 175–197.
8. Bůžková, P., Lumley, T., and Rice, K. (2011). Permutation and parametric bootstrap tests for gene-gene and gene-environment interactions. Ann Hum Genet *75*, 36-45. 10.1111/j.1469-1809.2010.00572.x.
9. Dai, J.Y., Kooperberg, C., Leblanc, M., and Prentice, R.L. (2012). Two-stage testing procedures with independent filtering for genome-wide gene-environment interaction. Biometrika *99*, 929-944. 10.1093/biomet/ass044.
