# Supplementary material for: fdrci: FDR confidence interval selection and adjustment for large-scale hypothesis testing
Source: Bioinform Adv. 2022 Jun 13;2(1):vbac047. doi: 10.1093/bioadv/vbac047 (PMC9210923; doi:10.1093/bioadv/vbac047)
Supplement: vbac047_Supplementary_Data [file vbac047_supplementary_data.zip › RMarkdown.zip!/BY_FDR_CI_selection.pdf]

fdrci: FDR confidence interval selection and adjustment

Joshua Millstein

5/30/2022

Introduction

This document includes code used in our FDR CI selection paper (Millstein et al. 2022). Through simlulation, the code evaluates an approach for post hoc selection and adjustment of FDR confidence intervals (CIs), accounting for multiplicity. It is then applied to tumor gene expression data from the MAVERICC clinical trial. Essentially, the investigator chooses a series of possible discovery thresholds, and at each threshold FDR is computed along with a p-value that tests the null hypothesis against the alternative that FDR is significantly less than one. The Benjamini and Yekutieli (BY) approach (Benjamini and Yekutieli 2005) via the Benjamini and Hochberg approach (Benjamini and Hochberg 1995) is used to select thresholds corresponding to rejected null hypotheses as described above and to adjust selected FDR CIs for multiplicity. Thus, the investigator is free to choose post hoc from any one (or multiple) of the selected discovery thresholds while maintaining adequate control of FDR CI coverage.

```
# download and install fdrci from github or CRAN
# https://uscbiostats.github.io/fdrci
# library(devtools)
# install_github("USCbiostats/fdrci")
library(fdrci)
library(MASS) # mvrnorm() to simulate mutlivariate normal data
library(ggplot2)
library(gridExtra)
library(dplyr)
```

```
Attaching package: 'dplyr'

The following object is masked from 'package:gridExtra':

    combine

The following object is masked from 'package:MASS':

    select

The following objects are masked from 'package:stats':

    filter, lag

The following objects are masked from 'package:base':

    intersect, setdiff, setequal, union
```

```
library(simsurv)
library(survival)
library(survminer)
```

```
Loading required package: ggpubr

Loading required package: magrittr
```

```
set.seed(052422)
```

Simulations to evaluate the proposed BY selection approach

In genomic studies involving large-scale multiple testing, the investigator is typically faced with the challenge of determining a discovery threshold to identify those results that warrant follow-up research. A common approach is simply choosing an FDR cutoff of 0.05, which follows the typical approach for controlling type I error. More lenient cutoffs such as 0.1 or 0.2 are also used. If FDR CIs are calculated, then another approach could be to require that the upper CI bound be less than a predetermined value.

To evaluate coverage (false coverage statement rate) of BY selected CIs in simulated data, we compared to two other methods along the lines described above under several sets of conditions. The first comparison method (UCB) selects all intervals where the upper bound is less than one. The second comparison method (M.2) selects all intervals where the FDR point estimate is less than 0.2.

The main FDR workhorse function is fdrTbl().

```
# fdrTbl() in the fdrci package, computes FDR and confidence intervals for a series of
# user-specified thresholds. In the new version, shown here as fdrTbl1, there is an option
# to account for multiplicity by using correct="BH" (correct = {"none", "BH"}).
fdrTbl1 = function (obs_vec, perm_list, pname, ntests, lowerbound, upperbound,
          incr = 0.1, cl = 0.95, c1 = NA, correct = "none") {
  plotdat = as.data.frame(matrix(NA, nrow = 0, ncol = 8))
  names(plotdat) = c("threshold", "fdr", "ll", "ul", "pi0",
                     "odp", "S", "Sp")
  thres_ = seq(lowerbound, upperbound, incr)
  for (i in 1:length(thres_)) {
    plotdat[i, "threshold"] = thres_[i]
    thr = 10^-(thres_[i])
    tmp = fdr_od(obs_vec, perm_list, pname, ntests, thr,
                 cl = 0.95, c1)
    if (length(tmp) == length(plotdat) - 1) plotdat[i, -1] = tmp
  }

  if (is.element(correct, "BH")) {
    aa = !is.na(plotdat$fdr)
    bb = !is.na(plotdat$ll)
    cc = !is.na(plotdat$ul)
    indx = which(aa & bb & cc)
    plotdat[!is.element(1:nrow(plotdat),indx), "ll"] = NA
    plotdat[!is.element(1:nrow(plotdat),indx), "ul"] = NA
    if (length(indx) > 1) {
      tmpt = plotdat[indx, c("fdr", "ll", "ul")]
      alpha = 1 - cl
      fdr_se = (log(tmpt$fdr) - log(tmpt$ll))/qnorm(1 - alpha/2)
      z = log(tmpt$fdr)/fdr_se
      p = pnorm(z, lower.tail = TRUE)
      qval = p.adjust(p, method="BH")
      sig = qval <= alpha
      R = sum(sig)
      alpha_a = R * alpha/length(p)
      ll_a = exp(log(tmpt$fdr) - qnorm(1 - alpha_a/2) * fdr_se)
```

```
      ul_a = exp(log(tmpt$fdr) + qnorm(1 - alpha_a/2) * fdr_se)
      ll_a = ifelse(sig, ll_a, NA)
      ul_a = ifelse(sig, ul_a, NA)
      plotdat[indx, "ll"] = ifelse(ll_a <= 1, ll_a, 1)
      plotdat[indx, "ul"] = ifelse(ul_a <= 1, ul_a, 1)
    }
  }
  return(plotdat)
}
```

To simlulate data data that includes dependencies, we call a function that simulates a block of multivariate Gaussian variables.

```
# Input
# n: sample size
# m: number of features
# cv: vector with lower and upper bounds for off diagonal elements of the covariance matrix.
#     In this function the exact covariances can be random if lower < upper.
# b: range for beta values that define dependencies between X and Y variables.

sim.blk = function( n, m, cv, b ){
  posdef = FALSE
  wcount = 1
  while(!posdef){
    if(wcount > 100) break
    s = matrix( runif(m*m, cv[1], cv[2]), ncol=m, nrow=m )
    s[lower.tri(s)] = t(s)[lower.tri(s)]
    diag(s) = 1
    x=try( mvrnorm( n, mu=rep(0,m), Sigma=s ), silent=TRUE)
    posdef = length(x) > 1
    wcount = wcount + 1
  }
  beta = runif( m, b[1], b[2] )
  y = beta %*% t(x) + rnorm( n )

  outp = vector('list', 2)
  outp[[1]] = as.data.frame(x)
  outp[[2]] = as.data.frame(y)
  return( outp )
} # End function sim.blk.m

### Function to simulate block diagonal matrix based on blocks simulated using sim.blk
sim.blk.diag = function( blk.num, blk.size, cv, b, n  ){

  ydat = as.data.frame( matrix( NA, nrow=n, ncol=blk.num ) )
  for( blk in 1:blk.num ){
    m = blk.size
    tmp=sim.blk( n, m, cv, b )
    colnames( tmp[[ 1 ]] ) = paste( "X.h1.", blk, ".", 1:m, ".", sep="" )
    if( blk == 1 ) xdat = tmp[[ 1 ]] else xdat = cbind( xdat, tmp[[ 1 ]] )
    ydat[ , blk ] = as.numeric(tmp[[ 2 ]])
  } # End blk loop

  colnames( ydat ) = paste( "Y.h1", ".", 1:blk.num, ".", sep="" )
```

```
  outp = vector('list', 2)
  outp[[1]] = xdat
  outp[[2]] = ydat
  return( outp )

} # End function sim.blk.diag.m
```

The goal in these simulated studies is to identify associations between a set of molecular factors and a set of response factors using simple pairwise tests of Pearson's correlation coefficient.

```
### Function to test correlations between all pairs of variables between (but not within) two matrices
corfun1.m = function( vec, mat1, mat2 ) cor.test( mat1[ , vec[1]], mat2[ , vec[2]], method="pearson" )$p
corfun.m = function( mat1, mat2 ){
  mat1 = as.data.frame(mat1)
  mat2 = as.data.frame(mat2)
  pmat = as.data.frame( matrix( NA, nrow=(ncol(mat1)*ncol(mat2)), ncol=3 ) )
  names(pmat) = c( "col.mat1", "col.mat2", "p.value" )
  pmat[, "col.mat1" ] = rep( colnames(mat1), each=ncol(mat2) )
  pmat[, "col.mat2" ] = rep( colnames(mat2), ncol(mat1) )
  pmat[, "p.value" ] = apply( pmat[,1:2], 1, corfun1.m, mat1=mat1, mat2=mat2 )
  return( pmat )
} # End function corfun.m
```

For the main simulation function, we create dependencies between a relatively small set of predictor-response pairs while all remaining pairs are statistically independent. FDR and 95% CIs are then computed using fdrTbl1() in a permutation-based approach, with and without adjustment for multiplicity.

```
# Function to simulate block diagonal variables dependent on other variables and analyze them
sim.fdrci = function( cv, b, n, blk.size, blk.num, blk.h1, n.perm ){

  # blocks H1
  tmp = sim.blk.diag( blk.h1, blk.size, cv, b, n  )
  x.tmp = tmp[[1]]
  y.tmp = tmp[[2]]

  # noise variables
  blk.num.noise = blk.num - blk.h1
  if(blk.num.noise > 0){
    tmp1 = sim.blk.diag( blk.num.noise, blk.size, cv, c(0, 0), n  )
    x.n.tmp = tmp1[[1]]
    y.n.tmp = tmp1[[2]]
    names(x.n.tmp) = paste( "X.n", 1:ncol(x.n.tmp), sep="." )
    names(y.n.tmp) = paste( "Y.n", 1:ncol(y.n.tmp), sep="." )
  }

  x.nms.h1 = names(x.tmp)
  y.nms.h1 = names(y.tmp)

  if(blk.num.noise > 0){
    x = cbind( x.tmp, x.n.tmp )
    y = cbind( y.tmp, y.n.tmp )
  } else {
    x = x.tmp
    y = y.tmp
```

```
}
rslts.y = corfun.m( x, y )

for( blk in 1:blk.h1 ){
  tag = paste( "h1.", blk, ".", sep="" )
  ind.y.x = grep( tag, rslts.y[,"col.mat1"], fixed=TRUE, value=FALSE )
  ind.y = grep( tag, rslts.y[,"col.mat2"], fixed=TRUE, value=FALSE )
  ind = ind.y[ is.element( ind.y, ind.y.x ) ]
  if(blk == 1) ind.h1 = ind
  if(blk > 1) ind.h1 = c( ind.h1, ind )
} # end blk loop

rslts.y[, "H"] = 0
rslts.y[ind.h1, "H"] = 1
obs = rslts.y

perml = vector('list',n.perm)
for(perm in 1:n.perm){
  y.perm = y[sample(nrow(y)), ]
  rslts.y = corfun.m( x, y.perm )

  for( blk in 1:blk.num ){
    tag = paste( "h1.", blk, ".", sep="" )
    ind.y.x = grep( tag, rslts.y[,"col.mat1"], fixed=TRUE, value=FALSE )
    ind.y = grep( tag, rslts.y[,"col.mat2"], fixed=TRUE, value=FALSE )
    ind = ind.y[ is.element( ind.y, ind.y.x ) ]
    if(blk == 1) ind.h1 = ind
    if(blk > 1) ind.h1 = c( ind.h1, ind )
  } # end blk loop

  rslts.y[, "H"] = 0
  rslts.y[ind.h1, "H"] = 1
  perml[[perm]] = rslts.y
}

## Create table of results for a series of significance thresholds
mytbl = fdrTbl1(obs$p.value,perml,"p.value",nrow(obs),2,6)
mytbl
mytbl[,"thresh"] = 10^-(mytbl[,"threshold"])
mytbl[,"fdr.true"] = NA
mytbl = mytbl[!is.na(mytbl[,"ul"]), ]

# add true fdr to table and coverage
if(nrow(mytbl) > 0){
  for(i in 1:nrow(mytbl)){
    aa = obs[,"p.value"] <= mytbl[i, "thresh"]
    n.discoveries = sum(aa & obs[, "H"])
    mytbl[i,"fdr.true"] = (mytbl[i,"S"] - n.discoveries) / mytbl[i,"S"]
  }
}
mytbl.ua = mytbl

## Create table of results for a series of significance thresholds
```

```
  mytbl = fdrTbl1(obs$p.value,perml,"p.value",nrow(obs),2,6,correct="BH")
  mytbl[,"thresh"] = 10^-(mytbl[,"threshold"])
  mytbl[,"fdr.true"] = NA
  mytbl = mytbl[!is.na(mytbl[,"ul"]), ]

  # add true fdr to table and coverage
  if(nrow(mytbl) > 0){
    for(i in 1:nrow(mytbl)){
      aa = obs[,"p.value"] <= mytbl[i, "thresh"]
      n.discoveries = sum(aa & obs[, "H"])
      mytbl[i,"fdr.true"] = (mytbl[i,"S"] - n.discoveries) / mytbl[i,"S"]
    }
  }
  mytbl.a = mytbl

  outp = vector('list', 2)
  outp[[1]] = mytbl.ua
  outp[[2]] = mytbl.a

  return( outp )
} # End function sim.fdrci
```

For each simulation scenario, we generate 2000 replicate datasets. Each dataset includes 200 observations. The number of correlated blocks among predictors is 20 and the number of features per block is 5, yielding 100 preditors. There is a single response feature for each block, thus 20 response features per dataset. Because we are interested in finding all associations between predictors and responses, we conduct a total of 100 * 20 = 2000 tests. (The investigator does not know the underlying structure of dependencies so must test all pairs.) This strategy is non-parametric permutation-based approach, so multiple sets of results must be generated under the null by random permutation of some aspect of the data. Here, we randomly permute the response to enforce statistical independence between the predictors and responses. The process is repeated 20 times, to generate 20 sets of results under the null.

Scenario A

A small proportion of predictor-response associations combined with weak effects. Five blocks of predictors are associated with one response (total of 5 response features), yielding 25 of 2000 tests where the alternative hypothesis is true.

```
## A. n.h1 = 25, weak effects beta = 0.05
reps = 2000
fdr.lst = vector('list', reps)
fdr.a.lst = vector('list', reps)
cv = c( .3, .3)
b = c(0.05, 0.05)
n = 200
blk.h1 = 5
blk.size = 5
blk.num = 20
n.perm = 20
select.ul = 1

t1 = Sys.time()
rowno = 1
for( iter in 1:reps ){
```

```
  tmp = sim.fdrci(cv, b, n, blk.size, blk.num, blk.h1, n.perm)
  fdr.lst[[iter]] = tmp[[1]]
  fdr.a.lst[[iter]] = tmp[[2]]
  t2 = Sys.time()
  print( paste( t1, "; ", t2, "Rep:", iter ) )
  rowno = rowno + 1
} # End rep loop

# compute E[FDR]
fdr.e = fdr.lst[[1]][, c("threshold", "fdr.true")]
fdr.a.e = fdr.a.lst[[1]][, c("threshold", "fdr.true")]

fl = "rslts_A_estimate_v10.Rdata"
save(fdr.lst, fdr.a.lst, file=fl)
```

Scenario B

A larger proportion of predictor-response associations combined with weak effects. Ten blocks of predictors are associated with one response (total of 10 response features), yielding 50 of 2000 tests where the alternative hypothesis is true.

```
## B. n.h1 = 50, weak effects beta = 0.05
reps = 2000
fdr.lst = vector('list', reps)
fdr.a.lst = vector('list', reps)
cv = c( .3, .3)
b = c(0.05, 0.05)
n = 200
blk.h1 = 10
blk.size = 5
blk.num = 20
n.perm = 20

t1 = Sys.time()
rowno = 1
for( iter in 1:reps ){
  tmp = sim.fdrci(cv, b, n, blk.size, blk.num, blk.h1, n.perm)
  fdr.lst[[iter]] = tmp[[1]]
  fdr.a.lst[[iter]] = tmp[[2]]
  t2 = Sys.time()
  print( paste( t1, "; ", t2, "Rep:", iter ) )
  rowno = rowno + 1
} # End rep loop

# compute E[FDR]
fdr.e = fdr.lst[[1]][, c("threshold", "fdr.true")]
fdr.a.e = fdr.a.lst[[1]][, c("threshold", "fdr.true")]

fl = "rslts_B_estimate_v10.Rdata"
save(fdr.lst, fdr.a.lst, file=fl)
```

Scenario C

A small proportion of predictor-response associations combined with medium size effects. Five blocks of predictors are associated with one response (total of 5 response features), yielding 25 of 2000 tests where the alternative hypothesis is true.

```
## C. n.h1 = 25, medium effects beta = 0.075
reps = 2000
fdr.lst = vector('list', reps)
fdr.a.lst = vector('list', reps)
cv = c( .3, .3)
b = c(0.075, 0.075)
n = 200
blk.h1 = 5
blk.size = 5
blk.num = 20
n.perm = 20

t1 = Sys.time()
rowno = 1
for( iter in 1:reps ){
  tmp = sim.fdrci(cv, b, n, blk.size, blk.num, blk.h1, n.perm)
  fdr.lst[[iter]] = tmp[[1]]
  fdr.a.lst[[iter]] = tmp[[2]]
  t2 = Sys.time()
  print( paste( t1, "; ", t2, "Rep:", iter ) )
  rowno = rowno + 1
} # End rep loop

# compute E[FDR]
fdr.e = fdr.lst[[1]][, c("threshold", "fdr.true")]
fdr.a.e = fdr.a.lst[[1]][, c("threshold", "fdr.true")]

fl = "rslts_C_estimate_v10.Rdata"
save(fdr.lst, fdr.a.lst, file=fl)
```

Scenario D

A larger proportion of predictor-response associations combined with medium size effects. Ten blocks of predictors are associated with one response (total of 10 response features), yielding 50 of 2000 tests where the alternative hypothesis is true.

```
## D. n.h1 = 50, medium effects beta = 0.075
reps = 2000
fdr.lst = vector('list', reps)
fdr.a.lst = vector('list', reps)
cv = c( .3, .3)
b = c(0.075, 0.075)
n = 200
blk.h1 = 10
blk.size = 5
blk.num = 20
n.perm = 20
select.ul = 0.3
```

```
t1 = Sys.time()
rowno = 1
for( iter in 1:reps ){
  tmp = sim.fdrci(cv, b, n, blk.size, blk.num, blk.h1, n.perm)
  fdr.lst[[iter]] = tmp[[1]]
  fdr.a.lst[[iter]] = tmp[[2]]
  t2 = Sys.time()
  print( paste( t1, "; ", t2, "Rep:", iter ) )
  rowno = rowno + 1
} # End rep loop

# compute E[FDR]
fdr.e = fdr.lst[[1]][, c("threshold", "fdr.true")]
fdr.a.e = fdr.a.lst[[1]][, c("threshold", "fdr.true")]

fl = "rslts_D_estimate_v10.Rdata"
save(fdr.lst, fdr.a.lst, file=fl)
```

Scenario E

Here all predictors are independent of all responses, hence this scenario represents the global nell. Here the null hypothesis is true for all 2000 tests.

```
## E. n.h1 = 0, zero effects beta = 0.0
reps = 2000
fdr.lst = vector('list', reps)
fdr.a.lst = vector('list', reps)
cv = c( .3, .3)
b = c(0.0, 0.0)
n = 200
blk.h1 = 1
blk.size = 5
blk.num = 20
n.perm = 20

t1 = Sys.time()
rowno = 1
for( iter in 1:reps ){
  tmp = sim.fdrci(cv, b, n, blk.size, blk.num, blk.h1, n.perm)
  fdr.lst[[iter]] = tmp[[1]]
  fdr.a.lst[[iter]] = tmp[[2]]
  t2 = Sys.time()
  print( paste( t1, "; ", t2, "Rep:", iter ) )
  rowno = rowno + 1
} # End rep loop

# compute E[FDR]
fdr.e = fdr.lst[[1]][, c("threshold", "fdr.true")]
fdr.a.e = fdr.a.lst[[1]][, c("threshold", "fdr.true")]

fl = "rslts_E_estimate_v10.Rdata"
save(fdr.lst, fdr.a.lst, file=fl)
```

CI coverage estimation

The false coverage statement rate (FCR) is defined as the expected proportion of parameters not covered by their intervals among the selected parameters and where the proportion is defined to be zero if no parameters are selected. Our measure of coverage is 1 - FCR. We will estimate coverage for each simulation scenario above for the three approaches (USB, M.2, and BY). We don't know the true FDR exactly for each discovery threshold, but we do know the true and false hypotheses, so using the 2000 datasets, we can get very close.

```
# Total number of tests:
blk.size = 5 # number of features per block
blk.num = 20 # total number of blocks and number of outcomes
n.tests = blk.num * blk.size * blk.num # number of x-features * number of y-features

# 1. estimate E[FDR], stratified by scenario and S
scen = LETTERS[1:5]
fdr.expect.ci = vector('list', 5)
names(fdr.expect.ci) = scen
fdr.expect.2 = vector('list', 5)
names(fdr.expect.2) = scen
fdr.expect.bh = vector('list', 5)
names(fdr.expect.bh) = scen

nms = c("scenario", "rep",
        "coverage.ci", "coverage.ll.ci", "coverage.ul.ci",
        "coverage.2", "coverage.ll.2", "coverage.ul.2",
        "coverage.bh", "coverage.ll.bh", "coverage.ul.bh")
myrslts = as.data.frame(matrix(NA, nrow=0, ncol=length(nms)))
names(myrslts) = nms
rowno.r = 1

for(s.ind in 1:length(scen)){
  s = scen[s.ind]
  fl = paste("rslts_", s,"_estimate_v10.Rdata", sep="")
  load(file=fl)
  fdr.lst.tmp = fdr.lst
  fdr.a.lst.tmp = fdr.a.lst

  # compute E[FDR]
  fdr.e.ci = fdr.lst[[1]][, c("threshold", "fdr.true")]
  fdr.e.2 = fdr.lst[[1]][, c("threshold", "fdr.true")]
  fdr.e.bh = fdr.a.lst[[1]][, c("threshold", "fdr.true")]
  thrsh = seq(2, 6, .1)
  fdr.e.ci[1:length(thrsh),"threshold"] = thrsh
  fdr.e.2[1:length(thrsh),"threshold"] = thrsh
  fdr.e.bh[1:length(thrsh),"threshold"] = thrsh
  fdr.e.ci[,"fdr.true"] = 0
  fdr.e.2[,"fdr.true"] = 0
  fdr.e.bh[,"fdr.true"] = 0
  fdr.e.ci[,"fdr.sum"] = 0
  fdr.e.2[,"fdr.sum"] = 0
  fdr.e.bh[,"fdr.sum"] = 0
  fdr.e.ci[,"fdr.count"] = 0
  fdr.e.2[,"fdr.count"] = 0
  fdr.e.bh[,"fdr.count"] = 0
```

```
for(i in 1:length(fdr.lst)){
  tmp = fdr.lst[[i]]
  tmp.ci = tmp[tmp[, "ul"] < 1, ] # select FDR by ul < 1
  tmp.2 = tmp[tmp[, "fdr"] < .2, ] # select FDR by fdr < 0.2
  tmp.bh = fdr.a.lst[[i]]
  flag = FALSE
  if(nrow(tmp.ci) > 0){
    for(j in 1:nrow(tmp.ci)){
      rowno = match(tmp.ci[ j,"threshold"], fdr.e.ci[,"threshold"])
      fdr.e.ci[ rowno,"fdr.sum"] = fdr.e.ci[ rowno,"fdr.sum"] + tmp.ci[ j,"fdr.true"]
      fdr.e.ci[ rowno,"fdr.count"] = fdr.e.ci[ rowno,"fdr.count"] + 1
    }
  }
  if(nrow(tmp.2) > 0){
    for(j in 1:nrow(tmp.2)){
      rowno = match(tmp.2[ j,"threshold"], fdr.e.2[,"threshold"])
      fdr.e.2[ rowno,"fdr.sum"] = fdr.e.2[ rowno,"fdr.sum"] + tmp.2[ j,"fdr.true"]
      fdr.e.2[ rowno,"fdr.count"] = fdr.e.2[ rowno,"fdr.count"] + 1
    }
  }
  if(nrow(tmp.bh) > 0){
    for(j in 1:nrow(tmp.bh)){
      rowno = match(tmp.bh[ j,"threshold"], fdr.e.bh[,"threshold"])
      fdr.e.bh[ rowno,"fdr.sum"] = fdr.e.bh[ rowno,"fdr.sum"] + tmp.bh[ j,"fdr.true"]
      fdr.e.bh[ rowno,"fdr.count"] = fdr.e.bh[ rowno,"fdr.count"] + 1
    }
  }
} # end i loop
fdr.e.ci[,"fdr.true"] = fdr.e.ci[,"fdr.sum"] / fdr.e.ci[,"fdr.count"]
fdr.e.2[,"fdr.true"] = fdr.e.2[,"fdr.sum"] / fdr.e.2[,"fdr.count"]
fdr.e.bh[,"fdr.true"] = fdr.e.bh[,"fdr.sum"] / fdr.e.bh[,"fdr.count"]
if(is.element(s, "E")){
  # scenario E is simulated under globel null w/ beta = 0
  fdr.e.ci[,"fdr.true"] = 1
  fdr.e.2[,"fdr.true"] = 1
  fdr.e.bh[,"fdr.true"] = 1
}
fdr.expect.ci[[s.ind]] = fdr.e.ci
fdr.expect.2[[s.ind]] = fdr.e.2
fdr.expect.bh[[s.ind]] = fdr.e.bh

# compute coverage based on E[FDR]
for(i in 1:length(fdr.lst)){
  tmp = fdr.lst[[i]]
  tmp[,"coverage"] = NA
  tmp[,"coverage.ll"] = NA
  tmp[,"coverage.ul"] = NA
  tmp.ci = tmp[tmp[, "ul"] < 1, ] # select FDR by ul < 1
  tmp.2 = tmp[tmp[, "fdr"] < .2, ] # select FDR by fdr < 0.2
  tmp.bh = fdr.a.lst[[i]]
  if(nrow(tmp.bh) > 0){
    tmp.bh[,"coverage"] = NA
    tmp.bh[,"coverage.ll"] = NA
```

```
    tmp.bh[,"coverage.ul"] = NA
  }
  if(nrow(tmp.ci) > 0){
    for(j in 1:nrow(tmp.ci)){
      rowno = match(tmp.ci[ j,"threshold"], fdr.e.ci[,"threshold"])
      tmp.ci[ j,"fdr.true"] = fdr.e.ci[ rowno,"fdr.true"]
      tmp.ci[ j,"coverage"] = ifelse((tmp.ci[ j,"fdr.true"] >= tmp.ci[ j,"ll"]) & (tmp.ci[ j,"fdr.tru
      tmp.ci[ j,"coverage.ll"] = ifelse(tmp.ci[ j,"fdr.true"] >= tmp.ci[ j,"ll"], 1, 0)
      tmp.ci[ j,"coverage.ul"] = ifelse(tmp.ci[ j,"fdr.true"] <= tmp.ci[ j,"ul"], 1, 0)
    }
  } # end if nrow(tmp.ci) > 0
  if(nrow(tmp.2) > 0){
    for(j in 1:nrow(tmp.2)){
      rowno = match(tmp.2[ j,"threshold"], fdr.e.2[,"threshold"])
      tmp.2[ j,"fdr.true"] = fdr.e.2[ rowno,"fdr.true"]
      tmp.2[ j,"coverage"] = ifelse((tmp.2[ j,"fdr.true"] >= tmp.2[ j,"ll"]) & (tmp.2[ j,"fdr.true"]
      tmp.2[ j,"coverage.ll"] = ifelse(tmp.2[ j,"fdr.true"] >= tmp.2[ j,"ll"], 1, 0)
      tmp.2[ j,"coverage.ul"] = ifelse(tmp.2[ j,"fdr.true"] <= tmp.2[ j,"ul"], 1, 0)
    }
  } # end if nrow(tmp.2) > 0
  if(nrow(tmp.bh) > 0){
    for(j in 1:nrow(tmp.bh)){
      rowno = match(tmp.bh[ j,"threshold"], fdr.e.bh[,"threshold"])
      tmp.bh[ j,"fdr.true"] = fdr.e.bh[ rowno,"fdr.true"]
      tmp.bh[ j,"coverage"] = ifelse((tmp.bh[ j,"fdr.true"] >= tmp.bh[ j,"ll"]) & (tmp.bh[ j,"fdr.tru
      tmp.bh[ j,"coverage.ll"] = ifelse(tmp.bh[ j,"fdr.true"] >= tmp.bh[ j,"ll"], 1, 0)
      tmp.bh[ j,"coverage.ul"] = ifelse(tmp.bh[ j,"fdr.true"] <= tmp.bh[ j,"ul"], 1, 0)
    }
  } # end if nrow(tmp.bh) > 0

  myrslts[rowno.r, "rep"] = i
  myrslts[rowno.r, "scenario"] = s
  if(nrow(tmp.ci) > 0){
    myrslts[rowno.r, "coverage.ci"] = mean(tmp.ci[,"coverage"])
    myrslts[rowno.r, "coverage.ll.ci"] = mean(tmp.ci[,"coverage.ll"])
    myrslts[rowno.r, "coverage.ul.ci"] = mean(tmp.ci[,"coverage.ul"])
  } else {
    myrslts[rowno.r, "coverage.ci"] = 1
    myrslts[rowno.r, "coverage.ll.ci"] = 1
    myrslts[rowno.r, "coverage.ul.ci"] = 1
  }
  if(nrow(tmp.2) > 0){
    myrslts[rowno.r, "coverage.2"] = mean(tmp.2[,"coverage"])
    myrslts[rowno.r, "coverage.ll.2"] = mean(tmp.2[,"coverage.ll"])
    myrslts[rowno.r, "coverage.ul.2"] = mean(tmp.2[,"coverage.ul"])
  } else {
    myrslts[rowno.r, "coverage.2"] = 1
    myrslts[rowno.r, "coverage.ll.2"] = 1
    myrslts[rowno.r, "coverage.ul.2"] = 1
  }
  if(nrow(tmp.bh) > 0){
    myrslts[rowno.r, "coverage.bh"] = mean(tmp.bh[,"coverage"])
    myrslts[rowno.r, "coverage.ll.bh"] = mean(tmp.bh[,"coverage.ll"])
```

```
      myrslts[rowno.r, "coverage.ul.bh"] = mean(tmp.bh[,"coverage.ul"])
    } else {
      myrslts[rowno.r, "coverage.bh"] = 1
      myrslts[rowno.r, "coverage.ll.bh"] = 1
      myrslts[rowno.r, "coverage.ul.bh"] = 1
    }
    rowno.r = rowno.r + 1
  }
} # end scen loop

tab1 <- myrslts %>% group_by(scenario) %>% summarise(
  UCB = mean(coverage.ci),
  M.2 = mean(coverage.2),
  BY = mean(coverage.bh)
)
knitr::kable(tab1, caption = "Coverage (1 - FCR) for three CI selection strategies",
             digits=3)
```

Table 1: Coverage (1 - FCR) for three CI selection strategies

scenario	UCB	M.2	BY
A	0.942	0.954	0.973
B	0.965	0.969	0.979
C	0.971	0.972	0.976
D	0.973	0.978	0.976
E	0.780	0.935	0.969

Conditional coverage estimation

It's also of interest for us to look at coverage among only those intervals that were selected, conditional coverage.

```
##### Conditional coverage:
# group E[FDR] into 0-0.05, 0.05-0.1, 0.1-0.2, 0.2-0.4, 0.4-0.6, 0.6-1
# group S: 1-5, 5-10, 10-20, 20+
# 1. estimate E[FDR], stratified by scenario and S
scen = LETTERS[1:5]
fdr.expect.ci = vector('list', 5)
names(fdr.expect.ci) = scen
fdr.expect.2 = vector('list', 5)
names(fdr.expect.2) = scen
fdr.expect.bh = vector('list', 5)
names(fdr.expect.bh) = scen

myrslts.ci = NA
myrslts.2 = NA
myrslts.bh = NA

for(s.ind in 1:length(scen)){
  s = scen[s.ind]
  fl = paste("rslts_", s,"_estimate_v10.Rdata", sep="")
  load(file=fl)
```

```
fdr.lst.tmp = fdr.lst
fdr.a.lst.tmp = fdr.a.lst

# compute E[FDR]
fdr.e.ci = fdr.lst[[1]][, c("threshold", "fdr.true")]
fdr.e.2 = fdr.lst[[1]][, c("threshold", "fdr.true")]
fdr.e.bh = fdr.a.lst[[1]][, c("threshold", "fdr.true")]
thrsh = seq(2, 6, .1)
fdr.e.ci[1:length(thrsh),"threshold"] = thrsh
fdr.e.2[1:length(thrsh),"threshold"] = thrsh
fdr.e.bh[1:length(thrsh),"threshold"] = thrsh
fdr.e.ci[,"fdr.true"] = 0
fdr.e.2[,"fdr.true"] = 0
fdr.e.bh[,"fdr.true"] = 0
fdr.e.ci[,"fdr.sum"] = 0
fdr.e.2[,"fdr.sum"] = 0
fdr.e.bh[,"fdr.sum"] = 0
fdr.e.ci[,"fdr.count"] = 0
fdr.e.2[,"fdr.count"] = 0
fdr.e.bh[,"fdr.count"] = 0

for(i in 1:length(fdr.lst)){
  tmp = fdr.lst[[i]]
  tmp.ci = tmp[tmp[, "ul"] < 1, ] # select FDR by ul < 1
  tmp.2 = tmp[tmp[, "fdr"] < .2, ] # select FDR by fdr < 0.2
  tmp.bh = fdr.a.lst[[i]]
  flag = FALSE
  if(nrow(tmp.ci) > 0){
    for(j in 1:nrow(tmp.ci)){
      rowno = match(tmp.ci[ j,"threshold"], fdr.e.ci[,"threshold"])
      fdr.e.ci[ rowno,"fdr.sum"] = fdr.e.ci[ rowno,"fdr.sum"] + tmp.ci[ j,"fdr.true"]
      fdr.e.ci[ rowno,"fdr.count"] = fdr.e.ci[ rowno,"fdr.count"] + 1
    }
  }
  if(nrow(tmp.2) > 0){
    for(j in 1:nrow(tmp.2)){
      rowno = match(tmp.2[ j,"threshold"], fdr.e.2[,"threshold"])
      fdr.e.2[ rowno,"fdr.sum"] = fdr.e.2[ rowno,"fdr.sum"] + tmp.2[ j,"fdr.true"]
      fdr.e.2[ rowno,"fdr.count"] = fdr.e.2[ rowno,"fdr.count"] + 1
    }
  }
  if(nrow(tmp.bh) > 0){
    for(j in 1:nrow(tmp.bh)){
      rowno = match(tmp.bh[ j,"threshold"], fdr.e.bh[,"threshold"])
      fdr.e.bh[ rowno,"fdr.sum"] = fdr.e.bh[ rowno,"fdr.sum"] + tmp.bh[ j,"fdr.true"]
      fdr.e.bh[ rowno,"fdr.count"] = fdr.e.bh[ rowno,"fdr.count"] + 1
    }
  }
} # end i loop
fdr.e.ci[,"fdr.true"] = fdr.e.ci[,"fdr.sum"] / fdr.e.ci[,"fdr.count"]
fdr.e.2[,"fdr.true"] = fdr.e.2[,"fdr.sum"] / fdr.e.2[,"fdr.count"]
fdr.e.bh[,"fdr.true"] = fdr.e.bh[,"fdr.sum"] / fdr.e.bh[,"fdr.count"]
if(is.element(s, "E")){
```

```
  # scenario E is simulated under globel null w/ beta = 0
  fdr.e.ci[,"fdr.true"] = 1
  fdr.e.2[,"fdr.true"] = 1
  fdr.e.bh[,"fdr.true"] = 1
}
fdr.expect.ci[[s.ind]] = fdr.e.ci
fdr.expect.2[[s.ind]] = fdr.e.2
fdr.expect.bh[[s.ind]] = fdr.e.bh

# compute coverage based on E[FDR]
for(i in 1:length(fdr.lst)){
  tmp = fdr.lst[[i]]
  tmp[,"coverage"] = NA
  tmp[,"coverage.ll"] = NA
  tmp[,"coverage.ul"] = NA
  tmp.ci = tmp[tmp[, "ul"] < 1, ] # select FDR by ul < 1
  tmp.2 = tmp[tmp[, "fdr"] < .2, ] # select FDR by fdr < 0.2
  tmp.bh = fdr.a.lst[[i]]
  if(nrow(tmp.bh) > 0){
    tmp.bh[,"coverage"] = NA
    tmp.bh[,"coverage.ll"] = NA
    tmp.bh[,"coverage.ul"] = NA
  }
  if(nrow(tmp.ci) > 0){
    for(j in 1:nrow(tmp.ci)){
      rowno = match(tmp.ci[ j,"threshold"], fdr.e.ci[,"threshold"])
      tmp.ci[ j,"fdr.true"] = fdr.e.ci[ rowno,"fdr.true"]
      tmp.ci[ j,"coverage"] = ifelse((tmp.ci[ j,"fdr.true"] >= tmp.ci[ j,"ll"]) & (tmp.ci[ j,"fdr.tru
      tmp.ci[ j,"coverage.ll"] = ifelse(tmp.ci[ j,"fdr.true"] >= tmp.ci[ j,"ll"], 1, 0)
      tmp.ci[ j,"coverage.ul"] = ifelse(tmp.ci[ j,"fdr.true"] <= tmp.ci[ j,"ul"], 1, 0)
    }
    tmp.ci[, "scenario"] = s
    tmp.ci[, "rep"] = i
    if(length(myrslts.ci) == 1) myrslts.ci = tmp.ci else myrslts.ci = rbind(myrslts.ci, tmp.ci)
  } # end if nrow(tmp.ci) > 0
  if(nrow(tmp.2) > 0){
    for(j in 1:nrow(tmp.2)){
      rowno = match(tmp.2[ j,"threshold"], fdr.e.2[,"threshold"])
      tmp.2[ j,"fdr.true"] = fdr.e.2[ rowno,"fdr.true"]
      tmp.2[ j,"coverage"] = ifelse((tmp.2[ j,"fdr.true"] >= tmp.2[ j,"ll"]) & (tmp.2[ j,"fdr.true"] 
      tmp.2[ j,"coverage.ll"] = ifelse(tmp.2[ j,"fdr.true"] >= tmp.2[ j,"ll"], 1, 0)
      tmp.2[ j,"coverage.ul"] = ifelse(tmp.2[ j,"fdr.true"] <= tmp.2[ j,"ul"], 1, 0)
    }
    tmp.2[, "scenario"] = s
    tmp.2[, "rep"] = i
    if(length(myrslts.2) == 1) myrslts.2 = tmp.2 else myrslts.2 = rbind(myrslts.2, tmp.2)
  } # end if nrow(tmp.2) > 0
  if(nrow(tmp.bh) > 0){
    for(j in 1:nrow(tmp.bh)){
      rowno = match(tmp.bh[ j,"threshold"], fdr.e.2[,"threshold"])
      tmp.bh[ j,"fdr.true"] = fdr.e.bh[ rowno,"fdr.true"]
      tmp.bh[ j,"coverage"] = ifelse((tmp.bh[ j,"fdr.true"] >= tmp.bh[ j,"ll"]) & (tmp.bh[ j,"fdr.tru
      tmp.bh[ j,"coverage.ll"] = ifelse(tmp.bh[ j,"fdr.true"] >= tmp.bh[ j,"ll"], 1, 0)
```

```
        tmp.bh[ j,"coverage.ul"] = ifelse(tmp.bh[ j,"fdr.true"] <= tmp.bh[ j,"ul"], 1, 0)
      }
      tmp.bh[, "scenario"] = s
      tmp.bh[, "rep"] = i
      if(length(myrslts.bh) == 1) myrslts.bh = tmp.bh else myrslts.bh = rbind(myrslts.bh, tmp.bh)
    } # end if nrow(tmp.bh) > 0
  } # end i loop
} # end scen loop

breaks.fdr = c(-.1, 0.1, 0.3, 0.5, 0.99999999)
breaks.S = c(0, 3, 10, 30, 2000)
myrslts.ci[,"fdr.group"] = cut(myrslts.ci[,"fdr.true"], breaks.fdr)
myrslts.ci[,"S.group"] = cut(myrslts.ci[,"S"], breaks.S)
myrslts.ci[,"fdr.S"] = paste(myrslts.ci[,"fdr.group"], myrslts.ci[,"S.group"], sep=":")
myrslts.2[,"fdr.group"] = cut(myrslts.2[,"fdr.true"], breaks.fdr)
myrslts.2[,"S.group"] = cut(myrslts.2[,"S"], breaks.S)
myrslts.2[,"fdr.S"] = paste(myrslts.2[,"fdr.group"], myrslts.2[,"S.group"], sep=":")
myrslts.bh[,"fdr.group"] = cut(myrslts.bh[,"fdr.true"], breaks.fdr)
myrslts.bh[,"S.group"] = cut(myrslts.bh[,"S"], breaks.S)
myrslts.bh[,"fdr.S"] = paste(myrslts.bh[,"fdr.group"], myrslts.bh[,"S.group"], sep=":")

# Selection = CI
l.fdr = levels(myrslts.ci[,"fdr.group"])
l.S = levels(myrslts.ci[,"S.group"])
grps = paste(rep(l.fdr, length(l.S)), rep(l.S, each=length(l.fdr)), sep=":")
nms = c("fdr.group", "S.group", "fdr.S", "reps", "coverage", "coverage.ll", "coverage.ul")
pdat.ci = as.data.frame(matrix(NA, nrow=length(grps), ncol=length(nms)))
names(pdat.ci) = nms
pdat.ci[, "fdr.group"] = rep(l.fdr, length(l.S))
pdat.ci[, "S.group"] = rep(l.S, each=length(l.fdr))
pdat.ci[, "fdr.S"] = grps
pdat.ci[, "reps"] = 0
for(j in 1:nrow(pdat.ci)){
  fdr = pdat.ci[j, "fdr.group"]
  S = pdat.ci[j, "S.group"]
  aa = is.element(myrslts.ci[,"fdr.group"], fdr) & is.element(myrslts.ci[,"S.group"], S)
  tmp = myrslts.ci[aa, ]
  if(nrow(tmp) > 0){
    mycov = aggregate(x = tmp$coverage, by = list(tmp$rep), FUN = mean)
    pdat.ci[j, "reps"] = nrow(mycov)
    pdat.ci[j, "coverage"] = mean(mycov$x)
    mycov.ll = aggregate(x = tmp$coverage.ll, by = list(tmp$rep), FUN = mean)
    pdat.ci[j, "coverage.ll"] = mean(mycov.ll$x)
    mycov.ul = aggregate(x = tmp$coverage.ul, by = list(tmp$rep), FUN = mean)
    pdat.ci[j, "coverage.ul"] = mean(mycov.ul$x)
  }
}
pdat.ci = pdat.ci[pdat.ci$reps > 20, ]

# Selection = 2
l.fdr = levels(myrslts.2[,"fdr.group"])
l.S = levels(myrslts.2[,"S.group"])
grps = paste(rep(l.fdr, length(l.S)), rep(l.S, each=length(l.fdr)), sep=":")
```

```r
nms = c("fdr.group", "S.group", "fdr.S", "reps", "coverage", "coverage.ll", "coverage.ul")
pdat.2 = as.data.frame(matrix(NA, nrow=length(grps), ncol=length(nms)))
names(pdat.2) = nms
pdat.2[, "fdr.group"] = rep(l.fdr, length(l.S))
pdat.2[, "S.group"] = rep(l.S, each=length(l.fdr))
pdat.2[, "fdr.S"] = grps
pdat.2[, "reps"] = 0
for(j in 1:nrow(pdat.2)){
  fdr = pdat.2[j, "fdr.group"]
  S = pdat.2[j, "S.group"]
  aa = is.element(myrslts.2[,"fdr.group"], fdr) & is.element(myrslts.2[,"S.group"], S)
  tmp = myrslts.2[aa, ]
  if(nrow(tmp) > 0){
    mycov = aggregate(x = tmp$coverage, by = list(tmp$rep), FUN = mean)
    pdat.2[j, "reps"] = nrow(mycov)
    pdat.2[j, "coverage"] = mean(mycov$x)
    mycov.ll = aggregate(x = tmp$coverage.ll, by = list(tmp$rep), FUN = mean)
    pdat.2[j, "coverage.ll"] = mean(mycov.ll$x)
    mycov.ul = aggregate(x = tmp$coverage.ul, by = list(tmp$rep), FUN = mean)
    pdat.2[j, "coverage.ul"] = mean(mycov.ul$x)
  }
}
pdat.2 = pdat.2[pdat.2$reps > 20, ]

# Selection = BH
l.fdr = levels(myrslts.bh[,"fdr.group"])
l.S = levels(myrslts.bh[,"S.group"])
grps = paste(rep(l.fdr, length(l.S)), rep(l.S, each=length(l.fdr)), sep=":")
nms = c("fdr.group", "S.group", "fdr.S", "reps", "coverage", "coverage.ll", "coverage.ul")
pdat.bh = as.data.frame(matrix(NA, nrow=length(grps), ncol=length(nms)))
names(pdat.bh) = nms
pdat.bh[, "fdr.group"] = rep(l.fdr, length(l.S))
pdat.bh[, "S.group"] = rep(l.S, each=length(l.fdr))
pdat.bh[, "fdr.S"] = grps
pdat.bh[, "reps"] = 0
for(j in 1:nrow(pdat.bh)){
  fdr = pdat.bh[j, "fdr.group"]
  S = pdat.bh[j, "S.group"]
  aa = is.element(myrslts.bh[,"fdr.group"], fdr) & is.element(myrslts.bh[,"S.group"], S)
  tmp = myrslts.bh[aa, ]
  if(nrow(tmp) > 0){
    mycov = aggregate(x = tmp$coverage, by = list(tmp$rep), FUN = mean)
    pdat.bh[j, "reps"] = nrow(mycov)
    pdat.bh[j, "coverage"] = mean(mycov$x)
    mycov.ll = aggregate(x = tmp$coverage.ll, by = list(tmp$rep), FUN = mean)
    pdat.bh[j, "coverage.ll"] = mean(mycov.ll$x)
    mycov.ul = aggregate(x = tmp$coverage.ul, by = list(tmp$rep), FUN = mean)
    pdat.bh[j, "coverage.ul"] = mean(mycov.ul$x)
  }
}
pdat.bh = pdat.bh[pdat.bh$reps > 20, ]

# by FDR only
```

```
breaks.fdr = c(-.1, 0.05, seq(0.1, 0.9, 0.1), 0.99999999)
myrslts.ci[,"fdr.group"] = cut(myrslts.ci[,"fdr.true"], breaks.fdr)
myrslts.2[,"fdr.group"] = cut(myrslts.2[,"fdr.true"], breaks.fdr)
myrslts.bh[,"fdr.group"] = cut(myrslts.bh[,"fdr.true"], breaks.fdr)

# Selection = CI
l.fdr = levels(myrslts.ci[,"fdr.group"])
nms = c("fdr.group", "reps", "coverage", "coverage.ll", "coverage.ul")
pdat.ci = as.data.frame(matrix(NA, nrow=length(l.fdr), ncol=length(nms)))
names(pdat.ci) = nms
pdat.ci[, "fdr.group"] = l.fdr
pdat.ci[, "reps"] = 0
for(j in 1:nrow(pdat.ci)){
  fdr = pdat.ci[j, "fdr.group"]
  aa = is.element(myrslts.ci[,"fdr.group"], fdr)
  tmp = myrslts.ci[aa, ]
  if(nrow(tmp) > 0){
    mycov = aggregate(x = tmp$coverage, by = list(tmp$rep), FUN = mean)
    pdat.ci[j, "reps"] = nrow(mycov)
    pdat.ci[j, "coverage"] = mean(mycov$x)
    mycov.ll = aggregate(x = tmp$coverage.ll, by = list(tmp$rep), FUN = mean)
    pdat.ci[j, "coverage.ll"] = mean(mycov.ll$x)
    mycov.ul = aggregate(x = tmp$coverage.ul, by = list(tmp$rep), FUN = mean)
    pdat.ci[j, "coverage.ul"] = mean(mycov.ul$x)
  }
}

# Selection = 2
l.fdr = levels(myrslts.2[,"fdr.group"])
nms = c("fdr.group", "reps", "coverage", "coverage.ll", "coverage.ul")
pdat.2 = as.data.frame(matrix(NA, nrow=length(l.fdr), ncol=length(nms)))
names(pdat.2) = nms
pdat.2[, "fdr.group"] = l.fdr
pdat.2[, "reps"] = 0
for(j in 1:nrow(pdat.2)){
  fdr = pdat.2[j, "fdr.group"]
  aa = is.element(myrslts.2[,"fdr.group"], fdr)
  tmp = myrslts.2[aa, ]
  if(nrow(tmp) > 0){
    mycov = aggregate(x = tmp$coverage, by = list(tmp$rep), FUN = mean)
    pdat.2[j, "reps"] = nrow(mycov)
    pdat.2[j, "coverage"] = mean(mycov$x)
    mycov.ll = aggregate(x = tmp$coverage.ll, by = list(tmp$rep), FUN = mean)
    pdat.2[j, "coverage.ll"] = mean(mycov.ll$x)
    mycov.ul = aggregate(x = tmp$coverage.ul, by = list(tmp$rep), FUN = mean)
    pdat.2[j, "coverage.ul"] = mean(mycov.ul$x)
  }
}

# Selection = BH
l.fdr = levels(myrslts.bh[,"fdr.group"])
nms = c("fdr.group", "reps", "coverage", "coverage.ll", "coverage.ul")
pdat.bh = as.data.frame(matrix(NA, nrow=length(l.fdr), ncol=length(nms)))
```

```r
names(pdat.bh) = nms
pdat.bh[, "fdr.group"] = l.fdr
pdat.bh[, "reps"] = 0
for(j in 1:nrow(pdat.bh)){
  fdr = pdat.bh[j, "fdr.group"]
  aa = is.element(myrslts.bh[,"fdr.group"], fdr)
  tmp = myrslts.bh[aa, ]
  if(nrow(tmp) > 0){
    mycov = aggregate(x = tmp$coverage, by = list(tmp$rep), FUN = mean)
    pdat.bh[j, "reps"] = nrow(mycov)
    pdat.bh[j, "coverage"] = mean(mycov$x)
    mycov.ll = aggregate(x = tmp$coverage.ll, by = list(tmp$rep), FUN = mean)
    pdat.bh[j, "coverage.ll"] = mean(mycov.ll$x)
    mycov.ul = aggregate(x = tmp$coverage.ul, by = list(tmp$rep), FUN = mean)
    pdat.bh[j, "coverage.ul"] = mean(mycov.ul$x)
  }
}

# plot coverage of ci, 2, and bh
xvals = c(0.025, 0.075, seq(0.15, 0.95, 0.1))
pdat.ci[,"x.fdr"] = xvals
pdat.2[,"x.fdr"] = xvals
pdat.bh[,"x.fdr"] = xvals
pdat.ci[,"selection"] = "UCB"
pdat.2[,"selection"] = "M.2"
pdat.bh[,"selection"] = "BY"
vrs = c("x.fdr", "coverage", "reps", "selection")
plotdat = rbind(pdat.ci[1:7,vrs], pdat.2[1:7,vrs], pdat.bh[1:7,vrs])
plotdat[,"selection"] = factor(plotdat[,"selection"], levels=c("UCB", "M.2", "BY"))

p1 = ggplot(plotdat, aes(x = x.fdr)) +
  annotate("rect", xmin = 0, xmax = 0.56, ymin = 0.95, ymax = 1, alpha = .2, , fill = "grey") +
  geom_hline(yintercept = 0.95, linetype="twodash") +
  scale_x_continuous(limits = c(0, 0.56), expand = c(0,0)) +
  ggplot2::scale_y_continuous(limits = c(0.8, 1)) +
  theme_bw() + theme(panel.border = element_blank(), panel.grid.major = element_blank(),
                     panel.grid.minor = element_blank(), axis.line = element_line(colour = "black")) +
  geom_line(aes(y = coverage, linetype = plotdat$selection), size=1) +
  scale_colour_discrete("") +
  scale_linetype_manual("", values=c("dashed", "dotted", "solid")) +
  scale_shape_manual("", values=c(16,17,18)) +
  xlab("FDR") + theme(legend.position = c(0.25, 0.25)) +
  ylab("coverage") + ggplot2::labs(title = "conditional coverage (1 - FCR)") +
  theme(plot.margin = unit(c(0, 20, 10, 10), "pt")) +
  geom_point(aes(y = coverage, shape=selection), alpha=0.7, size=2)
p1
```

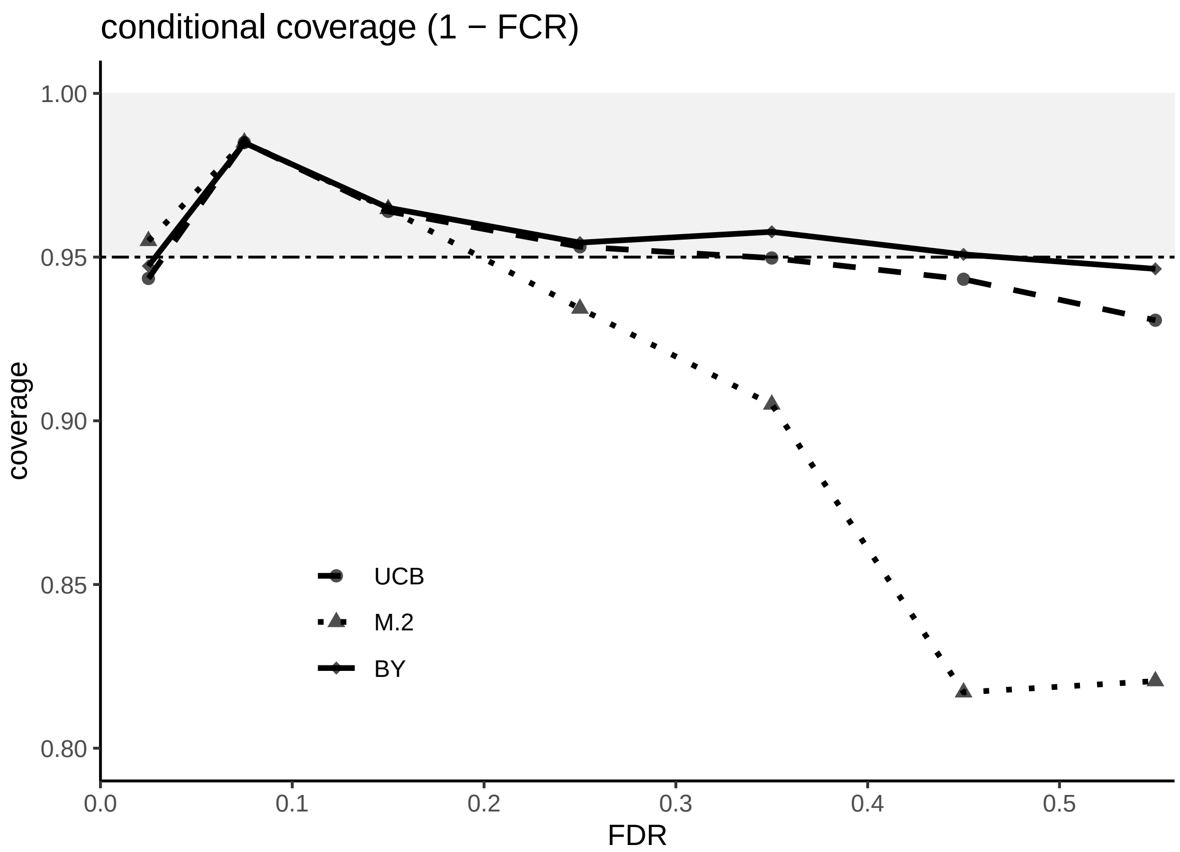


```
outfile = "Coverage_conditional_Fig1_v4.pdf"
pdf(outfile, width = 3.34646, height=3.3) # 3.34646 inches = 85 mm
grid.arrange( p1, ncol=1 )
dev.off()
```

```
pdf
  2
```

Simulations to evaluate MAVERICC trial permutation approach

Simulations were conducted to explore the statistical properties of likelihood ratio tests (LRT) of main effects and interactions in the context of Cox proportional hazards regression models following permutation. (See Supplemental Materials (Millstein et al. 2022) for more background.) In the MAVERICC clinical trial, we are interested in identifying genes whose expression interacts with treatment to affect survival. To generate results under the null hypothesis, we randomly permute the gene expression features. Here we use simulated data to assess type I error and power of the approach under several scenarios.

The first set of scenarios involves the situation where the gene is positively correlated with an adjustement covariate.

```
# myfun: function to find inverse of
# Z: myfun(X) = Z
# minX: lower bound of search
# maxX: upper bound of search
invfun = function(myfun, Z, minX, maxX, len0=100, mytol=1e-4, maxiter=1000){
  tol.tmp = 1
  maxiter.tmp = 0
  min.tmp = minX
```

```
  max.tmp = maxX
  while(tol.tmp > mytol && maxiter.tmp < maxiter){
    x.seq = seq(min.tmp, max.tmp, length.out=len0)
    v = myfun(x.seq)
    mvdif = which.min(abs(v - Z))
    X = x.seq[mvdif]

    if((mvdif == 1 | mvdif == len0) && maxiter.tmp == 0){
      print("Warning: min at boundary!")

      break
    }

    tol.tmp = abs(X - Z)
    min.tmp = x.seq[mvdif - 1]
    max.tmp = x.seq[mvdif + 1]
    maxiter.tmp = maxiter.tmp + 1
  }
  return(X)
}

# descretize TWAS features
descr = function(myvar, n.cats){
  probs = (0:n.cats) / n.cats
  qtls = quantile( myvar, probs=probs, na.rm=TRUE )
  myvar.c = cut(myvar, breaks=qtls, include.lowest = TRUE, ordered_result=TRUE)
  return(myvar.c)
}

### Continuous covariate, g (gene), binary covariate t (treatment)
gxe.sim = function(n, p.e=0.5, lambda=0.1, maxt, bh, bx, be, bg, beg ){
  E = rbinom(n, 1, p.e)
  H <- rnorm(n)
  G <- H*bh + rnorm(n)
  # descretize G?
  X = H*bh + rnorm(n)
  EG = E * G
  dat <- data.frame(X = X, G = G, E = E, EG = EG)
  tmp <- simsurv(dist="exponential", lambdas=lambda,
                x=dat, betas=c(X=bx, G=bg, E=be, EG=beg), maxt=maxt, )
  sdat = cbind(tmp[,c("eventtime", "status")], dat)
  return(sdat)
} # end gxe.sim

gxe.reg = function( mydat){
  nms = c("r.XG", "p.G", "p.EG", "p.2df", "p.G.perm", "p.EG.perm", "p.2df.perm")
  outp = rep(NA, length(nms))
  names(outp) = nms
  outp["r.XG"] = cor(mydat[,"X"], mydat[,"G"])

  my.surv = Surv( sdat[, "eventtime" ], mydat[, "status" ] )
  fit0 = coxph( my.surv ~ X + E + G, data=mydat)
  fit1 = coxph( my.surv ~ X + E + G + E:G, data=mydat)
```

```
  outp["p.G"] = anova(fit0)["G", "Pr(>|Chi|)"]
  outp["p.EG"] = anova(fit1)["E:G", "Pr(>|Chi|)"]
  outp["p.2df"] =  anova(fit0, fit1)[2,4]

  # permutation results
  mydat[,"G"] = sample(mydat[,"G"])
  my.surv = Surv( sdat[, "eventtime" ], mydat[, "status" ] )
  fit0 = coxph( my.surv ~ X + E + G, data=mydat)
  fit1 = coxph( my.surv ~ X + E + G + E:G, data=mydat)
  outp["p.G.perm"] = anova(fit0)["G", "Pr(>|Chi|)"]
  outp["p.EG.perm"] = anova(fit1)["E:G", "Pr(>|Chi|)"]
  outp["p.2df.perm"] =  anova(fit0, fit1)[2,4]

  return(outp)
} # end gxe.reg

# overall results for manuscript table
nms = c("betaG", "betaGT", "G.par", "GT.par", "G.perm", "GT.perm", "outliers", "G.cat")
tab.s1 = as.data.frame(matrix(NA, nrow=9, ncol=length(nms)))
names(tab.s1) = nms
rowno.s1 = 0
alpha = 0.05

# Conduct simulations under the null of beg = 0
n = 329
p.e = .5
maxt = 10
lambda = 0.2
bh = 1.5
bx = 0.2
be = 0.2
bg = 0.0
beg = 0.0
n.iter = 2000

rowno.s1 = rowno.s1 + 1
tab.s1[rowno.s1, "betaG"] = bg
tab.s1[rowno.s1, "betaGT"] = beg
tab.s1[rowno.s1, "outliers"] = 0
tab.s1[rowno.s1, "G.cat"] = 0

# results set-up
nms = c("n", "lambda", "maxt", "bh", "bx", "be", "bg", "beg",
        "p.G", "p.EG", "p.2df", "p.G.perm", "p.EG.perm", "p.2df.perm")
rslts = as.data.frame(matrix(NA, nrow=0, ncol=length(nms)))
names(rslts) = nms
nms.in = c("n", "lambda", "maxt", "bh", "bx", "be", "bg", "beg")
nms.out = c("r.XG", "p.G", "p.EG", "p.2df", "p.G.perm", "p.EG.perm", "p.2df.perm")

# run simulations
pflag = 0
for(i in 1:n.iter){
  if(floor(i/100) > pflag){
```

```
    #print(paste(Sys.time(), "Iter:", i))
    pflag = pflag + 1
  }
  sdat = gxe.sim(n=n, lambda=lambda, maxt=maxt, bh=bh, bx=bx, be=be, bg=bg, beg=beg)
  rslts[i, nms.in] = c(n, lambda, maxt, bh, bx, be, bg, beg)
  rslts[i, nms.out] = gxe.reg(sdat)
}

# type I error for parametric
tab.s1[rowno.s1, "G.par"] = sum(rslts$p.G < alpha) / n.iter
tab.s1[rowno.s1, "GT.par"] = sum(rslts$p.EG < alpha) / n.iter

# type I error for non-parametric
pdistn = ecdf(rslts[,"p.G.perm"])
tmpX = invfun(pdistn, 0.05, 0.03, 0.07)
tab.s1[rowno.s1, "G.perm"] = sum(rslts$p.G < tmpX) / n.iter

pdistn = ecdf(rslts[,"p.EG.perm"])
tmpX = invfun(pdistn, 0.05, 0.03, 0.07)
tab.s1[rowno.s1, "GT.perm"] = sum(rslts$p.EG < tmpX) / n.iter

# Conduct simulations under the null of beg = 0
n = 329
p.e = .5
maxt = 10
lambda = 0.2
bh = 1.5
bx = 0.2
be = 0.2
bg = 0.2
beg = 0.0

rowno.s1 = rowno.s1 + 1
tab.s1[rowno.s1, "betaG"] = bg
tab.s1[rowno.s1, "betaGT"] = beg
tab.s1[rowno.s1, "outliers"] = 0
tab.s1[rowno.s1, "G.cat"] = 0

# results set-up
nms = c("n", "lambda", "maxt", "bh", "bx", "be", "bg", "beg",
        "p.G", "p.EG", "p.2df", "p.G.perm", "p.EG.perm", "p.2df.perm")
rslts = as.data.frame(matrix(NA, nrow=0, ncol=length(nms)))
names(rslts) = nms
nms.in = c("n", "lambda", "maxt", "bh", "bx", "be", "bg", "beg")
nms.out = c("r.XG", "p.G", "p.EG", "p.2df", "p.G.perm", "p.EG.perm", "p.2df.perm")

# run simulations
pflag = 0
for(i in 1:n.iter){
  if(floor(i/100) > pflag){
    #print(paste(Sys.time(), "Iter:", i))
    pflag = pflag + 1
  }
```

```
  sdat = gxe.sim(n=n, lambda=lambda, maxt=maxt, bh=bh, bx=bx, be=be, bg=bg, beg=beg)
  rslts[i, nms.in] = c(n, lambda, maxt, bh, bx, be, bg, beg)
  rslts[i, nms.out] = gxe.reg(sdat)
}

# type I error for parametric
tab.s1[rowno.s1, "G.par"] = sum(rslts$p.G < alpha) / n.iter
tab.s1[rowno.s1, "GT.par"] = sum(rslts$p.EG < alpha) / n.iter

# type I error for non-parametric
pdistn = ecdf(rslts[,"p.G.perm"])
tmpX = invfun(pdistn, 0.05, 0.03, 0.07)
tab.s1[rowno.s1, "G.perm"] = sum(rslts$p.G < tmpX) / n.iter

pdistn = ecdf(rslts[,"p.EG.perm"])
tmpX = invfun(pdistn, 0.05, 0.03, 0.07)
tab.s1[rowno.s1, "GT.perm"] = sum(rslts$p.EG < tmpX) / n.iter

# Conduct simulations under the alternative of beg != 0
n = 329
p.e = .5
maxt = 10
lambda = 0.2
bh = 1.5
bx = 0.2
be = 0.2
bg = 0.2
beg = 0.2

rowno.s1 = rowno.s1 + 1
tab.s1[rowno.s1, "betaG"] = bg
tab.s1[rowno.s1, "betaGT"] = beg
tab.s1[rowno.s1, "outliers"] = 0
tab.s1[rowno.s1, "G.cat"] = 0

# results set-up
nms = c("n", "lambda", "maxt", "bh", "bx", "be", "bg", "beg",
        "p.G", "p.EG", "p.2df", "p.G.perm", "p.EG.perm", "p.2df.perm")
rslts = as.data.frame(matrix(NA, nrow=0, ncol=length(nms)))
names(rslts) = nms
nms.in = c("n", "lambda", "maxt", "bh", "bx", "be", "bg", "beg")
nms.out = c("r.XG", "p.G", "p.EG", "p.2df", "p.G.perm", "p.EG.perm", "p.2df.perm")

# run simulations
pflag = 0
for(i in 1:n.iter){
  if(floor(i/100) > pflag){
    #print(paste(Sys.time(), "Iter:", i))
    pflag = pflag + 1
  }
  sdat = gxe.sim(n=n, lambda=lambda, maxt=maxt, bh=bh, bx=bx, be=be, bg=bg, beg=beg)
  rslts[i, nms.in] = c(n, lambda, maxt, bh, bx, be, bg, beg)
  rslts[i, nms.out] = gxe.reg(sdat)
```

```
}

# type I error for parametric
tab.s1[rowno.s1, "G.par"] = sum(rslts$p.G < alpha) / n.iter
tab.s1[rowno.s1, "GT.par"] = sum(rslts$p.EG < alpha) / n.iter

# type I error for non-parametric
pdistn = ecdf(rslts[,"p.G.perm"])
tmpX = invfun(pdistn, 0.05, 0.03, 0.07)
tab.s1[rowno.s1, "G.perm"] = sum(rslts$p.G < tmpX) / n.iter

pdistn = ecdf(rslts[,"p.EG.perm"])
tmpX = invfun(pdistn, 0.05, 0.03, 0.07)
tab.s1[rowno.s1, "GT.perm"] = sum(rslts$p.EG < tmpX) / n.iter

# add random outliers to G and event time
### Continuous covariate, g (gene), binary covariate t (treatment)
gxe.sim.outl = function(n, p.e=0.5, lambda=0.1, maxt, bh, bx, be, bg, beg, n.outl=5 ){
  E = rbinom(n, 1, p.e)
  H <- rnorm(n)
  G <- H*bh + rnorm(n)
  # descretize G?
  X = H*bh + rnorm(n)
  EG = E * G
  dat <- data.frame(X = X, G = G, E = E, EG = EG)
  tmp <- simsurv(dist="exponential", lambdas=lambda,
               x=dat, betas=c(X=bx, G=bg, E=be, EG=beg), maxt=maxt, )
  sdat = cbind(tmp[,c("eventtime", "status")], dat)

  # add extreme outliers to G
  se.o = sd(sdat[,"G"])
  mn.o = mean(sdat[,"G"]) + 5 * se.o
  outlrs = rnorm(n.outl, mn.o, se.o)
  ind = sample(1:n, n.outl)
  sdat[ind, "G"] = outlrs

  # add extreme outliers to eventtime
  ind = sample(1:n, n.outl)
  sdat[ind, "eventtime"] = sdat[ind, "eventtime"] * 5

  return(sdat)
} # end gxe.sim.outl

# Outliers in G and eventtime under the null of beg = 0
n = 329
p.e = .5
maxt = 10
lambda = 0.2
bh = 1.5
bx = 0.2
be = 0.2
bg = 0.0
beg = 0.0
```

```
rowno.s1 = rowno.s1 + 1
tab.s1[rowno.s1, "betaG"] = bg
tab.s1[rowno.s1, "betaGT"] = beg
tab.s1[rowno.s1, "outliers"] = 1
tab.s1[rowno.s1, "G.cat"] = 0

# results set-up
nms = c("n", "lambda", "maxt", "bh", "bx", "be", "bg", "beg",
        "p.G", "p.EG", "p.2df", "p.G.perm", "p.EG.perm", "p.2df.perm")
rslts = as.data.frame(matrix(NA, nrow=0, ncol=length(nms)))
names(rslts) = nms
nms.in = c("n", "lambda", "maxt", "bh", "bx", "be", "bg", "beg")
nms.out = c("r.XG", "p.G", "p.EG", "p.2df", "p.G.perm", "p.EG.perm", "p.2df.perm")

# run simulations
pflag = 0
for(i in 1:n.iter){
  if(floor(i/100) > pflag){
    #print(paste(Sys.time(), "Iter:", i))
    pflag = pflag + 1
  }
  sdat = gxe.sim.outl(n=n, lambda=lambda, maxt=maxt, bh=bh, bx=bx, be=be, bg=bg, beg=beg)
  rslts[i, nms.in] = c(n, lambda, maxt, bh, bx, be, bg, beg)
  rslts[i, nms.out] = gxe.reg(sdat)
}

# type I error for parametric
tab.s1[rowno.s1, "G.par"] = sum(rslts$p.G < alpha) / n.iter # 0.047
tab.s1[rowno.s1, "GT.par"] = sum(rslts$p.EG < alpha) / n.iter

# type I error for non-parametric
pdistn = ecdf(rslts[,"p.G.perm"])
tmpX = invfun(pdistn, 0.05, 0.03, 0.07)
tab.s1[rowno.s1, "G.perm"] = sum(rslts$p.G < tmpX) / n.iter

pdistn = ecdf(rslts[,"p.EG.perm"])
tmpX = invfun(pdistn, 0.05, 0.03, 0.07)
tab.s1[rowno.s1, "GT.perm"] = sum(rslts$p.EG < tmpX) / n.iter

# Outliers in G and eventtime under the null of beg = 0
n = 329 # updated from 167
p.e = .5
maxt = 10
lambda = 0.2
bh = 1.5
bx = 0.2
be = 0.2
bg = 0.2
beg = 0.0

rowno.s1 = rowno.s1 + 1
tab.s1[rowno.s1, "betaG"] = bg
tab.s1[rowno.s1, "betaGT"] = beg
```

```
tab.s1[rowno.s1, "outliers"] = 1
tab.s1[rowno.s1, "G.cat"] = 0

# results set-up
nms = c("n", "lambda", "maxt", "bh", "bx", "be", "bg", "beg",
        "p.G", "p.EG", "p.2df", "p.G.perm", "p.EG.perm", "p.2df.perm")
rslts = as.data.frame(matrix(NA, nrow=0, ncol=length(nms)))
names(rslts) = nms
nms.in = c("n", "lambda", "maxt", "bh", "bx", "be", "bg", "beg")
nms.out = c("r.XG", "p.G", "p.EG", "p.2df", "p.G.perm", "p.EG.perm", "p.2df.perm")

# run simulations
pflag = 0
for(i in 1:n.iter){
  if(floor(i/100) > pflag){
    #print(paste(Sys.time(), "Iter:", i))
    pflag = pflag + 1
  }
  sdat = gxe.sim.outl(n=n, lambda=lambda, maxt=maxt, bh=bh, bx=bx, be=be, bg=bg, beg=beg)
  rslts[i, nms.in] = c(n, lambda, maxt, bh, bx, be, bg, beg)
  rslts[i, nms.out] = gxe.reg(sdat)
}

# type I error for parametric
tab.s1[rowno.s1, "G.par"] = sum(rslts$p.G < alpha) / n.iter
tab.s1[rowno.s1, "GT.par"] = sum(rslts$p.EG < alpha) / n.iter

# type I error for non-parametric
pdistn = ecdf(rslts[,"p.G.perm"])
tmpX = invfun(pdistn, 0.05, 0.03, 0.07)
tab.s1[rowno.s1, "G.perm"] = sum(rslts$p.G < tmpX) / n.iter

pdistn = ecdf(rslts[,"p.EG.perm"])
tmpX = invfun(pdistn, 0.05, 0.03, 0.07)
tab.s1[rowno.s1, "GT.perm"] = sum(rslts$p.EG < tmpX) / n.iter

# Outliers in G and eventtime under the null of beg != 0
n = 329
p.e = .5
maxt = 10
lambda = 0.2
bh = 1.5
bx = 0.2
be = 0.2
bg = 0.2
beg = 0.2

rowno.s1 = rowno.s1 + 1
tab.s1[rowno.s1, "betaG"] = bg
tab.s1[rowno.s1, "betaGT"] = beg
tab.s1[rowno.s1, "outliers"] = 1
tab.s1[rowno.s1, "G.cat"] = 0
```

```
# results set-up
nms = c("n", "lambda", "maxt", "bh", "bx", "be", "bg", "beg",
        "p.G", "p.EG", "p.2df", "p.G.perm", "p.EG.perm", "p.2df.perm")
rslts = as.data.frame(matrix(NA, nrow=0, ncol=length(nms)))
names(rslts) = nms
nms.in = c("n", "lambda", "maxt", "bh", "bx", "be", "bg", "beg")
nms.out = c("r.XG", "p.G", "p.EG", "p.2df", "p.G.perm", "p.EG.perm", "p.2df.perm")

# run simulations
pflag = 0
for(i in 1:n.iter){
  if(floor(i/100) > pflag){
    #print(paste(Sys.time(), "Iter:", i))
    pflag = pflag + 1
  }
  sdat = gxe.sim.outl(n=n, lambda=lambda, maxt=maxt, bh=bh, bx=bx, be=be, bg=bg, beg=beg)
  rslts[i, nms.in] = c(n, lambda, maxt, bh, bx, be, bg, beg)
  rslts[i, nms.out] = gxe.reg(sdat)
}

# type I error for parametric
tab.s1[rowno.s1, "G.par"] = sum(rslts$p.G < alpha) / n.iter
tab.s1[rowno.s1, "GT.par"] = sum(rslts$p.EG < alpha) / n.iter

# type I error for non-parametric
pdistn = ecdf(rslts[,"p.G.perm"])
tmpX = invfun(pdistn, 0.05, 0.03, 0.07)
tab.s1[rowno.s1, "G.perm"] = sum(rslts$p.G < tmpX) / n.iter

pdistn = ecdf(rslts[,"p.EG.perm"])
tmpX = invfun(pdistn, 0.05, 0.03, 0.07)
tab.s1[rowno.s1, "GT.perm"] = sum(rslts$p.EG < tmpX) / n.iter

# add random outliers to G and event time, but then descretize G
### Continuous covariate, g (gene), binary covariate t (treatment)
gxe.sim.outl = function(n, p.e=0.5, lambda=0.1, maxt, bh, bx, be, bg, beg, n.outl=5 ){
  E = rbinom(n, 1, p.e)
  H <- rnorm(n)
  G <- H*bh + rnorm(n)
  # descretize G?
  X = H*bh + rnorm(n)
  EG = E * G
  dat <- data.frame(X = X, G = G, E = E, EG = EG)
  tmp <- simsurv(dist="exponential", lambdas=lambda,
                 x=dat, betas=c(X=bx, G=bg, E=be, EG=beg), maxt=maxt, )
  sdat = cbind(tmp[,c("eventtime", "status")], dat)

  # add extreme outliers to G
  se.o = sd(sdat[,"G"])
  mn.o = mean(sdat[,"G"]) + 5 * se.o
  outlrs = rnorm(n.outl, mn.o, se.o)
  ind = sample(1:n, n.outl)
  sdat[ind, "G"] = outlrs
```

```r
  # descretize G
  sdat[,"G"] = as.integer(descr(sdat[,"G"], 3))

  # add extreme outliers to eventtime
  ind = sample(1:n, n.outl)
  sdat[ind, "eventtime"] = sdat[ind, "eventtime"] * 5

  return(sdat)
} # end gxe.sim.outl

# Outliers in G and eventtime under the null of beg = 0, bg = 0
n = 329
p.e = .5
maxt = 10
lambda = 0.2
bh = 1.5
bx = 0.2
be = 0.2
bg = 0.0
beg = 0.0

rowno.s1 = rowno.s1 + 1
tab.s1[rowno.s1, "betaG"] = bg
tab.s1[rowno.s1, "betaGT"] = beg
tab.s1[rowno.s1, "outliers"] = 1
tab.s1[rowno.s1, "G.cat"] = 1

# results set-up
nms = c("n", "lambda", "maxt", "bh", "bx", "be", "bg", "beg",
        "p.G", "p.EG", "p.2df", "p.G.perm", "p.EG.perm", "p.2df.perm")
rslts = as.data.frame(matrix(NA, nrow=0, ncol=length(nms)))
names(rslts) = nms
nms.in = c("n", "lambda", "maxt", "bh", "bx", "be", "bg", "beg")
nms.out = c("r.XG", "p.G", "p.EG", "p.2df", "p.G.perm", "p.EG.perm", "p.2df.perm")

# run simulations
pflag = 0
for(i in 1:n.iter){
  if(floor(i/100) > pflag){
    #print(paste(Sys.time(), "Iter:", i))
    pflag = pflag + 1
  }
  sdat = gxe.sim.outl(n=n, lambda=lambda, maxt=maxt, bh=bh, bx=bx, be=be, bg=bg, beg=beg)
  rslts[i, nms.in] = c(n, lambda, maxt, bh, bx, be, bg, beg)
  rslts[i, nms.out] = gxe.reg(sdat)
}

# type I error for parametric
tab.s1[rowno.s1, "G.par"] = sum(rslts$p.G < alpha) / n.iter
tab.s1[rowno.s1, "GT.par"] = sum(rslts$p.EG < alpha) / n.iter

# type I error for non-parametric
pdistn = ecdf(rslts[,"p.G.perm"])
```

```
tmpX = invfun(pdistn, 0.05, 0.03, 0.07)
tab.s1[rowno.s1, "G.perm"] = sum(rslts$p.G < tmpX) / n.iter

pdistn = ecdf(rslts[,"p.EG.perm"])
tmpX = invfun(pdistn, 0.05, 0.03, 0.07)
tab.s1[rowno.s1, "GT.perm"] = sum(rslts$p.EG < tmpX) / n.iter

# Outliers in G and eventtime under the null of beg = 0
n = 329
p.e = .5
maxt = 10
lambda = 0.2
bh = 1.5
bx = 0.2
be = 0.2
bg = 0.2
beg = 0.0

rowno.s1 = rowno.s1 + 1
tab.s1[rowno.s1, "betaG"] = bg
tab.s1[rowno.s1, "betaGT"] = beg
tab.s1[rowno.s1, "outliers"] = 1
tab.s1[rowno.s1, "G.cat"] = 1

# results set-up
nms = c("n", "lambda", "maxt", "bh", "bx", "be", "bg", "beg",
        "p.G", "p.EG", "p.2df", "p.G.perm", "p.EG.perm", "p.2df.perm")
rslts = as.data.frame(matrix(NA, nrow=0, ncol=length(nms)))
names(rslts) = nms
nms.in = c("n", "lambda", "maxt", "bh", "bx", "be", "bg", "beg")
nms.out = c("r.XG", "p.G", "p.EG", "p.2df", "p.G.perm", "p.EG.perm", "p.2df.perm")

# run simulations
pflag = 0
for(i in 1:n.iter){
  if(floor(i/100) > pflag){
    #print(paste(Sys.time(), "Iter:", i))
    pflag = pflag + 1
  }
  sdat = gxe.sim.outl(n=n, lambda=lambda, maxt=maxt, bh=bh, bx=bx, be=be, bg=bg, beg=beg)
  rslts[i, nms.in] = c(n, lambda, maxt, bh, bx, be, bg, beg)
  rslts[i, nms.out] = gxe.reg(sdat)
}

# type I error for parametric
tab.s1[rowno.s1, "G.par"] = sum(rslts$p.G < alpha) / n.iter
tab.s1[rowno.s1, "GT.par"] = sum(rslts$p.EG < alpha) / n.iter

# type I error for non-parametric
pdistn = ecdf(rslts[,"p.G.perm"])
tmpX = invfun(pdistn, 0.05, 0.03, 0.07)
tab.s1[rowno.s1, "G.perm"] = sum(rslts$p.G < tmpX) / n.iter
```

```r
pdistn = ecdf(rslts[,"p.EG.perm"])
tmpX = invfun(pdistn, 0.05, 0.03, 0.07)
tab.s1[rowno.s1, "GT.perm"] = sum(rslts$p.EG < tmpX) / n.iter

# Outliers in G and eventtime under the null of beg != 0
n = 329
p.e = .5
maxt = 10
lambda = 0.2
bh = 1.5
bx = 0.2
be = 0.2
bg = 0.2
beg = 0.2

rowno.s1 = rowno.s1 + 1
tab.s1[rowno.s1, "betaG"] = bg
tab.s1[rowno.s1, "betaGT"] = beg
tab.s1[rowno.s1, "outliers"] = 1
tab.s1[rowno.s1, "G.cat"] = 1

# results set-up
nms = c("n", "lambda", "maxt", "bh", "bx", "be", "bg", "beg",
        "p.G", "p.EG", "p.2df", "p.G.perm", "p.EG.perm", "p.2df.perm")
rslts = as.data.frame(matrix(NA, nrow=0, ncol=length(nms)))
names(rslts) = nms
nms.in = c("n", "lambda", "maxt", "bh", "bx", "be", "bg", "beg")
nms.out = c("r.XG", "p.G", "p.EG", "p.2df", "p.G.perm", "p.EG.perm", "p.2df.perm")

# run simulations
pflag = 0
for(i in 1:n.iter){
  if(floor(i/100) > pflag){
    #print(paste(Sys.time(), "Iter:", i))
    pflag = pflag + 1
  }
  sdat = gxe.sim.outl(n=n, lambda=lambda, maxt=maxt, bh=bh, bx=bx, be=be, bg=bg, beg=beg)
  rslts[i, nms.in] = c(n, lambda, maxt, bh, bx, be, bg, beg)
  rslts[i, nms.out] = gxe.reg(sdat)
}

# type I error for parametric
tab.s1[rowno.s1, "G.par"] = sum(rslts$p.G < alpha) / n.iter
tab.s1[rowno.s1, "GT.par"] = sum(rslts$p.EG < alpha) / n.iter

# type I error for non-parametric
pdistn = ecdf(rslts[,"p.G.perm"])
tmpX = invfun(pdistn, 0.05, 0.03, 0.07)
tab.s1[rowno.s1, "G.perm"] = sum(rslts$p.G < tmpX) / n.iter

pdistn = ecdf(rslts[,"p.EG.perm"])
tmpX = invfun(pdistn, 0.05, 0.03, 0.07)
tab.s1[rowno.s1, "GT.perm"] = sum(rslts$p.EG < tmpX) / n.iter
```

```r
# Supplementary table S1.
knitr::kable(tab.s1, caption = "Type I error and statistical power for main effects and interactions",
             digits=3)
```

Table 2: Type I error and statistical power for main effects and interactions

betaG	betaGT	G.par	GT.par	G.perm	GT.perm	outliers	G.cat
0.0	0.0	0.046	0.052	0.049	0.047	0	0
0.2	0.0	0.988	0.055	0.986	0.050	0	0
0.2	0.2	1.000	0.780	1.000	0.760	0	0
0.0	0.0	0.048	0.046	0.051	0.039	1	0
0.2	0.0	0.818	0.111	0.828	0.093	1	0
0.2	0.2	0.967	0.420	0.973	0.389	1	0
0.0	0.0	0.036	0.052	0.049	0.045	1	1
0.2	0.0	0.896	0.051	0.914	0.047	1	1
0.2	0.2	0.998	0.566	0.998	0.518	1	1

The second set of scenarios involves the situation where the gene is negatively correlated with an adjustment covariate.

```r
# overall results for manuscript table
nms = c("betaG", "betaGT", "G.par", "GT.par", "G.perm", "GT.perm", "outliers", "G.cat")
tab.s2 = as.data.frame(matrix(NA, nrow=9, ncol=length(nms)))
names(tab.s2) = nms
rowno.s1 = 0
alpha = 0.05

# Conduct simulations under the null of beg = 0
n = 329
p.e = .5
maxt = 10
lambda = 0.2
bh = -1.5
bx = 0.2
be = 0.2
bg = 0.0
beg = 0.0

rowno.s1 = rowno.s1 + 1
tab.s2[rowno.s1, "betaG"] = bg
tab.s2[rowno.s1, "betaGT"] = beg
tab.s2[rowno.s1, "outliers"] = 0
tab.s2[rowno.s1, "G.cat"] = 0

# results set-up
nms = c("n", "lambda", "maxt", "bh", "bx", "be", "bg", "beg",
        "p.G", "p.EG", "p.2df", "p.G.perm", "p.EG.perm", "p.2df.perm")
rslts = as.data.frame(matrix(NA, nrow=0, ncol=length(nms)))
names(rslts) = nms
nms.in = c("n", "lambda", "maxt", "bh", "bx", "be", "bg", "beg")
nms.out = c("r.XG", "p.G", "p.EG", "p.2df", "p.G.perm", "p.EG.perm", "p.2df.perm")
```

```
# run simulations
pflag = 0
for(i in 1:n.iter){
  if(floor(i/100) > pflag){
    #print(paste(Sys.time(), "Iter:", i))
    pflag = pflag + 1
  }
  sdat = gxe.sim(n=n, lambda=lambda, maxt=maxt, bh=bh, bx=bx, be=be, bg=bg, beg=beg)
  rslts[i, nms.in] = c(n, lambda, maxt, bh, bx, be, bg, beg)
  rslts[i, nms.out] = gxe.reg(sdat)
}

# type I error for parametric
tab.s2[rowno.s1, "G.par"] = sum(rslts$p.G < alpha) / n.iter
tab.s2[rowno.s1, "GT.par"] = sum(rslts$p.EG < alpha) / n.iter

# type I error for non-parametric
pdistn = ecdf(rslts[,"p.G.perm"])
tmpX = invfun(pdistn, 0.05, 0.03, 0.07)
tab.s2[rowno.s1, "G.perm"] = sum(rslts$p.G < tmpX) / n.iter

pdistn = ecdf(rslts[,"p.EG.perm"])
tmpX = invfun(pdistn, 0.05, 0.03, 0.07)
tab.s2[rowno.s1, "GT.perm"] = sum(rslts$p.EG < tmpX) / n.iter

# Conduct simulations under the null of beg = 0
n = 329
p.e = .5
maxt = 10
lambda = 0.2
bh = -1.5
bx = 0.2
be = 0.2
bg = 0.2
beg = 0.0

rowno.s1 = rowno.s1 + 1
tab.s2[rowno.s1, "betaG"] = bg
tab.s2[rowno.s1, "betaGT"] = beg
tab.s2[rowno.s1, "outliers"] = 0
tab.s2[rowno.s1, "G.cat"] = 0

# results set-up
nms = c("n", "lambda", "maxt", "bh", "bx", "be", "bg", "beg",
        "p.G", "p.EG", "p.2df", "p.G.perm", "p.EG.perm", "p.2df.perm")
rslts = as.data.frame(matrix(NA, nrow=0, ncol=length(nms)))
names(rslts) = nms
nms.in = c("n", "lambda", "maxt", "bh", "bx", "be", "bg", "beg")
nms.out = c("r.XG", "p.G", "p.EG", "p.2df", "p.G.perm", "p.EG.perm", "p.2df.perm")

# run simulations
pflag = 0
for(i in 1:n.iter){
```

```
  if(floor(i/100) > pflag){
    #print(paste(Sys.time(), "Iter:", i))
    pflag = pflag + 1
  }
  sdat = gxe.sim(n=n, lambda=lambda, maxt=maxt, bh=bh, bx=bx, be=be, bg=bg, beg=beg)
  rslts[i, nms.in] = c(n, lambda, maxt, bh, bx, be, bg, beg)
  rslts[i, nms.out] = gxe.reg(sdat)
}

# type I error for parametric
tab.s2[rowno.s1, "G.par"] = sum(rslts$p.G < alpha) / n.iter
tab.s2[rowno.s1, "GT.par"] = sum(rslts$p.EG < alpha) / n.iter

# type I error for non-parametric
pdistn = ecdf(rslts[,"p.G.perm"])
tmpX = invfun(pdistn, 0.05, 0.03, 0.07)
tab.s2[rowno.s1, "G.perm"] = sum(rslts$p.G < tmpX) / n.iter

pdistn = ecdf(rslts[,"p.EG.perm"])
tmpX = invfun(pdistn, 0.05, 0.03, 0.07)
tab.s2[rowno.s1, "GT.perm"] = sum(rslts$p.EG < tmpX) / n.iter

# Conduct simulations under the alternative of beg != 0
n = 329
p.e = .5
maxt = 10
lambda = 0.2
bh = -1.5
bx = 0.2
be = 0.2
bg = 0.2
beg = 0.2

rowno.s1 = rowno.s1 + 1
tab.s2[rowno.s1, "betaG"] = bg
tab.s2[rowno.s1, "betaGT"] = beg
tab.s2[rowno.s1, "outliers"] = 0
tab.s2[rowno.s1, "G.cat"] = 0

# results set-up
nms = c("n", "lambda", "maxt", "bh", "bx", "be", "bg", "beg",
        "p.G", "p.EG", "p.2df", "p.G.perm", "p.EG.perm", "p.2df.perm")
rslts = as.data.frame(matrix(NA, nrow=0, ncol=length(nms)))
names(rslts) = nms
nms.in = c("n", "lambda", "maxt", "bh", "bx", "be", "bg", "beg")
nms.out = c("r.XG", "p.G", "p.EG", "p.2df", "p.G.perm", "p.EG.perm", "p.2df.perm")

# run simulations
pflag = 0
for(i in 1:n.iter){
  if(floor(i/100) > pflag){
    #print(paste(Sys.time(), "Iter:", i))
    pflag = pflag + 1
```

```
  }
  sdat = gxe.sim(n=n, lambda=lambda, maxt=maxt, bh=bh, bx=bx, be=be, bg=bg, beg=beg)
  rslts[i, nms.in] = c(n, lambda, maxt, bh, bx, be, bg, beg)
  rslts[i, nms.out] = gxe.reg(sdat)
}

# type I error for parametric
tab.s2[rowno.s1, "G.par"] = sum(rslts$p.G < alpha) / n.iter
tab.s2[rowno.s1, "GT.par"] = sum(rslts$p.EG < alpha) / n.iter

# type I error for non-parametric
pdistn = ecdf(rslts[,"p.G.perm"])
tmpX = invfun(pdistn, 0.05, 0.03, 0.07)
tab.s2[rowno.s1, "G.perm"] = sum(rslts$p.G < tmpX) / n.iter

pdistn = ecdf(rslts[,"p.EG.perm"])
tmpX = invfun(pdistn, 0.05, 0.03, 0.07)
tab.s2[rowno.s1, "GT.perm"] = sum(rslts$p.EG < tmpX) / n.iter

# add random outliers to G and event time
### Continuous covariate, g (gene), binary covariate t (treatment)
gxe.sim.outl = function(n, p.e=0.5, lambda=0.1, maxt, bh, bx, be, bg, beg, n.outl=5 ){
  E = rbinom(n, 1, p.e)
  H <- rnorm(n)
  G <- H*bh + rnorm(n)
  # descretize G?
  X = H*bh + rnorm(n)
  EG = E * G
  dat <- data.frame(X = X, G = G, E = E, EG = EG)
  tmp <- simsurv(dist="exponential", lambdas=lambda,
                 x=dat, betas=c(X=bx, G=bg, E=be, EG=beg), maxt=maxt, )
  sdat = cbind(tmp[,c("eventtime", "status")], dat)

  # add extreme outliers to G
  se.o = sd(sdat[,"G"])
  mn.o = mean(sdat[,"G"]) + 5 * se.o
  outlrs = rnorm(n.outl, mn.o, se.o)
  ind = sample(1:n, n.outl)
  sdat[ind, "G"] = outlrs

  # add extreme outliers to eventtime
  ind = sample(1:n, n.outl)
  sdat[ind, "eventtime"] = sdat[ind, "eventtime"] * 5

  return(sdat)
} # end gxe.sim.outl

# Outliers in G and eventtime under the null of beg = 0
n = 329
p.e = .5
maxt = 10
lambda = 0.2
bh = -1.5
```

```
bx = 0.2
be = 0.2
bg = 0.0
beg = 0.0

rowno.s1 = rowno.s1 + 1
tab.s2[rowno.s1, "betaG"] = bg
tab.s2[rowno.s1, "betaGT"] = beg
tab.s2[rowno.s1, "outliers"] = 1
tab.s2[rowno.s1, "G.cat"] = 0

# results set-up
nms = c("n", "lambda", "maxt", "bh", "bx", "be", "bg", "beg",
        "p.G", "p.EG", "p.2df", "p.G.perm", "p.EG.perm", "p.2df.perm")
rslts = as.data.frame(matrix(NA, nrow=0, ncol=length(nms)))
names(rslts) = nms
nms.in = c("n", "lambda", "maxt", "bh", "bx", "be", "bg", "beg")
nms.out = c("r.XG", "p.G", "p.EG", "p.2df", "p.G.perm", "p.EG.perm", "p.2df.perm")

# run simulations
pflag = 0
for(i in 1:n.iter){
  if(floor(i/100) > pflag){
    #print(paste(Sys.time(), "Iter:", i))
    pflag = pflag + 1
  }
  sdat = gxe.sim.outl(n=n, lambda=lambda, maxt=maxt, bh=bh, bx=bx, be=be, bg=bg, beg=beg)
  rslts[i, nms.in] = c(n, lambda, maxt, bh, bx, be, bg, beg)
  rslts[i, nms.out] = gxe.reg(sdat)
}

# type I error for parametric
tab.s2[rowno.s1, "G.par"] = sum(rslts$p.G < alpha) / n.iter
tab.s2[rowno.s1, "GT.par"] = sum(rslts$p.EG < alpha) / n.iter

# type I error for non-parametric
pdistn = ecdf(rslts[,"p.G.perm"])
tmpX = invfun(pdistn, 0.05, 0.03, 0.07)
tab.s2[rowno.s1, "G.perm"] = sum(rslts$p.G < tmpX) / n.iter

pdistn = ecdf(rslts[,"p.EG.perm"])
tmpX = invfun(pdistn, 0.05, 0.03, 0.07)
tab.s2[rowno.s1, "GT.perm"] = sum(rslts$p.EG < tmpX) / n.iter

# Outliers in G and eventtime under the null of beg = 0
n = 329
p.e = .5
maxt = 10
lambda = 0.2
bh = -1.5
bx = 0.2
be = 0.2
bg = 0.2
```

```
beg = 0.0

rowno.s1 = rowno.s1 + 1
tab.s2[rowno.s1, "betaG"] = bg
tab.s2[rowno.s1, "betaGT"] = beg
tab.s2[rowno.s1, "outliers"] = 1
tab.s2[rowno.s1, "G.cat"] = 0

# results set-up
nms = c("n", "lambda", "maxt", "bh", "bx", "be", "bg", "beg",
        "p.G", "p.EG", "p.2df", "p.G.perm", "p.EG.perm", "p.2df.perm")
rslts = as.data.frame(matrix(NA, nrow=0, ncol=length(nms)))
names(rslts) = nms
nms.in = c("n", "lambda", "maxt", "bh", "bx", "be", "bg", "beg")
nms.out = c("r.XG", "p.G", "p.EG", "p.2df", "p.G.perm", "p.EG.perm", "p.2df.perm")

# run simulations
pflag = 0
for(i in 1:n.iter){
  if(floor(i/100) > pflag){
    #print(paste(Sys.time(), "Iter:", i))
    pflag = pflag + 1
  }
  sdat = gxe.sim.outl(n=n, lambda=lambda, maxt=maxt, bh=bh, bx=bx, be=be, bg=bg, beg=beg)
  rslts[i, nms.in] = c(n, lambda, maxt, bh, bx, be, bg, beg)
  rslts[i, nms.out] = gxe.reg(sdat)
}

# type I error for parametric
tab.s2[rowno.s1, "G.par"] = sum(rslts$p.G < alpha) / n.iter
tab.s2[rowno.s1, "GT.par"] = sum(rslts$p.EG < alpha) / n.iter

# type I error for non-parametric
pdistn = ecdf(rslts[,"p.G.perm"])
tmpX = invfun(pdistn, 0.05, 0.03, 0.07)
tab.s2[rowno.s1, "G.perm"] = sum(rslts$p.G < tmpX) / n.iter

pdistn = ecdf(rslts[,"p.EG.perm"])
tmpX = invfun(pdistn, 0.05, 0.03, 0.07)
tab.s2[rowno.s1, "GT.perm"] = sum(rslts$p.EG < tmpX) / n.iter

# Outliers in G and eventtime under the null of beg != 0
n = 329
p.e = .5
maxt = 10
lambda = 0.2
bh = -1.5
bx = 0.2
be = 0.2
bg = 0.2
beg = 0.2

rowno.s1 = rowno.s1 + 1
```

```r
tab.s2[rowno.s1, "betaG"] = bg
tab.s2[rowno.s1, "betaGT"] = beg
tab.s2[rowno.s1, "outliers"] = 1
tab.s2[rowno.s1, "G.cat"] = 0

# results set-up
nms = c("n", "lambda", "maxt", "bh", "bx", "be", "bg", "beg",
        "p.G", "p.EG", "p.2df", "p.G.perm", "p.EG.perm", "p.2df.perm")
rslts = as.data.frame(matrix(NA, nrow=0, ncol=length(nms)))
names(rslts) = nms
nms.in = c("n", "lambda", "maxt", "bh", "bx", "be", "bg", "beg")
nms.out = c("r.XG", "p.G", "p.EG", "p.2df", "p.G.perm", "p.EG.perm", "p.2df.perm")

# run simulations
pflag = 0
for(i in 1:n.iter){
  if(floor(i/100) > pflag){
    #print(paste(Sys.time(), "Iter:", i))
    pflag = pflag + 1
  }
  sdat = gxe.sim.outl(n=n, lambda=lambda, maxt=maxt, bh=bh, bx=bx, be=be, bg=bg, beg=beg)
  rslts[i, nms.in] = c(n, lambda, maxt, bh, bx, be, bg, beg)
  rslts[i, nms.out] = gxe.reg(sdat)
}

# type I error for parametric
tab.s2[rowno.s1, "G.par"] = sum(rslts$p.G < alpha) / n.iter
tab.s2[rowno.s1, "GT.par"] = sum(rslts$p.EG < alpha) / n.iter

# type I error for non-parametric
pdistn = ecdf(rslts[,"p.G.perm"])
tmpX = invfun(pdistn, 0.05, 0.03, 0.07)
tab.s2[rowno.s1, "G.perm"] = sum(rslts$p.G < tmpX) / n.iter

pdistn = ecdf(rslts[,"p.EG.perm"])
tmpX = invfun(pdistn, 0.05, 0.03, 0.07)
tab.s2[rowno.s1, "GT.perm"] = sum(rslts$p.EG < tmpX) / n.iter

# add random outliers to G and event time, but then descretize G
### Continuous covariate, g (gene), binary covariate t (treatment)
gxe.sim.outl = function(n, p.e=0.5, lambda=0.1, maxt, bh, bx, be, bg, beg, n.outl=5 ){
  E = rbinom(n, 1, p.e)
  H <- rnorm(n)
  G <- H*bh + rnorm(n)
  # descretize G?
  X = H*bh + rnorm(n)
  EG = E * G
  dat <- data.frame(X = X, G = G, E = E, EG = EG)
  tmp <- simsurv(dist="exponential", lambdas=lambda,
                 x=dat, betas=c(X=bx, G=bg, E=be, EG=beg), maxt=maxt, )
  sdat = cbind(tmp[,c("eventtime", "status")], dat)

  # add extreme outliers to G
```

```r
  se.o = sd(sdat[,"G"])
  mn.o = mean(sdat[,"G"]) + 5 * se.o
  outlrs = rnorm(n.outl, mn.o, se.o)
  ind = sample(1:n, n.outl)
  sdat[ind, "G"] = outlrs

  # descretize G
  sdat[,"G"] = as.integer(descr(sdat[,"G"], 3))

  # add extreme outliers to eventtime
  ind = sample(1:n, n.outl)
  sdat[ind, "eventtime"] = sdat[ind, "eventtime"] * 5

  return(sdat)
} # end gxe.sim.outl

# Outliers in G and eventtime under the null of beg = 0, bg = 0
n = 329
p.e = .5
maxt = 10
lambda = 0.2
bh = -1.5
bx = 0.2
be = 0.2
bg = 0.0
beg = 0.0

rowno.s1 = rowno.s1 + 1
tab.s2[rowno.s1, "betaG"] = bg
tab.s2[rowno.s1, "betaGT"] = beg
tab.s2[rowno.s1, "outliers"] = 1
tab.s2[rowno.s1, "G.cat"] = 1

# results set-up
nms = c("n", "lambda", "maxt", "bh", "bx", "be", "bg", "beg",
        "p.G", "p.EG", "p.2df", "p.G.perm", "p.EG.perm", "p.2df.perm")
rslts = as.data.frame(matrix(NA, nrow=0, ncol=length(nms)))
names(rslts) = nms
nms.in = c("n", "lambda", "maxt", "bh", "bx", "be", "bg", "beg")
nms.out = c("r.XG", "p.G", "p.EG", "p.2df", "p.G.perm", "p.EG.perm", "p.2df.perm")

# run simulations
pflag = 0
for(i in 1:n.iter){
  if(floor(i/100) > pflag){
    #print(paste(Sys.time(), "Iter:", i))
    pflag = pflag + 1
  }
  sdat = gxe.sim.outl(n=n, lambda=lambda, maxt=maxt, bh=bh, bx=bx, be=be, bg=bg, beg=beg)
  rslts[i, nms.in] = c(n, lambda, maxt, bh, bx, be, bg, beg)
  rslts[i, nms.out] = gxe.reg(sdat)
}
```

```
# type I error for parametric
tab.s2[rowno.s1, "G.par"] = sum(rslts$p.G < alpha) / n.iter
tab.s2[rowno.s1, "GT.par"] = sum(rslts$p.EG < alpha) / n.iter

# type I error for non-parametric
pdistn = ecdf(rslts[,"p.G.perm"])
tmpX = invfun(pdistn, 0.05, 0.03, 0.07)
tab.s2[rowno.s1, "G.perm"] = sum(rslts$p.G < tmpX) / n.iter

pdistn = ecdf(rslts[,"p.EG.perm"])
tmpX = invfun(pdistn, 0.05, 0.03, 0.07)
tab.s2[rowno.s1, "GT.perm"] = sum(rslts$p.EG < tmpX) / n.iter

# Outliers in G and eventtime under the null of beg = 0
n = 329
p.e = .5
maxt = 10
lambda = 0.2
bh = -1.5
bx = 0.2
be = 0.2
bg = 0.2
beg = 0.0

rowno.s1 = rowno.s1 + 1
tab.s2[rowno.s1, "betaG"] = bg
tab.s2[rowno.s1, "betaGT"] = beg
tab.s2[rowno.s1, "outliers"] = 1
tab.s2[rowno.s1, "G.cat"] = 1

# results set-up
nms = c("n", "lambda", "maxt", "bh", "bx", "be", "bg", "beg",
        "p.G", "p.EG", "p.2df", "p.G.perm", "p.EG.perm", "p.2df.perm")
rslts = as.data.frame(matrix(NA, nrow=0, ncol=length(nms)))
names(rslts) = nms
nms.in = c("n", "lambda", "maxt", "bh", "bx", "be", "bg", "beg")
nms.out = c("r.XG", "p.G", "p.EG", "p.2df", "p.G.perm", "p.EG.perm", "p.2df.perm")

# run simulations
pflag = 0
for(i in 1:n.iter){
  if(floor(i/100) > pflag){
    #print(paste(Sys.time(), "Iter:", i))
    pflag = pflag + 1
  }
  sdat = gxe.sim.outl(n=n, lambda=lambda, maxt=maxt, bh=bh, bx=bx, be=be, bg=bg, beg=beg)
  rslts[i, nms.in] = c(n, lambda, maxt, bh, bx, be, bg, beg)
  rslts[i, nms.out] = gxe.reg(sdat)
}

# type I error for parametric
tab.s2[rowno.s1, "G.par"] = sum(rslts$p.G < alpha) / n.iter
tab.s2[rowno.s1, "GT.par"] = sum(rslts$p.EG < alpha) / n.iter
```

```r
# type I error for non-parametric
pdistn = ecdf(rslts[,"p.G.perm"])
tmpX = invfun(pdistn, 0.05, 0.03, 0.07)
tab.s2[rowno.s1, "G.perm"] = sum(rslts$p.G < tmpX) / n.iter

pdistn = ecdf(rslts[,"p.EG.perm"])
tmpX = invfun(pdistn, 0.05, 0.03, 0.07)
tab.s2[rowno.s1, "GT.perm"] = sum(rslts$p.EG < tmpX) / n.iter

# Outliers in G and eventtime under the null of beg != 0
n = 329
p.e = .5
maxt = 10
lambda = 0.2
bh = -1.5
bx = 0.2
be = 0.2
bg = 0.2
beg = 0.2

rowno.s1 = rowno.s1 + 1
tab.s2[rowno.s1, "betaG"] = bg
tab.s2[rowno.s1, "betaGT"] = beg
tab.s2[rowno.s1, "outliers"] = 1
tab.s2[rowno.s1, "G.cat"] = 1

# results set-up
nms = c("n", "lambda", "maxt", "bh", "bx", "be", "bg", "beg",
        "p.G", "p.EG", "p.2df", "p.G.perm", "p.EG.perm", "p.2df.perm")
rslts = as.data.frame(matrix(NA, nrow=0, ncol=length(nms)))
names(rslts) = nms
nms.in = c("n", "lambda", "maxt", "bh", "bx", "be", "bg", "beg")
nms.out = c("r.XG", "p.G", "p.EG", "p.2df", "p.G.perm", "p.EG.perm", "p.2df.perm")

# run simulations
pflag = 0
for(i in 1:n.iter){
  if(floor(i/100) > pflag){
    #print(paste(Sys.time(), "Iter:", i))
    pflag = pflag + 1
  }
  sdat = gxe.sim.outl(n=n, lambda=lambda, maxt=maxt, bh=bh, bx=bx, be=be, bg=bg, beg=beg)
  rslts[i, nms.in] = c(n, lambda, maxt, bh, bx, be, bg, beg)
  rslts[i, nms.out] = gxe.reg(sdat)
}

# type I error for parametric
tab.s2[rowno.s1, "G.par"] = sum(rslts$p.G < alpha) / n.iter
tab.s2[rowno.s1, "GT.par"] = sum(rslts$p.EG < alpha) / n.iter

# type I error for non-parametric
pdistn = ecdf(rslts[,"p.G.perm"])
tmpX = invfun(pdistn, 0.05, 0.03, 0.07)
```

```r
tab.s2[rowno.s1, "G.perm"] = sum(rslts$p.G < tmpX) / n.iter

pdistn = ecdf(rslts[,"p.EG.perm"])
tmpX = invfun(pdistn, 0.05, 0.03, 0.07)
tab.s2[rowno.s1, "GT.perm"] = sum(rslts$p.EG < tmpX) / n.iter

# Supplementary table S1.
knitr::kable(tab.s2, caption = "Type I error and statistical power for main effects and interactions",
             digits=3)
```

Table 3: Type I error and statistical power for main effects and interactions

betaG	betaGT	G.par	GT.par	G.perm	GT.perm	outliers	G.cat
0.0	0.0	0.054	0.047	0.054	0.046	0	0
0.2	0.0	0.990	0.050	0.990	0.048	0	0
0.2	0.2	1.000	0.781	1.000	0.762	0	0
0.0	0.0	0.058	0.045	0.050	0.039	1	0
0.2	0.0	0.822	0.130	0.813	0.120	1	0
0.2	0.2	0.966	0.421	0.964	0.406	1	0
0.0	0.0	0.056	0.050	0.050	0.044	1	1
0.2	0.0	0.908	0.046	0.900	0.046	1	1
0.2	0.2	0.998	0.580	0.998	0.570	1	1

Application to the MAVERICC Clinical Trial

We conducted a TWAS in the MAVERICC clinical trial to identify genes predictive or prognostic of PFS or OS. MV FDR with the BY approach was used to identify discoveries and quantify uncertainty. For comparison, FDR was also computed using the BH method. MAVERICC was a randomized phase II trial comparing first-line treatment with modified leucovorin, 5-fluorouracil, oxaliplatin plus bevacizumab (mFOLFOX6-BV) to treatment with leucovorin, 5-fluorouracil, irinotecan plus bevacizumab (FOLFIRI-BV) in patients with previously untreated metastatic colorectal cancer (A. R. Parikh et al. 2019).

(Not run)

```r
options(stringsAsFactors = FALSE)
# Stratification factors are region and ERCC.Group
# Permutations should be conducted within strata

# read in map for reduced features
fl = "Imputed_expression_reduced_map_v1.csv"
map = read.csv(file=fl)
mlst = vector('list', nrow(map))
for(j in 1:nrow(map)){
  mlst[[j]] = eval(parse(text=map[j, "mapping"]))
}
tmp = unlist(mlst)

map.m = map[map$information < 1, ]
mlst.m = vector('list', nrow(map.m))
for(j in 1:nrow(map.m)){
  mlst.m[[j]] = eval(parse(text=map.m[j, "mapping"]))
}
```

```r
tmp.m = unlist(mlst.m)

map.s = map[map$information == 1, ]
mlst.s = vector('list', nrow(map.s))
for(j in 1:nrow(map.s)){
  mlst.s[[j]] = eval(parse(text=map.s[j, "mapping"]))
}
tmp.s = unlist(mlst.s)


# read in data reduced TWAS oncoarray data -- partition R package was used to reduce data
fl = "Imputed_expression_reduced_v1.csv"
gdat = read.csv(file=fl)

# read in clinical data
fl = "MAV_clinical_P0.csv"
cdat = read.csv(file=fl)

# read in ID linking file
fl = "MAVERICC_linkdat.csv"
link = read.csv(file=fl)

FDRplot1 = function (plotdat, lowerbound, upperbound, ymax = 1, annot = "",
          xpos = 0.8, ypos = 0.8) {
  threshold <- ll <- ul <- fdr <- S <- NULL
  p1 = ggplot2::ggplot(plotdat, aes(x = threshold)) + ggplot2::geom_ribbon(aes(ymin = ll,
  ymax = ul), alpha = 0.4, fill = "grey55") +
  ggplot2::scale_x_continuous(limits = c(lowerbound, upperbound)) +
  ggplot2::scale_y_continuous(limits = c(0, ymax)) +
  ggplot2::theme_bw(base_size = 16) + ggplot2::geom_line(aes(y = fdr),
  color = "grey35", size = 1.5) + ggplot2::xlab(expression(paste("detection threshold (-",
  log[10], "(", alpha, "))"))) + ggplot2::ylab("FDR") + ggplot2::labs(title = "") +
  ggplot2::theme(plot.margin = unit(c(0, 20, 10, 10), "pt")) +
  ggplot2::geom_point(aes(y = fdr)) + ggplot2::geom_text(aes(y = fdr,
  label = S), hjust = -0.2, vjust = -0.2) + ggplot2::annotate("text",
  x = xpos, y = ypos, label = annot, size = 6)
  return(p1)
}

dat.prep = function(cdat){
  # covariates: age, sex, ECOG, KRAS, Ethnicity, resection, nmets
  # covariates
  # Age.at.Baseline..years., Sex, Treatment.Arm.Received, ECOG.Group..0.1.4.,
  # KRAS.Status, Race, Ethnicity
  cdat[,"age"] = cdat[,"Age.at.Baseline..years."]
  cdat[,"sex"] = cdat[,"Sex"]
  cdat[,"treat"] = cdat[,"Treatment.Arm"]
  cdat[,"ECOG"] = ifelse(is.element(cdat[,"ECOG.Group..0.1.4."],"."), 0, cdat[,"ECOG.Group..0.1.4."])
  cdat[,"race"] = "other"
  cdat[,"race"] = ifelse(is.element(cdat[,"Race"], "White"), "white", cdat[,"race"])
  cdat[,"race"] = ifelse(is.element(cdat[,"Ethnicity"], "Hispanic or latino"), "hisp", cdat[,"race"])
  cdat[,"race"] = ifelse(is.element(cdat[,"Race"], "Black or African American"), "black", cdat[,"race"])
  cdat[,"race"] = factor(cdat[,"race"], levels=c("white", "hisp", "black", "other"))
```

```
  cdat[,"nmets"] = cdat[,"No..of.Metastatic.Sites"]
  cdat[,"resection"] = cdat[,"Resection"]
  cdat[,"KRAS"] = ifelse(is.element(cdat[,"KRAS.Status"], "Mutant"), 1, 0)
  cdat[,"time.pfs"] = cdat[, "PFS..Months." ]
  cdat[,"event.pfs"] = cdat[, "PFS.Censoring..1.Event..0.Censored." ] # change to 1: event, 0: censor
  cdat[,"time.os"] = cdat[, "Overall.Survival..Months." ]
  cdat[,"event.os"] = cdat[, "OS.Censoring..1.Event..0.Censored." ] # change to 1: event, 0: censor

  # Binary centered treatment indicator
  cdat[,"treatc"] = ifelse(is.element(cdat$Treatment.Arm, "Arm A (Bevacizumab & FOLFOX6)"), 0, NA)
  cdat[,"treatc"] = ifelse(is.element(cdat$Treatment.Arm, "Arm B (Bevacizumab & FOLFIRI)"), 1, cdat[,"t
  cdat[,"treatc"] = scale(cdat[,"treatc"], scale=FALSE)

  # Response, INV.Best.Overall.Response, 1=CR 2=PR 3=SD 4=PD 5=UE
  cdat[,"resp"] = NA
  cdat[,"resp"] = ifelse(is.element(cdat[,"INV.Best.Overall.Response"], 1:2), 1, cdat[,"resp"])
  cdat[,"resp"] = ifelse(is.element(cdat[,"INV.Best.Overall.Response"], 3:4), 0, cdat[,"resp"])
  cdat[,"side"] = ifelse(is.element(cdat[,"Location.of.Primary.Tumor"], "Left [everything distal from s
  cdat[,"side"] = ifelse(is.element(cdat[,"Location.of.Primary.Tumor"], "Right [right colon and transve

  cdat1 = merge(link[,c("row", "ID")], cdat, by.x="ID", by.y="Patient.ID", all=FALSE)
  names(cdat1)[is.element(names(cdat1), "row")] = "IID"
  covars = c("ID", "IID", "age", "sex", "treat", "treatc", "ECOG", "race", "KRAS", "resection", "nmets"
             "time.pfs", "event.pfs", "time.os", "event.os", "resp", "side", "Region", "ERCC.Group", "m
  gcdat = merge(cdat1[, covars], gdat, by="IID", all=FALSE)
  return(gcdat)
} # end dat.prep

# descretize TWAS features
descr = function(myvar, n.cats){
  probs = (0:n.cats) / n.cats
  qtls = quantile( myvar, probs=probs, na.rm=TRUE )
  myvar.c = cut(myvar, breaks=qtls, include.lowest = TRUE, ordered_result=TRUE)
  return(myvar.c)
}

myanalysis = function(vec){
  iter = as.integer(vec[1])
  gnm = as.character(vec[2])
  covars = c("ID", "IID", "time.pfs", "event.pfs", "time.os", "event.os", "resp", "mystrata",
             "age", "sex", "treat", "ECOG", "race", "KRAS", "resection", "nmets", "resp", "side")
  tmp = gcdat[,c(covars, gnm)]
  g.tmp =  try(as.integer(descr(tmp[,gnm], 3)), silent = TRUE)
  if(length(g.tmp) == 1) g.tmp = tmp[,gnm]
  tmp[,"G"] = g.tmp
  out.nms = c("iter", "gene", "p.g.pfs", "p.tg.pfs", "p.2df.pfs", "p.g.os", "p.tg.os", "p.2df.os", "p.g
  outp = rep(NA, length(out.nms))
  names(outp) = out.nms
  outp["iter"] = iter
  outp["gene"] = gnm
  names(outp) = out.nms

  # PFS
```

```
  tmp1 = na.exclude(tmp[,c("time.pfs", "event.pfs", "mystrata", "age", "sex", "ECOG", "nmets", "side",
  my.surv.pfs = Surv( tmp1[, "time.pfs" ], tmp1[, "event.pfs" ] )
  fit.pfs0 = coxph(my.surv.pfs ~ age + sex + ECOG + nmets + side + KRAS + treat + strata(mystrata), data
  fit.pfs1 = coxph(my.surv.pfs ~ age + sex + ECOG + nmets + side + KRAS + treat + G + strata(mystrata),
  fit.pfs2 = coxph(my.surv.pfs ~ age + sex + ECOG + nmets + side + KRAS + treat + G + treat:G + strata(m
  outp["p.g.pfs"] =  anova(fit.pfs1, test="Chisq")["G", "Pr(>|Chi|)"]
  outp["p.tg.pfs"] =  anova(fit.pfs2, test="Chisq")["treat:G", "Pr(>|Chi|)"]
  outp["p.2df.pfs"] =  anova(fit.pfs0, fit.pfs2)[2,4]

  # OS
  tmp1 = na.exclude(tmp[,c("time.os", "event.os", "mystrata", "age", "sex", "ECOG", "nmets", "side", "K
  my.surv.os = Surv( tmp1[, "time.os" ], tmp1[, "event.os" ] )
  fit.os0 = coxph( my.surv.os ~ age + sex + ECOG + nmets + side + KRAS + treat + strata(mystrata),
                 data=tmp1, method="efron", na.action=na.exclude)
  fit.os1 = coxph( my.surv.os ~ age + sex + ECOG + nmets + side + KRAS + treat + G + strata(mystrata),
                 data=tmp1, method="efron", na.action=na.exclude)
  fit.os2 = coxph( my.surv.os ~ age + sex + ECOG + nmets + side + KRAS + treat + G + treat:G + strata(my
                 data=tmp1, method="efron", na.action=na.exclude)
  outp["p.g.os"] =  anova(fit.os1, test="Chisq")["G", "Pr(>|Chi|)"]
  outp["p.tg.os"] =  anova(fit.os2, test="Chisq")["treat:G", "Pr(>|Chi|)"]
  outp["p.2df.os"] =  anova(fit.os0, fit.os2)[2,4]

  # Response (conditional logistic regression)
  tmp1 = na.exclude(tmp[,c("resp", "age", "sex", "ECOG", "nmets", "side", "KRAS", "treat", "G", "mystra
  fit.resp0 = clogit( resp ~ age + sex + ECOG + nmets + side + KRAS + treat + strata(mystrata),
                    data=tmp1)
  fit.resp1 = clogit( resp ~ age + sex + ECOG + nmets + side + KRAS + treat + G + strata(mystrata),
                data=tmp1)
  fit.resp2 = clogit( resp ~ age + sex + ECOG + nmets + side + KRAS + treat + treat:G + strata(mystrata
                    data=tmp1)
  outp["p.g.resp"] =  anova(fit.resp1)["G", "Pr(>|Chi|)"]
  outp["p.tg.resp"] =  anova(fit.resp2)["TG", "Pr(>|Chi|)"]
  outp["p.2df.resp"] =  anova(fit.resp0, fit.resp2, test="LRT")[2,"P(>|Chi|)"]
  return(outp)
} # end myanalysis()

myanalysis1 = function(gnm){
  covars = c("ID", "IID", "time.pfs", "event.pfs", "time.os", "event.os", "resp", "mystrata",
             "age", "sex", "treat", "ECOG", "race", "KRAS", "resection", "nmets", "resp", "side")
  tmp = gcdat[,c(covars, gnm)]
  g.tmp =  try(as.integer(descr(tmp[,gnm], 3)), silent = TRUE)
  if(length(g.tmp) == 1) g.tmp = tmp[,gnm]
  tmp[,"G"] = g.tmp
  out.nms = c("gene", "g.est.pfs", "g.ll.pfs", "g.ul.pfs", "p.g.pfs", "tg.est.pfs", "tg.ll.pfs", "tg.ul
              "g.est.os", "g.ll.os", "g.ul.os", "p.g.os", "tg.est.os", "tg.ll.os", "tg.ul.os", "p.tg.os
  outp = rep(NA, length(out.nms))
  names(outp) = out.nms
  outp["gene"] = gnm
  names(outp) = out.nms

  # PFS
  tmp1 = na.exclude(tmp[,c("time.pfs", "event.pfs", "mystrata", "age", "sex", "ECOG", "nmets", "side",
  my.surv.pfs = Surv( tmp1[, "time.pfs" ], tmp1[, "event.pfs" ] )
```

```
  fit.pfs0 = coxph(my.surv.pfs ~ age + sex + ECOG + nmets + side + KRAS + treat + strata(mystrata), data
  fit.pfs1 = coxph(my.surv.pfs ~ age + sex + ECOG + nmets + side + KRAS + treat + G + strata(mystrata),
  fit.pfs2 = coxph(my.surv.pfs ~ age + sex + ECOG + nmets + side + KRAS + treat + G + treat:G + strata(m
  outp["p.g.pfs"] =  anova(fit.pfs1, test="Chisq")["G", "Pr(>|Chi|)"]
  outp["p.tg.pfs"] =  anova(fit.pfs2, test="Chisq")["treat:G", "Pr(>|Chi|)"]
  outp["g.est.pfs"] = summary(fit.pfs2)$coefficients["G", "coef"]
  outp["g.ll.pfs"] = log(summary(fit.pfs2)$conf.int["G", "lower .95"])
  outp["g.ul.pfs"] = log(summary(fit.pfs2)$conf.int["G", "upper .95"])
  outp["tg.est.pfs"] = summary(fit.pfs2)$coefficients["treatArm B (Bevacizumab & FOLFIRI):G", "coef"]
  outp["tg.ll.pfs"] = log(summary(fit.pfs2)$conf.int["treatArm B (Bevacizumab & FOLFIRI):G", "lower .95
  outp["tg.ul.pfs"] = log(summary(fit.pfs2)$conf.int["treatArm B (Bevacizumab & FOLFIRI):G", "upper .95

  # OS
  tmp1 = na.exclude(tmp[,c("time.os", "event.os", "mystrata", "age", "sex", "ECOG", "nmets", "side", "KR
  my.surv.os = Surv( tmp1[, "time.os" ], tmp1[, "event.os" ] )
  fit.os0 = coxph( my.surv.os ~ age + sex + ECOG + nmets + side + KRAS + treat + strata(mystrata),
                  data=tmp1, method="efron", na.action=na.exclude)
  fit.os1 = coxph( my.surv.os ~ age + sex + ECOG + nmets + side + KRAS + treat + G + strata(mystrata),
                  data=tmp1, method="efron", na.action=na.exclude)
  fit.os2 = coxph( my.surv.os ~ age + sex + ECOG + nmets + side + KRAS + treat + G + treat:G + strata(my
                  data=tmp1, method="efron", na.action=na.exclude)
  outp["p.g.os"] =  anova(fit.os1, test="Chisq")["G", "Pr(>|Chi|)"]
  outp["p.tg.os"] =  anova(fit.os2, test="Chisq")["treat:G", "Pr(>|Chi|)"]
  outp["g.est.os"] = summary(fit.os2)$coefficients["G", "coef"]
  outp["g.ll.os"] = log(summary(fit.os2)$conf.int["G", "lower .95"])
  outp["g.ul.os"] = log(summary(fit.os2)$conf.int["G", "upper .95"])
  outp["tg.est.os"] = summary(fit.os2)$coefficients["treatArm B (Bevacizumab & FOLFIRI):G", "coef"]
  outp["tg.ll.os"] = log(summary(fit.os2)$conf.int["treatArm B (Bevacizumab & FOLFIRI):G", "lower .95"]
  outp["tg.ul.os"] = log(summary(fit.os2)$conf.int["treatArm B (Bevacizumab & FOLFIRI):G", "upper .95"]

  return(outp)
} # end myanalysis1()

fl = paste("MAV_clinical_P0.csv", sep="")
cdat = read.csv(file=fl)
cdat[,"mystrata"] = paste(cdat[,"Region"], cdat[,"ERCC.Group"], sep=":")
mystrata1 = unique(cdat[,"mystrata"])

for(j in 0:100){
  print(paste("j =", j, Sys.time()))

  # permute IDs of clinical data within Region and ERCC.group groups
  if(j > 0){
    randomizedIDs = rep(NA, nrow(cdat))
    for(k in mystrata1){
      aa = which(is.element(cdat[,"mystrata"], k))
      bb = sample(aa)
      randomizedIDs[aa] = bb
    }
    cdat[, "Patient.ID"] = cdat[randomizedIDs, "Patient.ID"]
  }

  gcdat = dat.prep(cdat)
```

```
  mygns = names(gcdat)[23:ncol(gcdat)]
  nmat =as.data.frame(cbind(1:length(mygns), mygns))
  names(nmat) = c("iter", "gene")

  tmp = apply(nmat, 1, myanalysis)
  rslts = as.data.frame(t(tmp))
  rslts[,"iter"] = as.integer(rslts[,"iter"])
  for(k in 3:ncol(rslts)) rslts[, k] = as.numeric(rslts[, k])

  fl = paste("rslts_p", j,"v8.csv", sep="_")
  write.csv(rslts, file=fl)
}

## compute FDR
j = 0
fl = paste("rslts_p", j,"v8.csv", sep="_")
obs = read.csv(file=fl)
n.perm = 0
perml = vector('list',n.perm)
lno = 1
for(j in 1:100){
  fl = paste("rslts_p", j,"v8.csv", sep="_")
  fexists = file.exists(fl)
  if(!fexists) print(j)
  if(fexists){
    length(perml) = length(perml) + 1
    perml[[lno]] = read.csv(file=fl)
    lno = lno + 1
  }
}

### PFS
mytbl = fdrTbl1(obs$p.g.pfs,perml,"p.g.pfs",nrow(obs),3,6)
mytbl = mytbl[!is.na(mytbl[,"ul"]), ]
mytbl

mytbl = fdrTbl1(obs$p.tg.pfs,perml,"p.tg.pfs",nrow(obs),3,6)
mytbl = mytbl[!is.na(mytbl[,"ul"]), ]
mytbl
# > obs[obs$p.tg.pfs < 10^(-3.8), ]
# X iter               gene   p.g.pfs     p.tg.pfs    p.2df.pfs     p.g.os     p.tg.os
# 769   769  769 ENSG00000196151.6 0.9487188 4.741166e-06 2.832911e-05 0.97484901 0.255904415
# 2998 2998 2998 ENSG00000170955.9 0.1152013 1.006906e-05 1.687249e-05 0.08760474 0.035470584
# 3182 3182 3182 ENSG00000187240.9 0.1543385 6.171507e-05 1.186824e-04 0.86561837 0.001510544
# 4542 4542 4542 ENSG00000108588.9 0.3640449 1.515161e-04 5.061983e-04 0.25691050 0.264667772
# p.2df.os  p.g.resp p.tg.resp p.2df.resp
# 769  0.524197606 0.4873101        NA 0.44097128
# 2998 0.025473627 0.4813216        NA 0.05341362
# 3182 0.006427477 0.3532420        NA 0.29760286
# 4542 0.282309140 0.2307736        NA 0.04510723

# ENSG00000196151.6 WDSUB1
# ENSG00000170955.9 CAVIN3
```

```r
# ENSG00000187240.9 DYNC2H1
# ENSG00000108588.9 CCDC47

obs[,"q.tg.pfs"] = p.adjust(obs[,"p.tg.pfs"], method="BH")
rslts.tg = obs[obs$p.tg.pfs < 10^(-3.8), ]
rslts.tg[,"nlp.tg"] = -log10(rslts.tg[,"p.tg.pfs"])

fl = paste("MAV_clinical_P0.csv", sep="")
cdat = read.csv(file=fl)
cdat[,"mystrata"] = paste(cdat[,"Region"], cdat[,"ERCC.Group"], sep=":")
gcdat = dat.prep(cdat)
gnms = c("ENSG00000196151.6", "ENSG00000170955.9", "ENSG00000187240.9", "ENSG00000108588.9")
for(j in 1:length(gnms)){
  tmp = myanalysis1(gnms[j])
  tmp1 = as.data.frame(matrix(tmp, nrow=1, ncol=length(tmp)))
  names(tmp1) = names(tmp)
  if(j == 1) mycoefs = tmp1 else mycoefs = rbind(mycoefs, tmp1)
}
fl = "Coefs_PFS_tg_v8.csv"
write.csv(mycoefs, file=fl)


mytbl1 = fdrTbl1(obs$p.tg.pfs,perml,"p.tg.pfs",nrow(obs),3,6,correct="BH")
mytbl1 = mytbl1[!is.na(mytbl1[,"ul"]), ]
mytbl1[, c("ll2", "ul2")] = mytbl1[, c("ll", "ul")]
mytbl1 = mytbl1[, c("threshold", "ll2", "ul2")]
mytbl2 = merge(mytbl, mytbl1, by="threshold", all=TRUE)

FDRplot2 = function (plotdat, lowerbound, upperbound, ymax = 1, annot = "",
                     xpos = 0.8, ypos = 0.8) {
  threshold <- ll <- ul <- fdr <- S <- NULL
  p1 = ggplot2::ggplot(plotdat, aes(x = threshold)) + ggplot2::geom_ribbon(aes(ymin = ll,
    ymax = ul), alpha = 0.4, fill = "grey55") +
    ggplot2::geom_ribbon(aes(ymin = ll2, ymax = ul2), alpha = 0.4, fill = "grey75") +
    ggplot2::scale_x_continuous(limits = c(lowerbound, upperbound)) +
    ggplot2::scale_y_continuous(limits = c(0, ymax)) +
    ggplot2::theme_bw(base_size = 16) +
    ggplot2::geom_hline(yintercept=0.05, linetype="dashed", color = "grey40") +
    ggplot2::theme(panel.border = element_blank(), panel.grid.major = element_blank(),
          panel.grid.minor = element_blank(), axis.line = element_line(colour = "black")) +
    ggplot2::geom_line(aes(y = fdr),
    color = "grey45", size = 1.5) +
    ggplot2::xlab(expression(paste("detection threshold (-", log[10], "(p-value))"))) +
    ggplot2::ylab("FDR") + ggplot2::labs(title = "") +
    ggplot2::theme(plot.margin = unit(c(0, 20, 10, 10), "pt")) +
    ggplot2::geom_point(aes(y = fdr)) + ggplot2::geom_text(aes(y = fdr,
    label = S), hjust = -0.2, vjust = -0.2) + ggplot2::annotate("text",
    x = xpos, y = ypos, label = annot, size = 6)

  # ggplot2::xlab(expression(paste("detection threshold (-", log[10], "(", alpha, "))"))) +
  return(p1)
}
p2 = FDRplot2(mytbl2, 3, 5.4)
```

```
fl = "FDRtbl_PFS_tg_by_v8.csv"
write.csv(mytbl2, file=fl)

pdf( "FDRplot_PFS_tg_by_v8.pdf", width=6, height=4 )
grid.arrange(p2, ncol=1, nrow=1)
dev.off()

### OS
mytbl = fdrTbl1(obs$p.g.os,perml,"p.g.os",nrow(obs),3,6) # Not selected by BY approach
mytbl = mytbl[!is.na(mytbl[,"ul"]), ]
mytbl
# obs[obs$p.g.os < 10^(-4.9), ]
#        X iter              gene    p.g.pfs  p.tg.pfs  p.2df.pfs       p.g.os  p.tg.os     p.2df.os p.
# 2358 2358 2358 ENSG00000176209.7 0.08369225 0.1469424 0.07826111 5.086448e-06 0.554221 2.549234e-05 0
# q.g.pfs  q.tg.pfs q.2df.pfs    q.g.os    q.tg.os  q.2df.os  q.g.resp
# 2358 0.8797786 0.9403457 0.8958114 0.0280772 0.9563301 0.1400207 0.9991693
# ENSG00000176209.7 SMIM19

mytbl1 = fdrTbl1(obs$p.g.os,perml,"p.g.os",nrow(obs),3,6,correct="BH")
mytbl1 = mytbl1[!is.na(mytbl1[,"ul"]), ]
mytbl1[, c("ll2", "ul2")] = mytbl1[, c("ll", "ul")]
mytbl1 = mytbl1[, c("threshold", "ll2", "ul2")]
mytbl2 = merge(mytbl, mytbl1, by="threshold", all=TRUE)

fl = "FDRtbl_OS_g_by_v8.csv"
write.csv(mytbl2, file=fl)

p2 = FDRplot2(mytbl2, 3, 5.4)

pdf( "FDRplot_OS_g_by_v8.pdf", width=6, height=4 )
grid.arrange(p2, ncol=1, nrow=1)
dev.off()

mytbl = fdrTbl1(obs$p.tg.os,perml,"p.tg.os",nrow(obs),3,6)
mytbl = mytbl[!is.na(mytbl[,"ul"]), ]
mytbl

mytbl = fdrTbl1(obs$p.2df.os,perml,"p.2df.os",nrow(obs),3,6)
mytbl = mytbl[!is.na(mytbl[,"ul"]), ]
mytbl

obs[,"q.g.pfs"] = p.adjust(obs[,"p.g.pfs"], method="BH")
obs[,"q.tg.pfs"] = p.adjust(obs[,"p.tg.pfs"], method="BH")
obs[,"q.2df.pfs"] = p.adjust(obs[,"p.2df.pfs"], method="BH")
obs[,"q.g.os"] = p.adjust(obs[,"p.g.os"], method="BH")
obs[,"q.tg.os"] = p.adjust(obs[,"p.tg.os"], method="BH")
obs[,"q.2df.os"] = p.adjust(obs[,"p.2df.os"], method="BH")

alpha = 0.05
obs[obs[,"q.g.pfs"] <= alpha, ]
obs[obs[,"q.tg.pfs"] <= alpha, ]
obs[obs[,"q.2df.pfs"] <= alpha, ]
obs[obs[,"q.g.os"] <= alpha, ]
```

```
obs[obs[,"q.tg.os"] <= alpha, ]
obs[obs[,"q.2df.os"] <= alpha, ]

################## Generate KM curves for top genes

# ENSG00000196151.6 WDSUB1 - PFS
# ENSG00000170955.9 CAVIN3 - PFS
# ENSG00000187240.9 DYNC2H1 - PFS
# ENSG00000108588.9 CCDC47 - PFS
# ENSG00000176209.7 SMIM19 - OS

tmp = as.integer(descr(gcdat[, "ENSG00000196151.6"], 3))
gcdat[,"WDSUB1"] = ifelse(is.element(tmp, 1), "low", NA)
gcdat[,"WDSUB1"] = ifelse(is.element(tmp, 2), "mid", gcdat[,"WDSUB1"])
gcdat[,"WDSUB1"] = ifelse(is.element(tmp, 3), "high", gcdat[,"WDSUB1"])
gcdat[,"WDSUB1"] = factor(gcdat[,"WDSUB1"], levels=c("low", "mid", "high"))

tmp = as.integer(descr(gcdat[, "ENSG00000170955.9"], 3))
gcdat[,"CAVIN3"] = ifelse(is.element(tmp, 1), "low", NA)
gcdat[,"CAVIN3"] = ifelse(is.element(tmp, 2), "mid", gcdat[,"CAVIN3"])
gcdat[,"CAVIN3"] = ifelse(is.element(tmp, 3), "high", gcdat[,"CAVIN3"])
gcdat[,"CAVIN3"] = factor(gcdat[,"CAVIN3"], levels=c("low", "mid", "high"))

tmp = gcdat[, "ENSG00000187240.9"]
gcdat[,"DYNC2H1"] = ifelse(tmp < -0.1, "low", NA)
gcdat[,"DYNC2H1"] = ifelse(tmp > -0.1 & tmp < 0, "mid", gcdat[,"DYNC2H1"])
gcdat[,"DYNC2H1"] = ifelse(tmp == 0, "high", gcdat[,"DYNC2H1"])
gcdat[,"DYNC2H1"] = factor(gcdat[,"DYNC2H1"], levels=c("low", "mid", "high"))

tmp = as.integer(descr(gcdat[, "ENSG00000108588.9"], 3))
gcdat[,"CCDC47"] = ifelse(is.element(tmp, 1), "low", NA)
gcdat[,"CCDC47"] = ifelse(is.element(tmp, 2), "mid", gcdat[,"CCDC47"])
gcdat[,"CCDC47"] = ifelse(is.element(tmp, 3), "high", gcdat[,"CCDC47"])
gcdat[,"CCDC47"] = factor(gcdat[,"CCDC47"], levels=c("low", "mid", "high"))

tmp = as.integer(descr(gcdat[, "ENSG00000176209.7"], 3))
gcdat[,"SMIM19"] = ifelse(is.element(tmp, 1), "low", NA)
gcdat[,"SMIM19"] = ifelse(is.element(tmp, 2), "mid", gcdat[,"SMIM19"])
gcdat[,"SMIM19"] = ifelse(is.element(tmp, 3), "high", gcdat[,"SMIM19"])
gcdat[,"SMIM19"] = factor(gcdat[,"SMIM19"], levels=c("low", "mid", "high"))

gcdat[,"treat1"] = gcdat[,"treat"]
gcdat[,"treat"] = ifelse(is.element(gcdat$treat1, "Arm A (Bevacizumab & FOLFOX6)"), "FOLFOX6.bev", gcda
gcdat[,"treat"] = ifelse(is.element(gcdat$treat1, "Arm B (Bevacizumab & FOLFIRI)"), "FOLFIRI.bev", gcda

# read in clinical data
#fl = "/Volumes/ccdept/Biostats/Lenz/Josh/PRKCDBP/Data/dat.m.MAVERICC.Rda"
#load(file=fl) # dat.m.MAVERICC

# merge in PC1, PC2, PC3
#gcdat1 = merge(dat.m.MAVERICC[, c("row", "PC1", "PC2", "PC3")], gcdat, by.x="row", by.y="IID", all=FAL

# KM function
```

```
kmplot1 = function( tmp.dat, ylab, sm1="", sm2="", sm3="", tmax=NULL, bx=NULL, p.logrank, p.lrt ){
  tmp.dat1 = as.data.frame(tmp.dat)
  names(tmp.dat1) = c("time", "event", "Treatment")
  myfit = survfit( Surv(time, event) ~ Treatment, data=tmp.dat1 )

  names(myfit$strata) <- gsub("Treatment=", "", names(myfit$strata))
  summ=summary(myfit)$table
  t1 = "Median Surv (95% CI)"
  ll = ifelse(is.na(round(summ[1, "0.95LCL"], 1)), "--", round(summ[1, "0.95LCL"], 1))
  ul = ifelse(is.na(round(summ[1, "0.95UCL"], 1)), "--", round(summ[1, "0.95UCL"], 1))
  r1 = paste(rownames(summ)[1], "  ", round(summ[1, "median"], 1), "  ", "(", ll, ", ", ul, ")", sep=""
  ll = ifelse(is.na(round(summ[2, "0.95LCL"], 1)), "--", round(summ[2, "0.95LCL"], 1))
  ul = ifelse(is.na(round(summ[2, "0.95UCL"], 1)), "--", round(summ[2, "0.95UCL"], 1))
  r2 = paste(rownames(summ)[2], "  ", round(summ[2, "median"], 1), "  ", "(", ll, ", ", ul, ")", sep=""
  ll = ifelse(is.na(round(summ[3, "0.95LCL"], 1)), "--", round(summ[3, "0.95LCL"], 1))
  ul = ifelse(is.na(round(summ[3, "0.95UCL"], 1)), "--", round(summ[3, "0.95UCL"], 1))
  r3 = paste(rownames(summ)[3], "  ", round(summ[3, "median"], 1), "  ", "(", ll, ", ", ul, ")", sep=""
  # events_n=paste0(summ[,4],'/',summ[,1])
  eq = substitute(expr=paste(sm1, italic(sm2), sm3),
                env = base::list(sm1 = sm1,sm2 = sm2,sm3 = sm3))

  myplot = ggsurvplot(myfit, data=tmp.dat1, submain="",
                      pval = FALSE, conf.int = FALSE, color="black",
                      risk.table = TRUE, fontsize=4, xlab="Time (months)", ylab=ylab,
                      break.x.by = bx, xlim=c(0, tmax),
                      tables.theme = theme_cleantable(),
                      ggtheme = theme(panel.grid.major = element_blank(), panel.grid.minor = element_bla
                                      panel.background = element_blank(), axis.line = element_line(colou
                                      legend.key = element_rect(colour = "transparent", fill = "white")
                                      legend.background = element_rect(fill="transparent")),
                      legend = c(0.1, 0.21),
                      legend.title = "",
                      linetype = c( "solid", "dashed", "dotted"), size=1)
  mt = ifelse(is.null(tmax), max(tmp.dat1$time), tmax)
  myplot$plot = myplot$plot + ggplot2::annotate("text", x=(mt/1.4), y=0.99, label=expression(underline(
  myplot$plot = myplot$plot + ggplot2::annotate("text", x=(mt/1.37), y=0.91, label=r1, size=3.3, hjust=(
  myplot$plot = myplot$plot + ggplot2::annotate("text", x=(mt/1.37), y=0.83, label=r2, size=3.3, hjust=(
  myplot$plot = myplot$plot + ggplot2::annotate("text", x=(mt/1.37), y=0.75, label=r3, size=3.3, hjust=(

  myplot$plot = myplot$plot + ggplot2::annotate("text", x=(mt/5), y=0.99, label=eq, size=4, hjust=0)

  p.lr = paste("logrank p =", p.logrank)
  p.lrt = paste("Cox LRT p =", p.lrt)
  myplot$plot = myplot$plot + ggplot2::annotate("text", x=(mt/1.37), y=0.6, label=p.lr, size=3.3, hjust=
  myplot$plot = myplot$plot + ggplot2::annotate("text", x=(mt/1.37), y=0.52, label=p.lrt, size=3.3, hju

  return( myplot )
} # End kmplot1

# 2 4 panel plots
# A. PFS, WDSUB1, FOLFIRI.bev
aa = is.element(gcdat[,"treat"], "FOLFIRI.bev")
tmp1 = na.exclude(gcdat[aa,c("time.pfs", "event.pfs", "age", "sex", "ECOG", "nmets", "side", "KRAS", "t
```

```
my.surv.pfs = Surv( tmp1[, "time.pfs" ], tmp1[, "event.pfs" ] )
fit.pfs = coxph(my.surv.pfs ~ age + sex + ECOG + nmets + side + KRAS + WDSUB1, data=tmp1, method="efron
summary(fit.pfs)
anova(fit.pfs) # p = 0.00754

survtest <- survdiff(Surv(time.pfs, event.pfs) ~ WDSUB1, data = tmp1)
pchisq(survtest$chisq, df=2, lower.tail=FALSE) # p = 0.002379571

p.logrank = "0.0024"
p.lrt = "0.0075"
p.WDSUB1.pfs.ff = kmplot1(tmp1[,c("time.pfs","event.pfs", "WDSUB1")],
                       ylab="PFS", sm1="FOLFIRI.bev -- ", sm2="WDSUB1", sm3="", tmax=40, bx=10, p.logran

# B. PFS, WDSUB1, FOLFOX6.bev
aa = is.element(gcdat[,"treat"], "FOLFOX6.bev")
tmp1 = na.exclude(gcdat[aa,c("time.pfs", "event.pfs", "age", "sex", "ECOG", "nmets", "side", "KRAS", "t
my.surv.pfs = Surv( tmp1[, "time.pfs" ], tmp1[, "event.pfs" ] )
fit.pfs = coxph(my.surv.pfs ~ age + sex + ECOG + nmets + side + KRAS + WDSUB1, data=tmp1, method="efron
summary(fit.pfs)
anova(fit.pfs) # p = 0.00318

survtest <- survdiff(Surv(time.pfs, event.pfs) ~ WDSUB1, data = tmp1)
pchisq(survtest$chisq, df=2, lower.tail=FALSE) # p = 0.01376756

p.logrank = "0.014"
p.lrt = "0.0032"
p.WDSUB1.pfs.fx = kmplot1(tmp1[,c("time.pfs","event.pfs", "WDSUB1")],
                       ylab="PFS", sm1="FOLFOX6.bev -- ", sm2="WDSUB1", sm3="", tmax=40, bx=10, p.logran

# C. PFS, CAVIN3, FOLFIRI.bev
aa = is.element(gcdat[,"treat"], "FOLFIRI.bev")
tmp1 = na.exclude(gcdat[aa,c("time.pfs", "event.pfs", "age", "sex", "ECOG", "nmets", "side", "KRAS", "t
my.surv.pfs = Surv( tmp1[, "time.pfs" ], tmp1[, "event.pfs" ] )
fit.pfs = coxph(my.surv.pfs ~ age + sex + ECOG + nmets + side + KRAS + CAVIN3, data=tmp1, method="efron
summary(fit.pfs)
anova(fit.pfs) # p = 0.09992

survtest <- survdiff(Surv(time.pfs, event.pfs) ~ CAVIN3, data = tmp1)
pchisq(survtest$chisq, df=2, lower.tail=FALSE) # p = 0.2252713

p.logrank = "0.23"
p.lrt = "0.10"
p.CAVIN3.pfs.ff = kmplot1(tmp1[,c("time.pfs","event.pfs", "CAVIN3")],
                          ylab="PFS", sm1="FOLFIRI.bev -- ", sm2="CAVIN3", sm3="", tmax=40, bx=10, p.log

# D. PFS, CAVIN3, FOLFOX6.bev
aa = is.element(gcdat[,"treat"], "FOLFOX6.bev")
tmp1 = na.exclude(gcdat[aa,c("time.pfs", "event.pfs", "age", "sex", "ECOG", "nmets", "side", "KRAS", "t
my.surv.pfs = Surv( tmp1[, "time.pfs" ], tmp1[, "event.pfs" ] )
fit.pfs = coxph(my.surv.pfs ~ age + sex + ECOG + nmets + side + KRAS + CAVIN3, data=tmp1, method="efron
summary(fit.pfs)
anova(fit.pfs) # p = 3.647e-05
```

```
survtest <- survdiff(Surv(time.pfs, event.pfs) ~ CAVIN3, data = tmp1)
pchisq(survtest$chisq, df=2, lower.tail=FALSE) # p = 6.316307e-05

p.logrank = "6.3e-5"
p.lrt = "3.6e-5"
p.CAVIN3.pfs.fx = kmplot1(tmp1[,c("time.pfs","event.pfs", "CAVIN3")],
                          ylab="PFS", sm1="FOLFOX6.bev -- ", sm2="CAVIN3", sm3="", tmax=40, bx=10, p.log

# E. PFS, DYNC2H1, FOLFIRI.bev
aa = is.element(gcdat[,"treat"], "FOLFIRI.bev")
tmp1 = na.exclude(gcdat[aa,c("time.pfs", "event.pfs", "age", "sex", "ECOG", "nmets", "side", "KRAS", "tr
my.surv.pfs = Surv( tmp1[, "time.pfs" ], tmp1[, "event.pfs" ] )
fit.pfs = coxph(my.surv.pfs ~ age + sex + ECOG + nmets + side + KRAS + DYNC2H1, data=tmp1, method="efron
summary(fit.pfs)
anova(fit.pfs) # p = 0.00443

survtest <- survdiff(Surv(time.pfs, event.pfs) ~ DYNC2H1, data = tmp1)
pchisq(survtest$chisq, df=2, lower.tail=FALSE) # p = 0.02946822

p.logrank = "0.029"
p.lrt = "0.0044"
p.DYNC2H1.pfs.ff = kmplot1(tmp1[,c("time.pfs","event.pfs", "DYNC2H1")],
                           ylab="PFS", sm1="FOLFIRI.bev -- ", sm2="DYNC2H1", sm3="", tmax=40, bx=10, p.lo

# F. PFS, DYNC2H1, FOLFOX6.bev
aa = is.element(gcdat[,"treat"], "FOLFOX6.bev")
tmp1 = na.exclude(gcdat[aa,c("time.pfs", "event.pfs", "age", "sex", "ECOG", "nmets", "side", "KRAS", "tr
my.surv.pfs = Surv( tmp1[, "time.pfs" ], tmp1[, "event.pfs" ] )
fit.pfs = coxph(my.surv.pfs ~ age + sex + ECOG + nmets + side + KRAS + DYNC2H1, data=tmp1, method="efron
summary(fit.pfs)
anova(fit.pfs) # p = 0.1761

survtest <- survdiff(Surv(time.pfs, event.pfs) ~ DYNC2H1, data = tmp1)
pchisq(survtest$chisq, df=2, lower.tail=FALSE) # p = 0.1287833

p.logrank = "0.13"
p.lrt = "0.18"
p.DYNC2H1.pfs.fx = kmplot1(tmp1[,c("time.pfs","event.pfs", "DYNC2H1")],
                           ylab="PFS", sm1="FOLFOX6.bev -- ", sm2="DYNC2H1", sm3="", tmax=40, bx=10, p.lo

# G. PFS, CCDC47, FOLFIRI.bev
aa = is.element(gcdat[,"treat"], "FOLFIRI.bev")
tmp1 = na.exclude(gcdat[aa,c("time.pfs", "event.pfs", "age", "sex", "ECOG", "nmets", "side", "KRAS", "tr
my.surv.pfs = Surv( tmp1[, "time.pfs" ], tmp1[, "event.pfs" ] )
fit.pfs = coxph(my.surv.pfs ~ age + sex + ECOG + nmets + side + KRAS + CCDC47, data=tmp1, method="efron
summary(fit.pfs)
anova(fit.pfs) # p = 0.06618

survtest <- survdiff(Surv(time.pfs, event.pfs) ~ CCDC47, data = tmp1)
pchisq(survtest$chisq, df=2, lower.tail=FALSE) # p = 0.1403054

p.logrank = "0.14"
p.lrt = "0.066"
```

```
p.CCDC47.pfs.ff = kmplot1(tmp1[,c("time.pfs","event.pfs", "CCDC47")],
                          ylab="PFS", sm1="FOLFIRI.bev -- ", sm2="CCDC47", sm3="", tmax=40, bx=10, p.log

# H. PFS, CCDC47, FOLFOX6.bev
aa = is.element(gcdat[,"treat"], "FOLFOX6.bev")
tmp1 = na.exclude(gcdat[aa,c("time.pfs", "event.pfs", "age", "sex", "ECOG", "nmets", "side", "KRAS", "t
my.surv.pfs = Surv( tmp1[, "time.pfs" ], tmp1[, "event.pfs" ] )
fit.pfs = coxph(my.surv.pfs ~ age + sex + ECOG + nmets + side + KRAS + CCDC47, data=tmp1, method="efron
summary(fit.pfs)
anova(fit.pfs) # p = 0.0008368

survtest <- survdiff(Surv(time.pfs, event.pfs) ~ CCDC47, data = tmp1)
pchisq(survtest$chisq, df=2, lower.tail=FALSE) # p = 0.001409881

p.logrank = "0.0014"
p.lrt = "0.00084"
p.CCDC47.pfs.fx = kmplot1(tmp1[,c("time.pfs","event.pfs", "CCDC47")],
                          ylab="PFS", sm1="FOLFOX6.bev -- ", sm2="CCDC47", sm3="", tmax=40, bx=10, p.log

# ENSG00000196151.6 WDSUB1 - PFS
# ENSG00000170955.9 CAVIN3 - PFS
# ENSG00000187240.9 DYNC2H1 - PFS
# ENSG00000108588.9 CCDC47 - PFS

splots <- list()
splots[[1]] = p.WDSUB1.pfs.ff
splots[[2]] = p.CAVIN3.pfs.ff
splots[[3]] = p.DYNC2H1.pfs.ff
splots[[4]] = p.CCDC47.pfs.ff

splots[[5]] = p.WDSUB1.pfs.fx
splots[[6]] = p.CAVIN3.pfs.fx
splots[[7]] = p.DYNC2H1.pfs.fx
splots[[8]] = p.CCDC47.pfs.fx

res <- arrange_ggsurvplots(splots, print = TRUE,
                           ncol = 2, nrow = 4, risk.table.height = 0.23)
ggsave("KM_MAVERICC_TWAS_PFS_Table1_v7.pdf", res, width=11, height=16)

# KM function
kmplot1 = function( tmp.dat, ylab, sm1="", sm2="", sm3="", tmax=NULL, bx=NULL, p.logrank, p.lrt ){
  tmp.dat1 = as.data.frame(tmp.dat)
  names(tmp.dat1) = c("time", "event", "Treatment")
  myfit = survfit( Surv(time, event) ~ Treatment, data=tmp.dat1 )

  names(myfit$strata) <- gsub("Treatment=", "", names(myfit$strata))
  summ=summary(myfit)$table
  t1 = "Median Surv (95% CI)"
  medn = ifelse(is.na(summ[1, "median"]), "--", round(summ[1, "median"], 1))
  ll = ifelse(is.na(round(summ[1, "0.95LCL"], 1)), "--", round(summ[1, "0.95LCL"], 1))
  ul = ifelse(is.na(round(summ[1, "0.95UCL"], 1)), "--", round(summ[1, "0.95UCL"], 1))
  r1 = paste(rownames(summ)[1], "  ", medn, "  ", "(", ll, ", ", ul, ")", sep="" )
```

```
  medn = ifelse(is.na(summ[2, "median"]), "--", round(summ[2, "median"], 1))
  ll = ifelse(is.na(round(summ[2, "0.95LCL"], 1)), "--", round(summ[2, "0.95LCL"], 1))
  ul = ifelse(is.na(round(summ[2, "0.95UCL"], 1)), "--", round(summ[2, "0.95UCL"], 1))
  r2 = paste(rownames(summ)[2], "  ", medn, "  ", "(", ll, ", ", ul, ")", sep="" )

  medn = ifelse(is.na(summ[3, "median"]), "--", round(summ[3, "median"], 1))
  ll = ifelse(is.na(round(summ[3, "0.95LCL"], 1)), "--", round(summ[3, "0.95LCL"], 1))
  ul = ifelse(is.na(round(summ[3, "0.95UCL"], 1)), "--", round(summ[3, "0.95UCL"], 1))
  r3 = paste(rownames(summ)[3], "  ", medn, "  ", "(", ll, ", ", ul, ")", sep="" )
  # events_n=paste0(summ[,4],'/',summ[,1])
  eq = substitute(expr=paste(sm1, italic(sm2), sm3),
                env = base::list(sm1 = sm1,sm2 = sm2,sm3 = sm3))

  myplot = ggsurvplot(myfit, data=tmp.dat1, submain="",
                      pval = FALSE, conf.int = FALSE, color="black",
                      risk.table = TRUE, fontsize=4, xlab="Time (months)", ylab=ylab,
                      break.x.by = bx, xlim=c(0, tmax),
                      tables.theme = theme_cleantable(),
                      ggtheme = theme(panel.grid.major = element_blank(), panel.grid.minor = element_bla
                                      panel.background = element_blank(), axis.line = element_line(colou
                                      legend.key = element_rect(colour = "transparent", fill = "white")
                                      legend.background = element_rect(fill="transparent")),
                      legend = c(0.1, 0.18),
                      legend.title = "",
                      linetype = c( "solid", "dashed", "dotted"), size=1)
  mt = ifelse(is.null(tmax), max(tmp.dat1$time), tmax)
  myplot$plot = myplot$plot + ggplot2::annotate("text", x=(mt/1.4), y=0.99, label=expression(underline(
  myplot$plot = myplot$plot + ggplot2::annotate("text", x=(mt/1.37), y=0.91, label=r1, size=3.3, hjust=
  myplot$plot = myplot$plot + ggplot2::annotate("text", x=(mt/1.37), y=0.83, label=r2, size=3.3, hjust=
  myplot$plot = myplot$plot + ggplot2::annotate("text", x=(mt/1.37), y=0.75, label=r3, size=3.3, hjust=

  myplot$plot = myplot$plot + ggplot2::annotate("text", x=(mt/5), y=0.99, label=eq, size=4, hjust=0)

  p.lr = paste("logrank p =", p.logrank)
  p.lrt = paste("Cox LRT p =", p.lrt)
  myplot$plot = myplot$plot + ggplot2::annotate("text", x=0.0, y=0.45, label=p.lr, size=3.3, hjust=0)
  myplot$plot = myplot$plot + ggplot2::annotate("text", x=0.0, y=0.37, label=p.lrt, size=3.3, hjust=0)

  return( myplot )
} # End kmplot1

###### OS, SMIM19, SMAD1
# A. OS, SMIM19, FOLFIRI.bev
aa = is.element(gcdat[,"treat"], "FOLFIRI.bev")
tmp1 = na.exclude(gcdat[aa,c("time.os", "event.os", "age", "sex", "ECOG", "nmets", "side", "KRAS", "trea
my.surv.os = Surv( tmp1[, "time.os" ], tmp1[, "event.os" ] )
fit.os = coxph(my.surv.os ~ age + sex + ECOG + nmets + side + KRAS + SMIM19, data=tmp1, method="efron",
summary(fit.os)
anova(fit.os) # p = 0.003607

survtest <- survdiff(Surv(time.os, event.os) ~ SMIM19, data = tmp1)
pchisq(survtest$chisq, df=2, lower.tail=FALSE) # p = 0.004156277
```

```
p.logrank = "0.0042"
p.lrt = "0.0036"
p.SMIM19.os.ff = kmplot1(tmp1[,c("time.os","event.os", "SMIM19")],
                          ylab="OS", sm1="FOLFIRI.bev -- ", sm2="SMIM19", sm3="", tmax=45, bx=10, p.log

# B. OS, SMIM19, FOLFOX6.bev
aa = is.element(gcdat[,"treat"], "FOLFOX6.bev")
tmp1 = na.exclude(gcdat[aa,c("time.os", "event.os", "age", "sex", "ECOG", "nmets", "side", "KRAS", "tre
my.surv.os = Surv( tmp1[, "time.os" ], tmp1[, "event.os" ] )
fit.os = coxph(my.surv.os ~ age + sex + ECOG + nmets + side + KRAS + SMIM19, data=tmp1, method="efron",
summary(fit.os)
anova(fit.os) # p = 0.004351

survtest <- survdiff(Surv(time.os, event.os) ~ SMIM19, data = tmp1)
pchisq(survtest$chisq, df=2, lower.tail=FALSE) # p = 0.004303152

p.logrank = "0.0043"
p.lrt = "0.0044"
p.SMIM19.os.fx = kmplot1(tmp1[,c("time.os","event.os", "SMIM19")],
                          ylab="OS", sm1="FOLFOX6.bev -- ", sm2="SMIM19", sm3="", tmax=45, bx=10, p.log

# ENSG00000176209.7 SMIM19 - OS

splots <- list()
splots[[1]] = p.SMIM19.os.ff
splots[[2]] = p.SMIM19.os.fx

res <- arrange_ggsurvplots(splots, print = TRUE,
                           ncol = 1, nrow = 2, risk.table.height = 0.19)
ggsave("KM_MAVERICC_TWAS_OS_SMIM19_v7.pdf", res, width=6, height=11)

sessionInfo()
```

Benjamini, Yoav, and Yosef Hochberg. 1995. “Controlling the False Discovery Rate: A Practical and Powerful Approach to Multiple Testing.” Journal Article. *Journal of the Royal Statistical Society: Series B (Methodological)* 57.1: 289–300.

Benjamini, Yoav, and Daniel Yekutieli. 2005. “False Discovery Rate Adjusted Multiple Confidence Intervals for Selected Parameters.” Journal Article. *Journal of the American Statistical Association* 100 (469): 71–81.

Millstein, Joshua, Francesca Battaglin, Hiroyuki Arai, Wu Zhang, Priya Jayachandran, Shivani Soni, Christoph Parikh Aparna R. And Mancao, and Heinz-Josef Lenz. 2022. *Fdrci: FDR Confidence Interval Selection and Adjustment for Large-Scale Hypothesis Testing. Bioinformatics Advances.*

Parikh, Aparna R, Fa-Chyi Lee, Linda Yau, Han Koh, James Knost, Edith P Mitchell, Ivan Bosanac, et al. 2019. “MAVERICC, a Randomized, Biomarker-Stratified, Phase Ii Study of mFOLFOX6-Bevacizumab Versus Folfiri-Bevacizumab as First-Line Chemotherapy in Metastatic Colorectal Cancer.” *Clinical Cancer Research* 25 (10). AACR: 2988–95.
